# *p*TSA-catalyzed synthesis of functionalized chromeno[2,3-*d*]pyrimidine/chromeno[4,3-*b*]chromene derivatives *via* one-pot three component reaction: mechanistic insights and variable temperature NMR studies to investigate restricted bond rotation

**DOI:** 10.1039/d5ra06198a

**Published:** 2025-10-06

**Authors:** Yogesh Bhaskar Singh Tanwer, Suman Sourabh, Sabyasachi Bhunia, Sanchari Pal, Debjit Das

**Affiliations:** a Department of Chemistry, Central University of Jharkhand Ranchi Jharkhand India sabyasachi.bhunia@cuj.ac.in; b Department of Chemistry, Triveni Devi Bhalotia College Raniganj India debjitofchem@gmail.com

## Abstract

In this study, an efficient and convenient three-component cyclization protocol is presented for the development of synthetically important chromeno[2,3-*d*]pyrimidine/chromeno[4,3-*b*]chromene derivatives *via p*TSA-catalyzed reaction of substituted salicylaldehyde, 1,3-dimethylbarbituric acid/4-hydroxycoumarin, and electron-rich arenes at 80 °C in the presence of ethanol. In addition, variable temperature (VT) ^1^H &^13^C NMR spectroscopy is used to study the dynamic Csp^2^–Csp^3^ bond between the electron-rich aryl group and the benzylic sp^3^-carbon. Furthermore, our development is simple and economical, tolerates many functional groups, works with a wide variety of substrates, produces exceptional yields, does not require column chromatography, and allows for scalable synthesis—all of which support the basic principles of green chemistry.

## Abstract 2

An efficient method is presented for the synthesis of chromeno[2,3-*d*]pyrimidine/chromeno[4,3-*b*]chromene derivatives *via p*TSA-catalyzed cyclization of 1,3-dimethylbarbituric acid/4-hydroxycoumarin, electron-rich arenes, and various salicylaldehydes.

## Graphical abstract

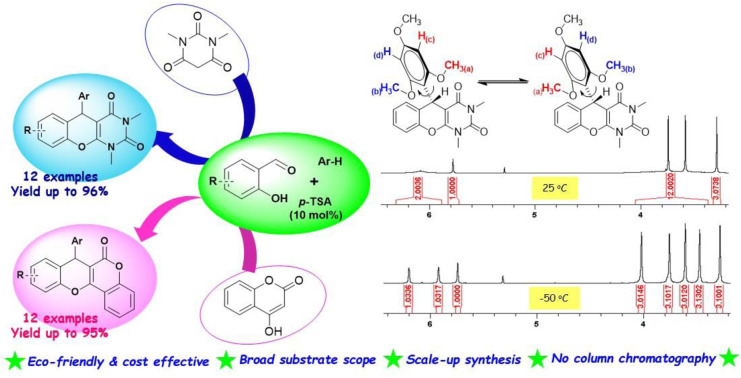

## Introduction

Multi-component reactions (MCRs) have long been considered as an important methodological tool in medicinal and pharmaceutical chemistry. Compared to the conventional organic transformations, MCRs offer a faster and more efficient method for developing chemical libraries, particularly in the drug discovery process.^1^ Additionally, MCRs are becoming more popular than the methods used in traditional organic synthesis due to their typical eco-friendly transformations, atom/step economy, shortened reaction time, and high levels of regio- and chemo-selectivity with excellent yields. Since there is no need to isolate and purify the reaction intermediates, multicomponent reactions are highly beneficial in synthesizing privileged scaffolds while saving money, time, labor, and energy. In recent advances, the development of green and novel synthetic approaches through MCRs has emerged for the construction of complex molecules from readily available starting precursors in a single operation that minimizes the unnecessary environmental impact.^2^ One of the primary issues with organic synthesis is the utilization of costly and hazardous metal catalysts, as well as volatile and toxic organic solvents. Thus, the establishment of novel metal-free MCRs and exploration for more environmentally benign distinct catalytic systems are receiving substantial attention in the areas of medicinal chemistry, pharmaceutical chemistry, synthetic organic chemistry, and fine chemical synthesis.^3^ In this regard, the use of *p*-toluenesulfonic acid (*p*TSA) as a catalyst in metal free chemical synthesis has expanded rapidly due to its numerous advantages, like easy availability, low cost, operational simplicity, good selectivity, non-toxicity, and high yields with ease of isolation of the desired products from the reaction mixture.^4^

**Fig. 1:**
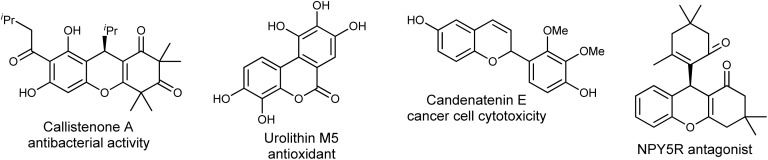
Representative examples of biologically active chromene moieties.

The presence of the chromene moiety in the pharmacophores of various biologically active substances has drawn a lot of interest (Fig. 1).^5^ Chromenes and their structural equivalents are generally physiologically active in nature, have intriguing therapeutic properties and are found in drugs, natural products, and plants, including various fruits and vegetables.^6^

**Fig. 2:**
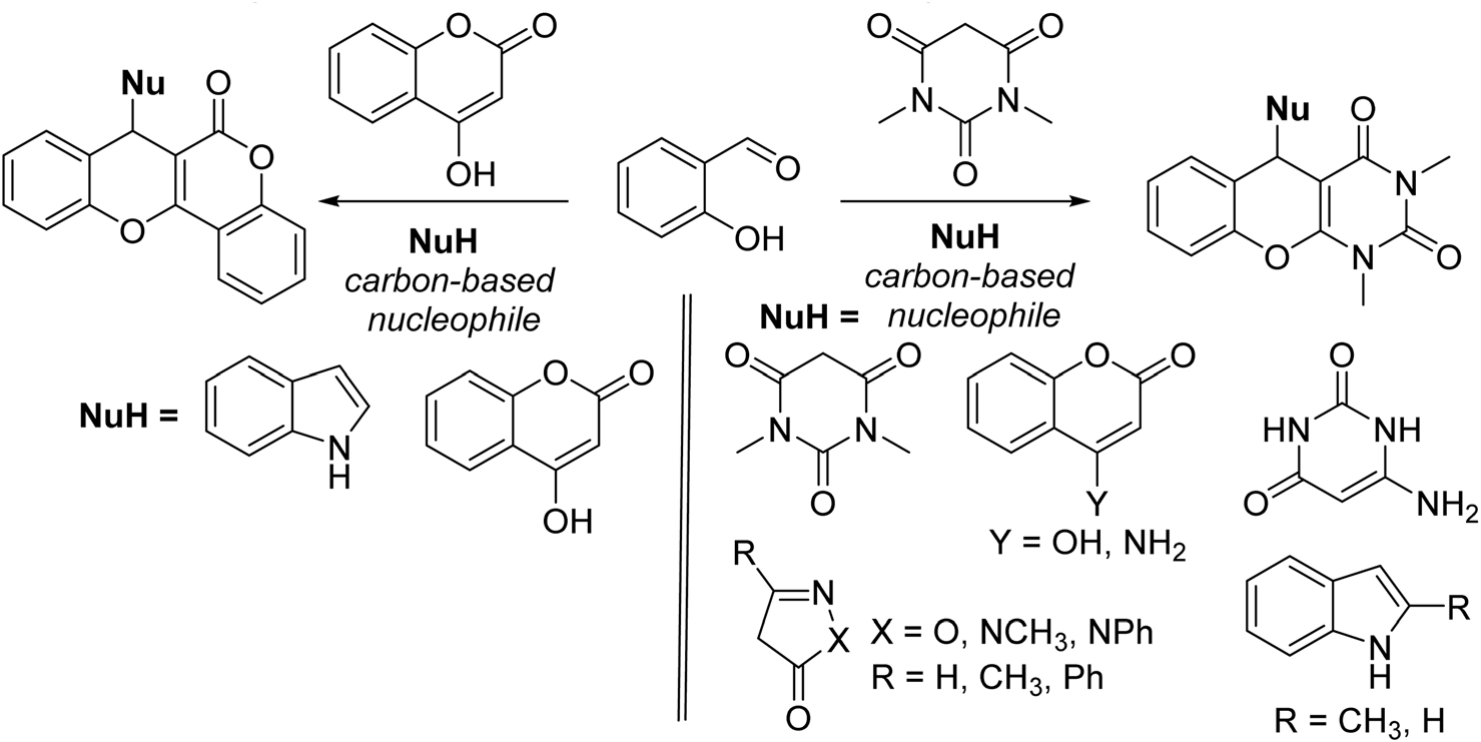
Synthesis of chromeno[2,3-*d*]pyrimidines and chromeno[4,3-*b*]chromenes with some representative carbon-based nucleophiles.

On the other hand, the compounds containing barbituric acids/coumarin analogues are ubiquitous to various important naturally occurring molecules and drugs and have been used in clinical practice.^7,8^ A molecular framework that builds up chromene and other bioactive analogues may combine the characteristics of both, and the combination of the two heterocyclic moieties in one nucleus may produce a specific class of substances that are valuable from a biological perspective. In this context, chromeno[2,3-*d*]pyrimidines & chromeno[4,3-*b*]chromene derivatives are pharmacologically & biologically very important active scaffolds. A comprehensive literature survey suggested that, the pharmacological characteristics of both chromeno[2,3-*d*]pyrimidine and chromeno[4,3-*b*]chromene derivatives are quite diverse including antithrombotic, antioxidant, HIV-1 IN inhibitors, antifungal, antibacterial, antimicrobial, antiplatelet, analgesic, antigenotoxic activities and photophysical properties.^9^ Hence, there are several reports for the synthesis of chromeno[2,3-*d*]pyrimidines and chromeno[4,3-*b*]chromenes *via* one-pot, three-component coupling reactions of a carbon-based nucleophile,^10^ salicylaldehyde, and 1,3-dimethylbarbituric acid/4-hydroxycoumarin using different metal-containing promoters, acids and bases (Fig. 2).^7,11^

Even though these approaches have their own benefits, they also come with various drawbacks, including narrow substrate scope, the need for costly and not easily accessible catalyst systems, metal catalysts, and a tedious work-up procedure. These factors can lead to higher costs in extraction and purification, along with increased waste production. It has also been confirmed by the literature that no general protocol has been made until now on the direct utilization of electron-rich arenes for the synthesis of chromeno[2,3-*d*]pyrimidines and chromeno[4,3-*b*]chromenes with an arene located at the 5/7 positions. This provokes us to develop a novel and efficient method for the synthesis of selective arene functionalized chromenes. Considering the significance of chromene derivatives and our continuous interest in the development of green and eco-friendly processes,^12^ here we report a convenient and efficient *p*-TSA catalyzed synthesis of various substituted chromeno[2,3-*d*]pyrimidines/chromeno[4,3-*b*]chromenes from a one-pot multicomponent reaction involving salicylaldehydes, 1,3-dimethylbarbituric acid/4-hydroxycoumarin, and electron-rich arenes. The present method has many advantages, including operational simplicity, high tolerance towards functional groups, excellent yields, no requirement for column chromatographic separation, environmental friendliness, and the ability to be readily scaled up for large-scale synthesis.

## Results and discussion

We conducted an extensive screening test using several Brønsted acids and solvents, based on the hypothesis that the formation of chromeno[2,3-*d*]pyrimidines would occur through the one-step three-component reaction. Initially, salicylaldehyde 1a (0.25 mmol), 1,3,5-trimethoxybenzene 2a (0.25 mmol), and 1,3-dimethylbabituric acid 3a (0.25 mmol) were stirred at room temperature in the presence of *p*TSA (20 mol%) and ethanol (3 mL); however, even after 24 h, a trace amount of product 4a was generated. Then the reaction was performed under 80 °C for 12 h and the desired product was isolated in 88% yield (Table 1, entry 1). The yield of the reaction was improved up to 95% by reducing the amount of solvent and catalyst loading (Table 1, entries 2 and 4). We observed that in the presence of 10 mol% of *p*TSA in 2 mL of ethanol, a better result was obtained (Table 1, entry 4) with respect to the other tested organic solvents (Table 1, entries 5–7). To further improve the yield, different Brønsted acids have also been examined (Table 1, entries 8–11) and to our delight, the desired product 4a was precipitated out in all cases with lower yields (65% to 86%). The reaction was also attempted in the absence of any Brønsted acids, but it was unable to produce any desired products even after prolonged reaction time exposure (Table 1, entries 12). Hence, using 10 mol% of *p*TSA in 2 mL of ethanol under 80 °C are the optimal conditions for this three-component coupling reaction.

**Table 1:** Optimization of reaction conditions for 4*H*-chromene derivative^a^

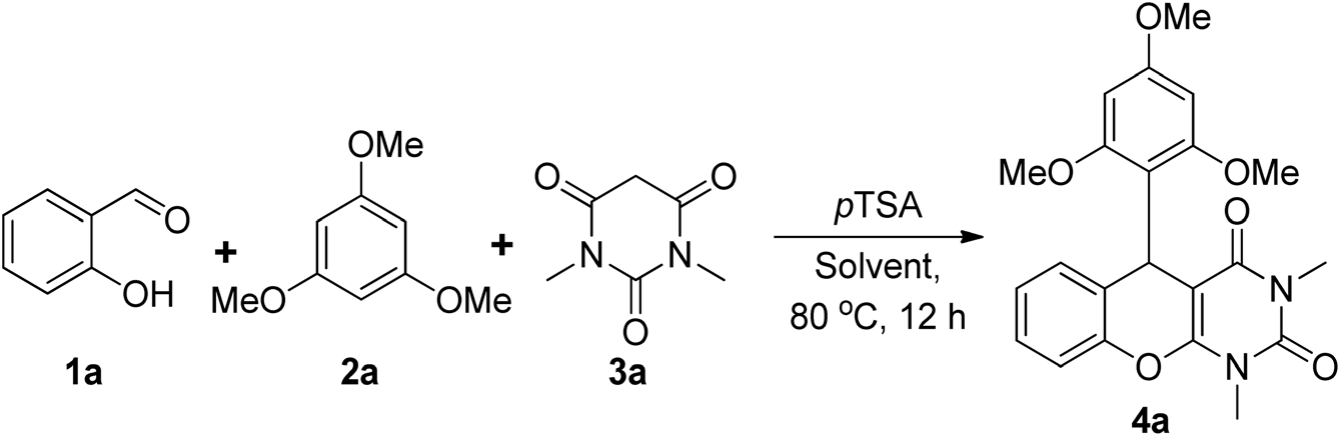			
S. No.	Brønsted acids (%)	Solvent (mL)	Yield of 4a (%)
01	*p*TSA (20)	EtOH (3)	88^b^
02	*p*TSA (10)	EtOH (3)	91^b^
03	*p*TSA (10)	EtOH (1)	77^b^
04	***p*TSA (10)**	**EtOH (2)**	**95^b^**
05	*p*TSA (10)	THF (2)	65
06	*p*TSA (10)	DCE (2)	50
07	*p*TSA (10)	CH_3_CN (2)	69
08	CH_3_CO_2_H (10)	EtOH (2)	72
09	CH_3_SO_3_H (10)	EtOH (2)	65
10	CF_3_CO_2_H (10)	EtOH (2)	68
11	Polyphosphoric acid (10)	EtOH (2)	86^b^
12	—	EtOH (2)	No reaction

a All reactions were carried out in 0.25 mmol scale.

b Isolated yield after filtration.

**Fig. 3:**
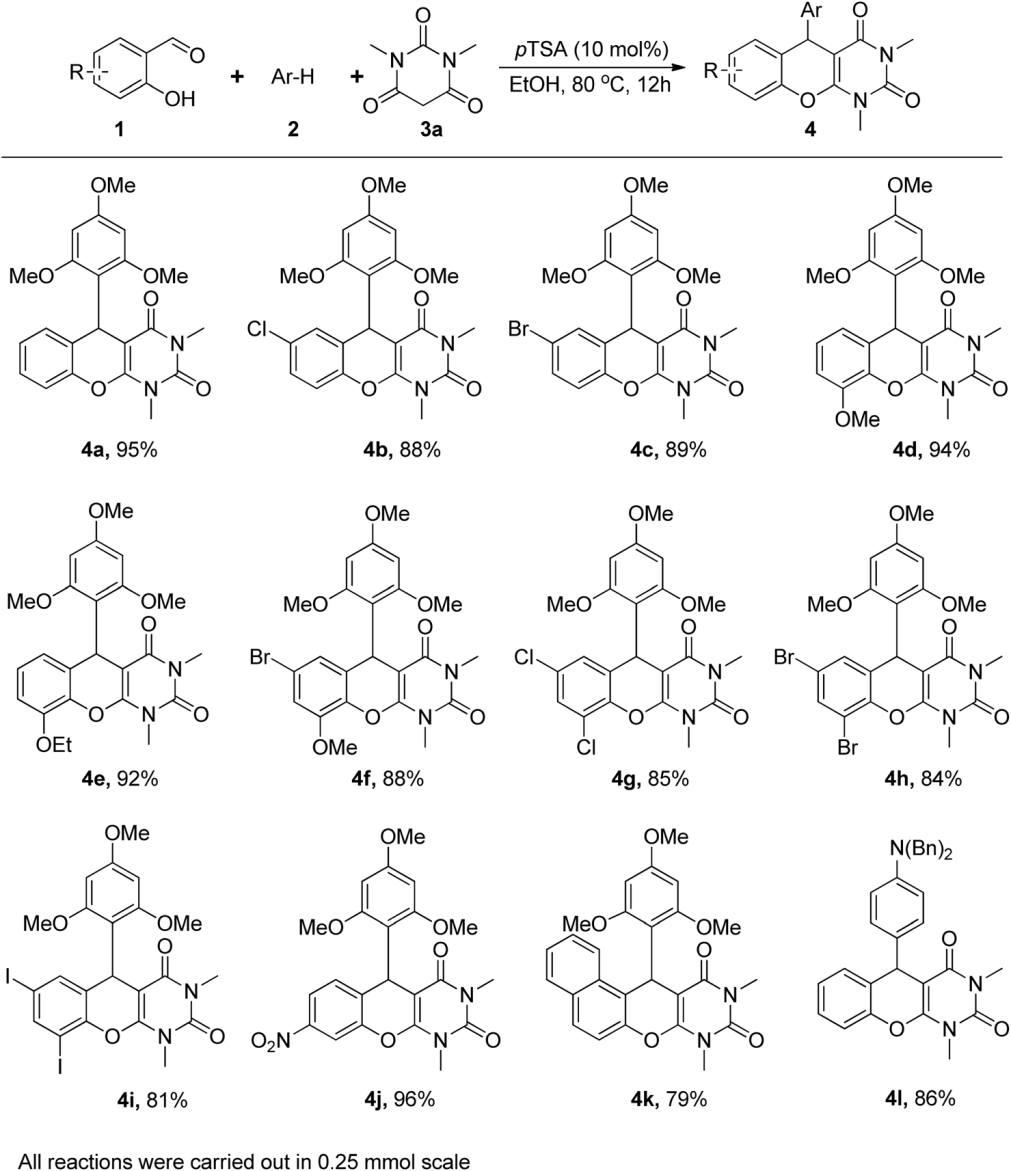
*p*-TSA catalysed synthesis of chromeno[2,3-*d*]pyrimidine: substrate scope.

Once we have the optimal reaction conditions, we explored the potential substrate scope of these three-component coupling reactions and the outcomes are depicted in Fig. 3. Typically, a mixture of 2-hydroxybenzaldehyde (0.25 mmol), 1,3,5-trimethoxybenzene (0.25 mmol), and 1,3-dimethylbarbituric acid was heated for 12 h at 80 °C in the presence of 10 mol% of *p*TSA in 2.0 mL of EtOH, which afforded a library of chromeno[2,3-*d*]pyrimidines in good to excellent yields. Initially, in the presence of 1,3-dimethylbarbituric acid and electron-rich 1,3,5-trimethoxybenzene, a wide variety of salicylaldehyde derivatives were examined. The substituents on the salicylaldehyde moiety have little influence on the three-component coupling process. To our delight, single substitution bearing halides (Cl and Br) and electron donating groups (–OMe and –OEt) provided the corresponding products in 88 to 94% yields (4b–4e). The reaction was also compatible with 3-methoxy-5-bromosalicylaldehyde and dihalide substitution and provided corresponding chromeno[2,3-*d*]pyrimidines 4f–4i with 81 to 88% yields. Unexpectedly, 4-nitrosalicyldehyde yielded a higher output, suggesting that the electron-withdrawing group at the 4-position of the aldehyde facilitates the reaction. On the other hand, 4-methoxysalicyldehyde did not yield any desired product under our optimized conditions. The reaction gave satisfactory results in the case of 2-hydroxy-1-naphthaldehyde, with 79% yield. We also concentrated our efforts using dibenzylaniline as an electron-rich arene, which provided the 86% yield of the respective chromeno[2,3-*d*]pyrimidine 4l.

**Fig. 4:**
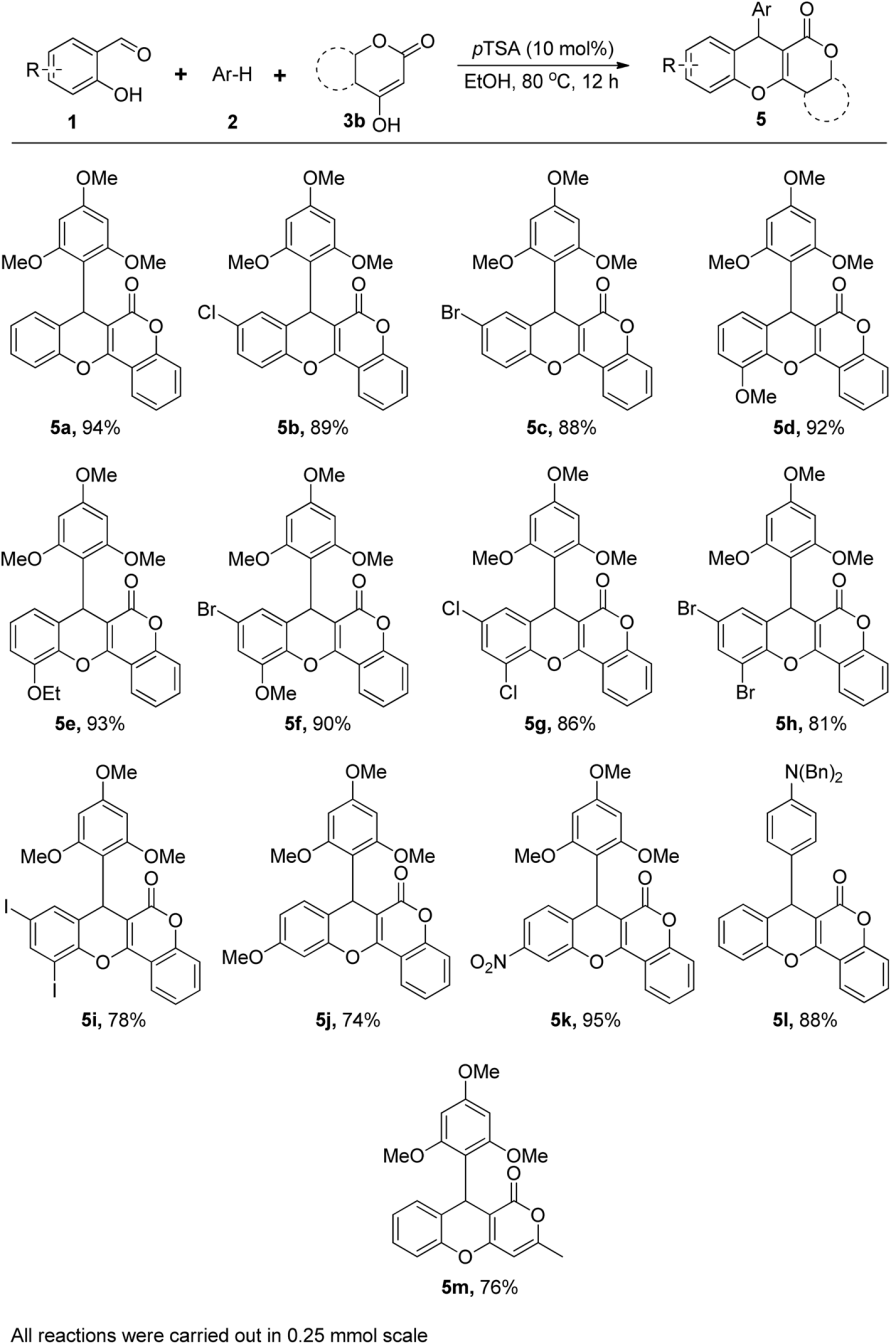
*p*-TSA catalysed synthesis of chromeno[4,3-*b*]chromene: substrate scope.

Subsequently, we applied this three-component coupling reaction using 4-hydroxycoumarin and 1,3,5-trimethoxybenzene with variety of salicylaldehyde derivatives (Fig. 4). Interestingly, under the same optimized condition, the reaction with these entities afforded the expected chromeno[4,3-*b*]chromenes with good to excellent yields. Both single-substituted (Cl, Br –OMe & –OEt) salicylaldehyde and di-substitutedlike 3-methoxy-5-bromosalicylaldehyde and dihalide salicylaldehydes were provided the corresponding chromeno[4,3-*b*]chromenes 5a–5i with satisfactory yield (78 to 94%). The reaction was also compatible with both electron donating –OMe or electron withdrawing –NO_2_ group, attached at the 4-position of aldehyde (5j–5k). However, 4-nitrosalicyldehyde produced a higher yield (95%), indicating that adding an electron-withdrawing group at the 4-position of the aldehyde part made the aldehyde carbonyl more reactive, which led to better results. Additionally, we focused on dibenzylaniline as an electron-rich arene, which yielded the corresponding chromeno[4,3-*b*]chromene 5l with 88% yield. The reaction is compatible only with strong electron-rich arenes. Thus, other electron-rich arenes like phloroglucinol, mesitylene, resorcinol, catechol, phenol, dimethoxybenzene, methoxynaphthalene, aniline and heteroaromatic compounds such as 2-methylthiophene or pyrrole are inactive or very less active in this three-component coupling reaction. Notably, we examined (Fig. 4) this three-component coupling reaction with 4-hydroxy-6-methyl-2-pyrone, salicylaldehyde, and 1,3,5-trimethoxybenzene, but the corresponding product 5m was obtained in moderate yields (76%). The low solubility of the final products resulted in easy separation from the reaction mixture by simple filtration, eliminated extraction steps, and avoided tedious column chromatography, which reduces the use of hazardous organic solvents and chemical impurities. Thus, it was a very positive development from the perspective of green chemistry. Moreover, the products were adequately pure after straight forward filtration, which only needed a solvent wash and recrystallization to make analytically pure form. All final products were properly characterized with the help of various spectral (^1^H, ^13^C NMR, & HRMS) and elemental analysis data.

**Fig. 5:**
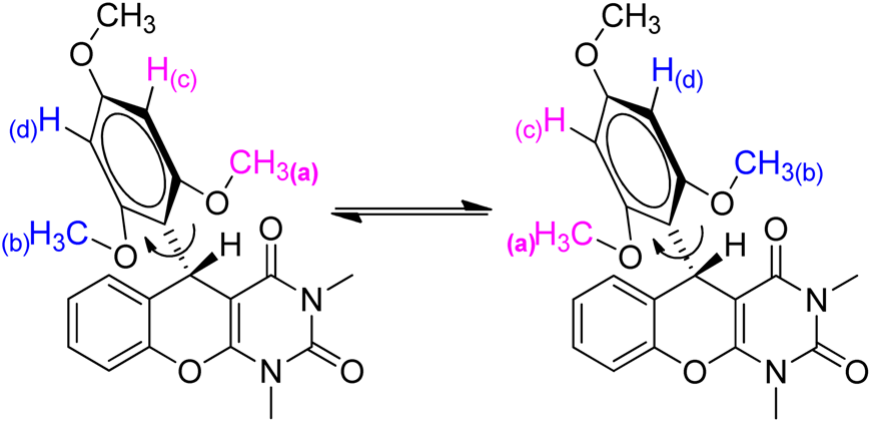
Restricted rotation around the Csp^2^–Csp^3^ bond showing the diastereomeric nature of two *ortho*-methoxy groups (a and b) & two *meta*-protons (c and d).

**Fig. 6:**
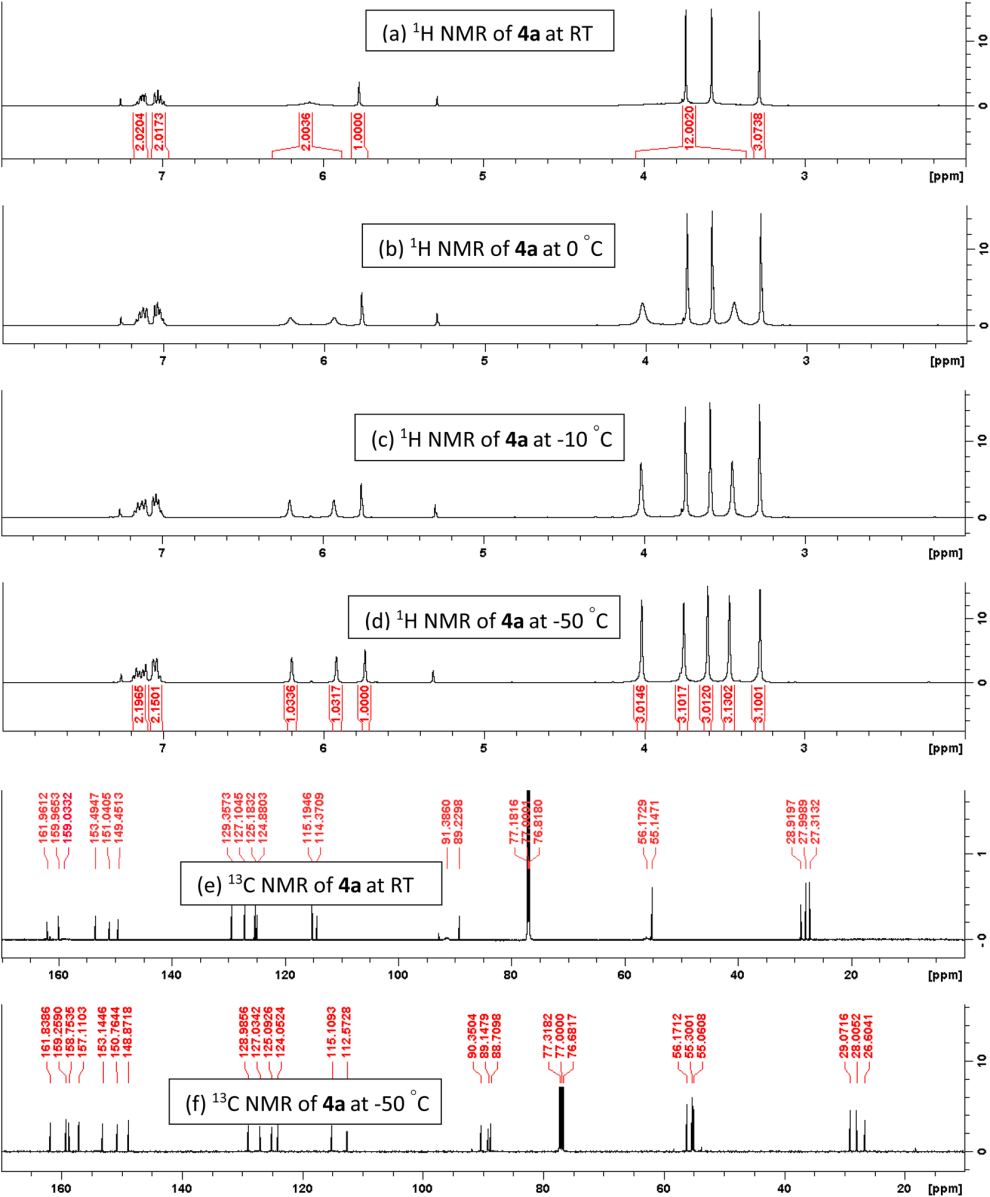
^1^H and ^13^C NMR spectrum of 4a in CDCl_3_ at RT to −50 °C.

**Fig. 7:**
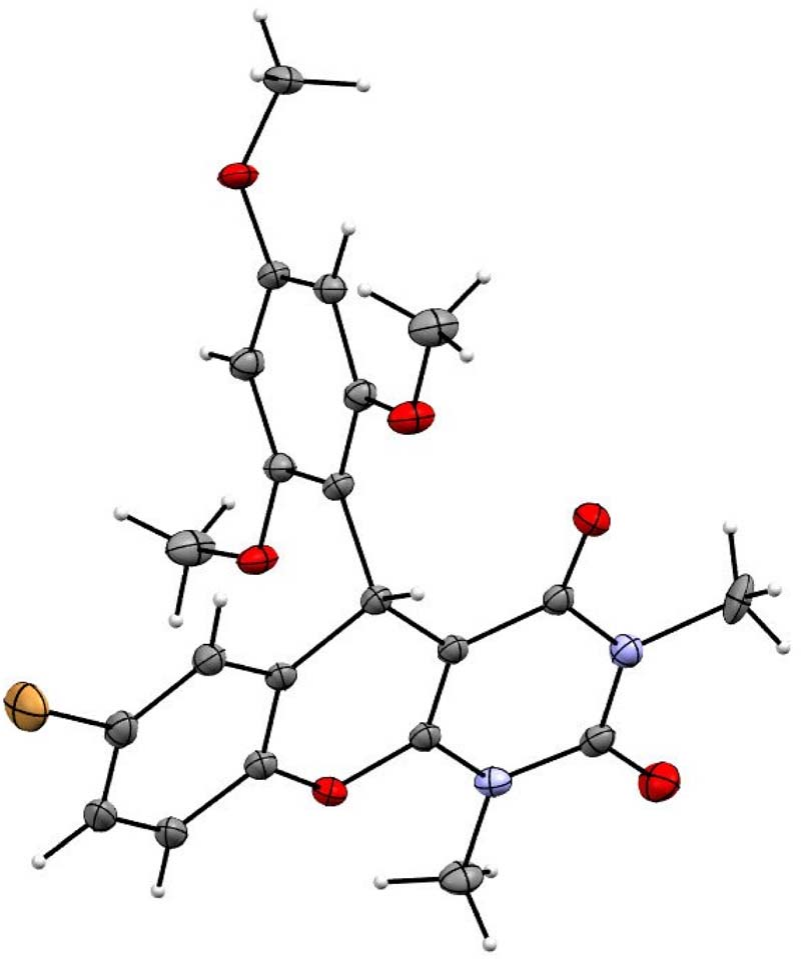
Single crystal X-ray structure of 4c.

In addition, variable temperature (VT) ^1^H &^13^C NMR spectroscopy^13^ was studied to rationalize the dynamic Csp^2^–Csp^3^bond between electron-rich aryl group and benzylic sp^3^-carbon (Fig. 5). If we consider the free rotation around Csp^2^–Csp^3^ bond, the six protons in two *ortho*-methoxy groups (a & b) are chemically and magnetically indistinguishable; we may predict a sharp singlet for all six protons. Similar expectations for two *meta*-protons (c & d) might also be predicted to be a sharp singlet. However, at room temperature, the ^1^H NMR spectrum of 4a in CDCl_3_ (Fig. 6a) shows broad signals for the two *ortho*-methoxy groups (a & b) between 3.38–4.08 ppm and two *meta*-protons (c & d) between 5.85–6.25 ppm (Fig. 6a). Surprisingly, these two broad signals are almost merged with the baseline of the ^1^H NMR spectra and initially confused us to predict the product structure correctly. A similar effect was also observed in the ^13^C NMR spectra at room temperature (Fig. 6e). Two equivalent *ortho*-methoxy carbons and two equivalent *meta*-carbons of electron-rich aryl ring of 4a are showing a very small broad signal at 61 & 91 ppm (Fig. 6e). We also observed that the signal of two equivalent *ortho*-quaternary carbons in ^13^C NMR spectra is almost merged with the baseline around 159 ppm. The broad resonances of ^1^H and ^13^C NMR are due to the restricted rotation around the Csp^2^–Csp^3^ bond. This led to a spectroscopically different of all magnetically equivalent protons and carbons of electron-rich trimethoxy benzene at room temperature since they do not experience the same time-averaged environment. To rationalise the above hypothesis, we have decided to take the ^1^H NMR & ^13^C NMR spectra at low temperature. Upon gradual cooling of the sample, free rotation of trimethoxybenzene moiety (around the Csp^2^–Csp^3^ bond) is frozen, which makes magnetically non-equivalent of two *ortho*- and *meta*-sides. Thus, the signals of two *ortho*-methoxy groups are separated and appeared at 3.42 & 4.0 ppm and two *meta*-protons resonance at 5.86 & 6.02 ppm as a sharp singlet at −50 °C (Fig. 6d). A similar observation was obtained in the ^13^C NMR spectra (Fig. 6f), where all 22 carbons exhibited the sharp signals at −50 °C due to the frozen of restricted rotation about the Csp^2^–Csp^3^ bond. We also noticed a minor chemical shift deviation at freezing temperature in both the ^1^H and ^13^C NMR spectra because the magnetic field varies with magnet temperature. Additionally, single X-ray crystallographic analysis of one selected molecule 4c (CCDC-2441332) (Fig. 7) provided additional confirmation of the entire structural motif.

**Scheme 1:**
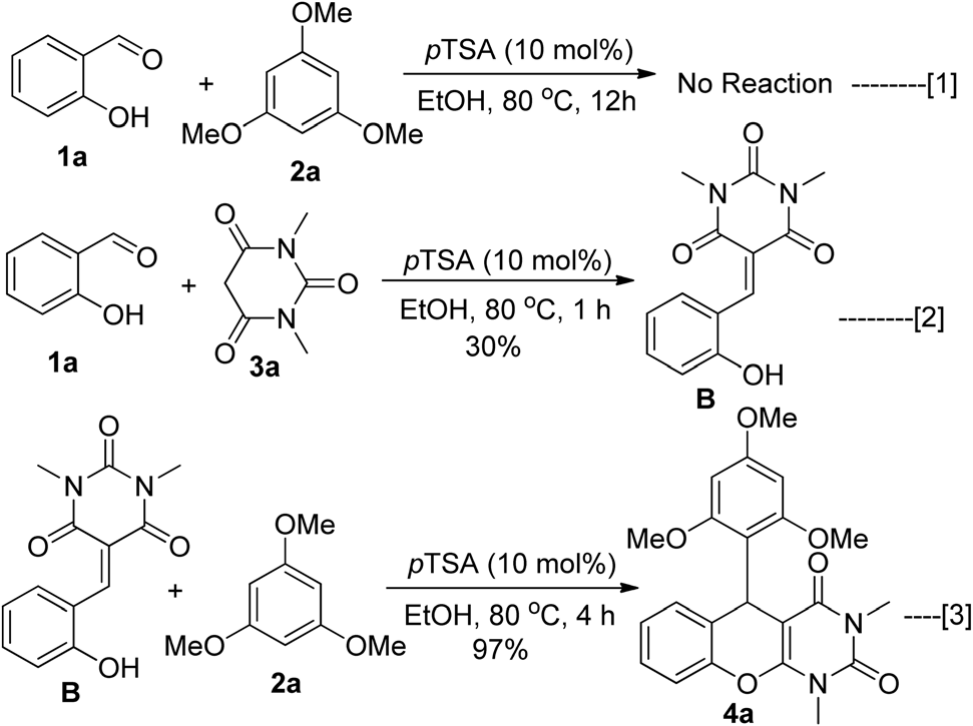
Mechanistic studies for the synthesis of chromeno[2,3-*d*]pyrimidine derivative.

To gain a better understanding of the reaction mechanism, a number of control experiments were conducted and illustrated in Scheme 1. In order to determine the mode of initiation in the *p*TSA/EtOH medium, the control experiment was conducted using 2-hydroxybenzaldehyde 1a and 1,3,5-trimethoxybenzene 2a without 1,3-dimethylbarbituric acid 3a, which did not result in the generation of any adduct (Scheme 1, eqn (1)). The alternative reaction route was further validated by exposing 2-hydroxybenzaldehyde 1a and 1,3-dimethylbarbituric acid 3a to the same ideal conditions, which resulted in the condensed product B in 30% yield in one hour with some unreacted starting materials (Scheme 1, eqn (2)). We separated the intermediate B and characterized it using NMR spectroscopy. We next treated it with 1,3,5-trimethoxybenzene 2a, which yielded the required chromeno[2,3-*d*]pyrimidine 4a with 97% yield under the same ideal conditions (Scheme 1, eqn (3)). This result indicates that our three-component coupling reaction proceeded through the intermediate B.

**Scheme 2:**
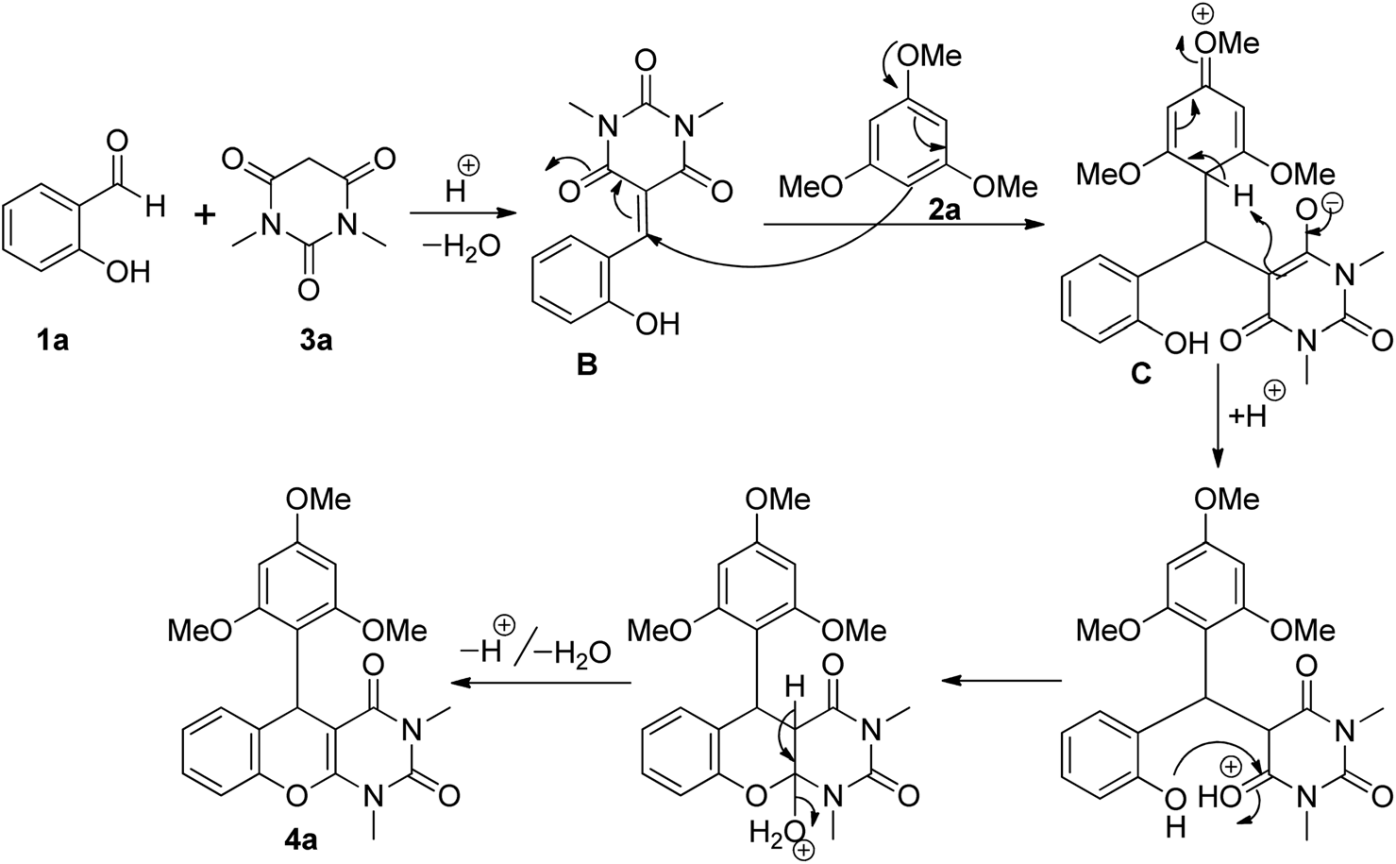
Proposed mechanism for the formation of chromeno[2,3-*d*]pyrimidine derivative 4a.

Based on the above controlled experiments, we assumed that (Scheme 2) the reaction proceeded through the acid catalysed Knoevenagel condensation between salicylaldehyde 1a and 1,3-dimethylbarbituric acid 3a results in the formation of intermediate B. The *p*TSA-promoted Michael type addition of 1,3,5-trimethoxybenzene 2a to the Michael acceptor B resulted in the formation of intermediate C associated with a *tertiary* carbon center. The desired product 4a was then obtained by acid promoted intramolecular ring closure reaction followed by dehydration.

In addition, to investigate the viability of large-scale production, we conducted a scaled-up reaction for the preparation of compound 4a at a 10 mmol scale. We observed that the efficiency and gram-scale yields of this approach are comparable to small-scale system, demonstrating its potential for use in commercial and industrial applications.

## Conclusion

In summary, we successfully explored an economical and environmentally friendly method for the *p*TSA-catalyzed cyclization of salicylaldehyde, 1,3-dimethylbarbituric acid/4-hydroxycoumarin, and electron-rich arenes, which produced the synthetically important chromeno[2,3-*d*]pyrimidine/chromeno[4,3-*b*]chromene derivatives. We conducted controlled experiments to better understand the reaction mechanism by showing that the reaction proceeded through the common intermediate B through a condensation reaction followed by cascade Michael type addition, ring closing reaction and dehydration, producing desired chromeno[2,3-*d*]pyrimidine derivatives. Furthermore, the dynamic Csp^2^–Csp^3^ bond between the electron-rich aryl group and the benzylic sp^3^-carbon is investigated using variable temperature (VT) ^1^H & ^13^C NMR spectroscopy. Additionally, the process provides excellent yields, high functional group tolerance, large-scale synthesis, and column chromatographic free purification. All these features endorse a green development towards the sustainable synthesis of chromeno[2,3-*d*]pyrimidine/chromeno[4,3-*b*]chromene derivatives.

## Conflicts of interest

The authors declare no conflict of interest.

## Supplementary Material

RA-015-D5RA06198A-s001

RA-015-D5RA06198A-s002

## Data Availability

All the data are newly generated and not publish in anywhere. CCDC 2441332 contains the supplementary crystallographic data for this paper.^14^ All analytical data to support our manuscript has been included in the supplementary information (SI). Supplementary information: general information, experimental procedures for catalytic operation, crystal data of 4c, spectral data for all compounds (PDF). ^1^H NMR and ^13^C NMR spectra of all compounds (PDF). See DOI: https://doi.org/10.1039/d5ra06198a.
