# Supplementary material for: pTSA-catalyzed synthesis of functionalized chromeno[2,3-d]pyrimidine/chromeno[4,3-b]chromene derivatives via one-pot three component reaction: mechanistic insights and variable temperature NMR studies to investigate restricted bond rotation
Source: RSC Adv. 2025 Oct 6;15(44):37090–8. doi: 10.1039/d5ra06198a (PMC12499413; doi:10.1039/d5ra06198a)

Supplementary Information (SI) for RSC Advances.
This journal is © The Royal Society of Chemistry 2025

*p*TSA-Catalyzed Synthesis of Functionalized Chromeno[2,3-*d*]pyrimidine/Chromeno[4,3-*b*]chromene Derivatives via One-Pot Three Component Reaction: Mechanistic Insights and Variable Temperature NMR Studies to Investigate Restricted Bond Rotation

Yogesh Bhaskar Singh Tanwer[1], Suman Sourabh[1], Sabyasachi Bhunia*[1], Sanchari Pal[2], Debjit Das*[2]

[1]*Department of Chemistry, Central University of Jharkhand, Ranchi, Jharkhand, India*
E-mail: sabyasachi.bhunia@cuj.ac.in

[2]*Department of Chemistry, Triveni Devi Bhalotia College, Raniganj, India*
E-mail: debjitofchem@gmail.com

Table of Contents:

S-1. General methods

S-2. Experimental procedures

S-3. List of crystallographic parameter details of **4c**.

S-4. Spectral data

S-5. References

S-6. Spectra of products

S-1. General methods

All commercial materials and solvents were used directly without further purification. ^{1}H and ^{13}C NMR were recorded with Bruker AV-400, AV-600, and AV-700 spectrometers and were referenced to residual ^{1}H and ^{13}C signals of the deuterated solvents respectively (δ H 7.26, δ C 77.00 for chloroform) or tetramethylsilane signal. Chemical shifts were reported in δ units (ppm) relative to the TMS signal as an internal reference in $CDCl_3$. Coupling constants, J were reported in Hz. Signal multiplicity was reported as follows: s (singlet), d (doublet), t (triplet), q (quartet), m (multiplet, for complex multiplicity). High-resolution mass spectroscopy were recorded with MALDI-TOF-TOF mass spectrometer. Melting points were obtained using MAC melting point apparatus. Reactions were monitored by thin-layer chromatography (TLC) on silica gel 60 F_{254} plate. Single crystals of **4c** were developed from the slow evaporation of the methanolic solution. A suitable crystal was selected and the crystal data for **4c** has been collected on a Bruker SMART CCD diffractometer (Mo Kα radiation, λ = 0.71073 Å). The crystal was kept at 100 K during data collection. Using Olex2 [1], the structure was solved with the ShelXT [2] structure solution program using Intrinsic

Phasing and refined with the ShelXL [3] refinement package using Least Squares minimisation. All the non-hydrogen atoms were refined with anisotropic displacement parameters. Hydrogens were fixed at calculated positions and refined isotropically.

S-2. Experimental procedures

a. **Preparation of 7-(2,4,6-trimethoxyphenyl)-5,7-dihydro-6*H*-benzo[*c*]xanthen-6-one**

To the mixture of salicylaldehyde (30.53 mg, 0.25 mmol), 1,3,5-trimethoxybenzene (42.04 mg, 0.25 mmol), and 4-Hydroxy Coumarin (40.53 mg, 0.25 mmol) was added *p*-TSA (20 mol %) and EtOH (2.0 mL). The resulting mixture was stirred at 80 ºC for 12 hours, the progress of reaction was monitored by TLC. After completion of the reaction brine solution was added to reaction mixture, and then it was cooled, the solid obtained was filtered, washed with water and chilled EtOH then recrystallized with appropriate solvent to get the pure product.

b. **Preparation of 1,3-dimethyl-5-(2,4,6-trimethoxyphenyl)-1,5-dihydro-2*H*-chromeno[2,3-d]pyrimidine-2,4(3*H*)-dione**

To the mixture of salicylaldehyde (30.53 mg, 0.25 mmol), 1,3,5-trimethoxybenzene (42.04 mg, 0.25 mmol), and 1,3-dimethylbarbituric acid (39.03 mg, 0.25 mmol) was added *p*-TSA (20 mol %) and EtOH (2.0 mL). The resulting mixture was stirred at 80 ºC for 12 hours, the progress of reaction was monitored by TLC. After completion of the reaction brine solution was added to reaction mixture, and then it was cooled, the solid obtained was filtered, washed with water and chilled EtOH then recrystallized with appropriate solvent to get the pure product.

S-3. List of crystallographic parameter details of 4c.

**Table 1: Crystal data and structure refinement for 4c [mo_sv_c_04_0m].**	
Identification code	mo_sv_c_04_0m
Empirical formula	$C_{22}H_{21}BrN_2O_6$
Formula weight	489.32
Temperature/K	100.0
Crystal system	triclinic
Space group	P-1
a/Å	7.8555(2)
b/Å	11.8073(3)
c/Å	12.6358(3)
α/°	68.3900(10)
β/°	73.5550(10)
γ/°	82.2480(10)
Volume/Å^3	1044.43(5)
Z	2
ρ_{calc}g/cm^3	1.556

μ/mm^{-1}	2.011
F(000)	500.0
Crystal size/mm^3	0.24 × 0.22 × 0.19
Radiation	MoKα (λ = 0.71073)
2Θ range for data collection/°	5.41 to 67.324
Index ranges	$-12 \leq h \leq 11$, $-17 \leq k \leq 18$, $-19 \leq l \leq 15$
Reflections collected	33491
Independent reflections	7114 [R_{int} = 0.0519, R_{sigma} = 0.0495]
Data/restraints/parameters	7114/0/285
Goodness-of-fit on F^2	1.037
Final R indexes [I>=2σ (I)]	R_1 = 0.0521, wR_2 = 0.1377
Final R indexes [all data]	R_1 = 0.0781, wR_2 = 0.1569
Largest diff. peak/hole / e Å^{-3}	1.81/-1.85
CCDC Number	2441332

S-4. Spectral data

Due to the restricted rotation around the Csp^2–Csp^3 bond, we observed that in few cases, the signals of two equivalent *ortho*-quaternary carbons and two *ortho*-methoxy methyl carbons of electron rich *tri*-methoxybenzene in ^{13}C NMR spectra are totally merged with the baseline.

Spectral data of 1,3-dimethyl-5-(2,4,6-trimethoxyphenyl)-1,5-dihydro-2*H*-chromeno[2,3-d]pyrimidine-2,4(3*H*)-dione (4a)

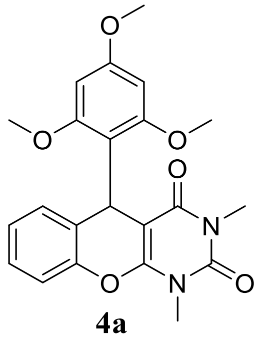

4a

Yield: 95%; White solid, mp 205 °C; R_f = 0.4 (petroleum ether : ethyl acetate = 6 : 4); ^{1}H NMR (400 MHz, $CDCl_3$) δ 3.28 (s, 3H), 3.58 (s, 3H), 3.74 (s, 3H), 3.40-4.06 (bs, 6H), 5.77 (s, 1H), 6.08 (bs, 2H), 6.99-7.04 (m, 2H), 7.10-7.15 (m, 2H). ^{13}C NMR (100 MHz, $CDCl_3$) δ 27.3, 27.9, 28.9, 55.1, 56.1 (br), 89.2, 91.3 (br), 114.3, 115.1, 124.8, 125.1, 127.1, 129.3, 149.4, 151.0, 153.4, 159.0 (br), 159.9, 161.9. Anal. Calcd for $C_{22}H_{22}N_2O_6$: C, 64.38; H, 5.40; N, 6.83. Found: C, 64.62; H, 5.49; N, 6.75.

Spectral data of 7-chloro-1,3-dimethyl-5-(2,4,6-trimethoxyphenyl)-1,5-dihydro-2*H*-chromeno[2,3-d]pyrimidine-2,4(3*H*)-dione (4b)

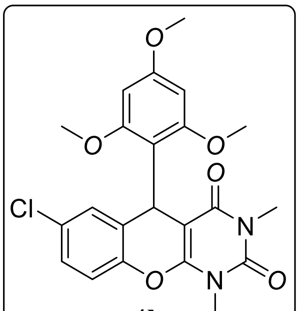

4b

Yield: 88%; White solid, mp 218 ºC; R_{f} = 0.37 (petroleum ether : ethyl acetate = 6 : 4); ^{1}H NMR (400 MHz, $CDCl_3$) δ 3.25(s, 3H), 3.54 (s, 3H), 3.72 (s, 3H), 3.3-4.08 (bs. 6H), 5.70 (s, 1H), 6.05 (bs, 2H), 6.91 (d, 1H, J = 8.6 Hz), 7.18-7.23 (m, 2H). ^{13}C NMR (100 MHz, $CDCl_3$) δ 27.1, 27.8, 28.7, 55.0, 55.8 (br), 88.7, 91.2 (br), 113.4, 116.8, 117.4, 127.0, 130.0, 130.8, 148.4, 150.7, 153.0, 158.5 (br), 160.1, 161.5. HRMS (ESI) m/z: $[M + Na]^{+}$ calcd. for $C_{22}H_{21}ClN_2NaO_6$ $[M + Na]^{+}$ 467.0986; found: 467.0980.

Spectral data of 7-bromo-1,3-dimethyl-5-(2,4,6-trimethoxyphenyl)-1,5-dihydro-2*H*-chromeno[2,3-d]pyrimidine-2,4(3*H*)-dione (4c)

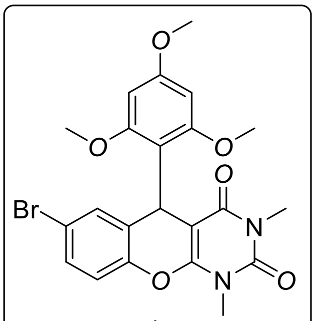

4c

Yield: 89%; White solid, mp 222 ºC; R_{f} = 0.35 (petroleum ether : ethyl acetate = 6 : 4); ^{1}H NMR (400 MHz, $CDCl_3$) δ 3.27 (s, 3H), 3.56 (s, 3H), 3.75 (s, 3H), 3.35-4.1 (bs. 6H), 5.73 (s, 1H), 6.08 (bs, 2H), 6.98 (d, 1H, J = 8.6 Hz), 7.06-7.11 (m, 2H). ^{13}C NMR (100 MHz, $CDCl_3$) δ 27.4, 28.0, 28.9, 55.1, 88.8, 91.3 (br), 113.5, 116.5, 126.7, 127.2, 129.0, 129.9, 148.0, 150.9, 153.2, 160.2, 161.7, (Two peaks are merged with the baseline due to the restricted bond rotation). Anal. Calcd for $C_{22}H_{21}BrN_2O_6$: C, 54.00; H, 4.33; N, 5.73. Found: C, 54.19; H, 4.25; N, 5.81.

Spectral data of 9-methoxy-1,3-dimethyl-5-(2,4,6-trimethoxyphenyl)-1,5-dihydro-2*H*-chromeno[2,3-d]pyrimidine-2,4(3*H*)-dione (4d)

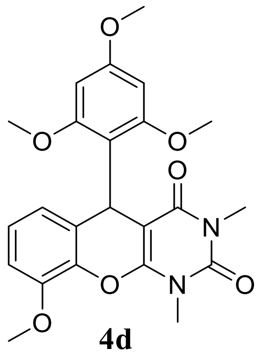

4d

Yield: 94%; White solid, mp 235 ºC; R_{f} = 0.45 (petroleum ether : ethyl acetate = 6 : 4); ^{1}H NMR (400 MHz, $CDCl_3$) δ 3.28 (s, 3H), 3.60 (s, 3H), 3.74 (bs, 3H), 3.4-4.18 (bs. 6H), 3.89 (s, 3H), 5.76 (s, 1H), 6.08 (bs, 2H), 6.69-6.74 (m, 2H), 6.93 (t, 1H, *J* = 7.96 Hz). ^{13}C NMR (100 MHz, $CDCl_3$) δ 27.4, 27.9, 28.9, 55.1, 56.0, 88.9, 91.4 (br), 109.6, 114.2, 120.6, 124.6, 125.7, 139.1, 147.1, 151.0, 153.4, 159.4 (br), 159.9, 161.9, (One peak is merged with the baseline due to the restricted bond rotation). HRMS (ESI) m/z: $[M + Na]^+$ calcd. for $C_{23}H_{24}N_2NaO_7$ $[M + Na]^+$ 463.1481; found: 463.1476.

Spectral data of 9-ethoxy-1,3-dimethyl-5-(2,4,6-trimethoxyphenyl)-1,5-dihydro-2*H*-chromeno[2,3-d]pyrimidine-2,4(3*H*)-dione (4e)

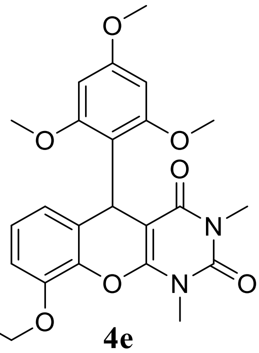

4e

Yield: 92%; White solid, mp 207 ºC; R_{f} = 0.45 (petroleum ether : ethyl acetate = 6 : 4); ^{1}H NMR (700 MHz, $CDCl_3$) δ 1.46 (t, 3H, *J* = 6.93 Hz). 3.28 (s, 3H), 3.60 (s, 3H), 3.74 (s, 3H), 3.45-4.08 (bs. 6H), 4.04-4.14 (m, 2H), 5.76 (s, 1H), 6.08 (bs, 2H), 6.68 (d, 1H. *J* = 7.7 Hz), 6.72 (d, 1H, *J* = 8.05 Hz), 6.90 (t, 1H, *J* = 7.91 Hz). ^{13}C NMR (175 MHz, $CDCl_3$) δ 14.9, 27.4, 27.9, 28.8, 55.1, 56.1 (br), 64.6, 88.9, 91.4 (br), 111.2, 114.2, 120.6, 124.6, 125.7, 139.4, 146.4, 151.1, 153.4, 159.2 (br), 159.9, 162.0. Anal. Calcd for $C_{24}H_{26}N_2O_7$: C, 63.43; H, 5.77; N, 6.16. Found: C, 63.21; H, 5.72; N, 6.09.

Spectral data of 7-bromo-9-methoxy-1,3-dimethyl-5-(2,4,6-trimethoxyphenyl)-1,5-dihydro-2*H*-chromeno[2,3-d]pyrimidine-2,4(3*H*)-dione (4f)

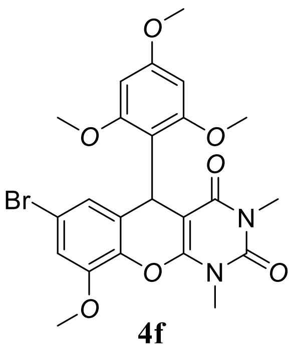

4f

Yield: 88%; White solid, mp 244 ºC; R_f = 0.36 (petroleum ether : ethyl acetate = 6 : 4); ^{1}H NMR (400 MHz, $CDCl_3$) δ 3.27 (s, 3H), 3.58 (s, 3H), 3.75 (s, 3H), 3.88 (s, 3H), 3.5-4.09 (bs. 6H), 5.71 (s, 1H), 6.08 (bs, 2H), 6.81 (s, 1H), 6.84 (s, 1H). ^{13}C NMR (100 MHz, $CDCl_3$) δ 27.4, 27.9, 28.9, 55.1, 56.0 (br), 56.2, 88.7, 91.3 (br), 113.2, 113.3, 116.9, 123.2, 127.3, 138.4, 147.8, 150.9, 153.2, 160.2, 161.7, (One peak is merged with the baseline due to the restricted bond rotation). Anal. Calcd for $C_{23}H_{23}BrN_2O_7$: C, 53.19; H, 4.46; N, 5.39. Found: C, 53.29; H, 4.41; N, 5.42.

Spectral data of 7,9-dichloro-1,3-dimethyl-5-(2,4,6-trimethoxyphenyl)-1,5-dihydro-2*H*-chromeno[2,3-d]pyrimidine-2,4(3*H*)-dione (4g)

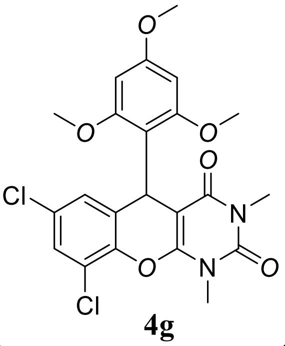

4g

Yield: 85%; White solid, mp 230 ºC; R_f = 0.4 (petroleum ether : ethyl acetate = 5 : 5); ^{1}H NMR (400 MHz, $CDCl_3$) δ 3.26 (s, 3H), 3.60 (s, 3H), 3.73 (s, 3H), 3.51-4.08 (bs. 6H), 5.72 (s, 1H), 6.05 (bs, 2H), 6.96 (d, 1H, J = 2.28 Hz), 7.19 (d, 1H, J = 2.4 Hz). ^{13}C NMR (100 MHz, $CDCl_3$) δ 27.6, 27.9, 28.9, 55.0, 55.8 (br), 88.6, 91.2, 112.9, 121.4, 127.3, 127.5, 127.9, 129.6, 144.2, 150.6, 152.7, 158.5 (br), 160.3, 161.4. HRMS (ESI) m/z: $[M + Na]^+$ calcd. for $C_{22}H_{20}Cl_2N_2NaO_6$ $[M + Na]^+$ 501.0596; found: 501.0591.

Spectral data of 7,9-dibromo-1,3-dimethyl-5-(2,4,6-trimethoxyphenyl)-1,5-dihydro-2*H*-chromeno[2,3-d]pyrimidine-2,4(3*H*)-dione (4h)

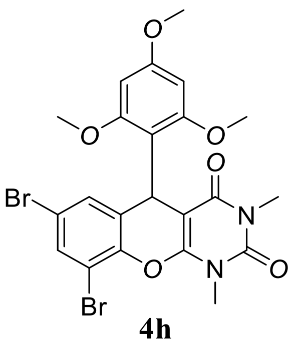

4h

Yield: 84%; White solid, mp 250 ºC; R_f = 0.37 (petroleum ether : ethyl acetate = 5 : 5); ^{1}H NMR (400 MHz, $CDCl_3$) δ 3.27 (s, 3H), 3.62 (s, 3H), 3.75 (s, 3H), 3.45-4.18 (bs. 6H), 5.75 (s, 1H), 6.08 (bs, 2H), 7.15 (d, 1H, *J* = 1.8 Hz), 7.51 (d, 1H, *J* = 2.08 Hz). ^{13}C NMR (100 MHz, $CDCl_3$) δ 27.8, 28.0, 29.2, 55.2, 56.0 (br), 88.9, 91.2, 110.2, 113.0, 117.4, 128.4, 131.1, 133.1, 145.8, 150.7, 153.0, 158.5 (br), 160.4, 161.5. Anal. Calcd for $C_{22}H_{20}Br_2N_2O_6$: C, 46.50; H, 3.55; N, 4.93. Found: C, 46.29; H, 3.60; N, 4.98.

Spectral data of 7,9-diiodo-1,3-dimethyl-5-(2,4,6-trimethoxyphenyl)-1,5-dihydro-2*H*-chromeno[2,3-d]pyrimidine-2,4(3*H*)-dione (4i)

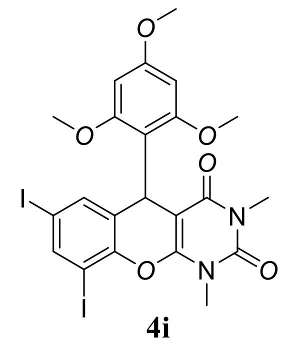

4i

Yield: 81%; White solid, mp 250 ºC; R_f = 0.36 (petroleum ether : ethyl acetate = 5 : 5); ^{1}H NMR (400 MHz, $CDCl_3$) δ 3.27 (s, 3H), 3.66 (s, 3H), 3.76 (s, 3H), 3.47-4.28 (bs. 6H), 5.72 (s, 1H), 6.08 (bs, 2H), 7.33 (d, 1H, *J* = 1.08 Hz), 7.88 (d, 1H, *J* = 1.64 Hz). ^{13}C NMR (100 MHz, $CDCl_3$) δ 27.8, 28.0, 29.7, 55.2, 55.9 (br), 83.8, 88.7, 89.1, 91.3, 113.2, 128.1, 138.0, 144.3, 149.1, 150.7, 153.3, 160.4, 161.4, (One peak is merged with the baseline due to the restricted bond rotation). Anal. Calcd for $C_{22}H_{20}I_2N_2O_6$: C, 39.90; H, 3.04; N, 4.23. Found: C, 40.17; H, 3.11; N, 4.12.

Spectral data of 1,3-dimethyl-8-nitro-5-(2,4,6-trimethoxyphenyl)-1,5-dihydro-2*H*-chromeno[2,3-d]pyrimidine-2,4(3*H*)-dione (4j)

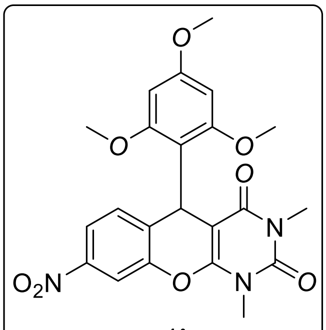

4j

Yield: 96%; White solid, mp 243 ºC; R_{f} = 0.42 (petroleum ether : ethyl acetate = 6 : 4); ^{1}H NMR (400 MHz, $CDCl_3$) δ 3.28 (s, 3H), 3.59 (s, 3H), 3.74 (s, 3H), 3.3-4.24 (bs. 6H), 5.83 (s, 1H), 6.08 (bs, 2H), 7.27 (d, 1H, *J* = 9.2 Hz), 7.87 (d, 1H, *J* = 8.4 Hz), 7.94 (s, 1H). ^{13}C NMR (100 MHz, $CDCl_3$) δ 27.6, 28.0, 29.0, 55.2, 56.0 (br), 88.6, 91.2, 111.0, 112.9, 120.0, 130.2, 132.7, 146.7, 149.4, 150.7, 152.9, 158.5 (br), 160.6, 161.5. Anal. Calcd for $C_{22}H_{21}N_3O_8$: C, 58.02; H, 4.65; N, 9.23. Found: C, 58.31; H, 4.59; N, 9.28.

Spectral data of 8,10-dimethyl-12-(2,4,6-trimethoxyphenyl)-8,12-dihydro-9*H*-benzo[5,6]chromeno[2,3-d]pyrimidine-9,11(10*H*)-dione (4k)

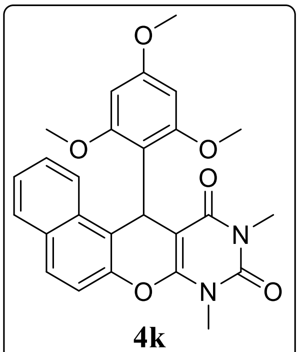

4k

Yield: 79%; White solid, mp 205 ºC; R_{f} = 0.51 (petroleum ether : ethyl acetate = 6 : 4); ^{1}H NMR (400 MHz, $CDCl_3$) δ 3.30 (s, 3H), 3.60 (bs, 6H), 3.68 (bs, 6H), 6.07 (bs, 2H), 6.24 (s, 1H), 7.24 (d, 1H, *J* = 3.48 Hz), 7.33-7.44 (m, 2H), 7.69 (d, 1H, *J* = 8.88 Hz), 7.73 (d, 1H, *J* = 7.6 Hz), 8.31 (d, 1H, *J* = 8.32 Hz). ^{13}C NMR (100 MHz, $CDCl_3$) δ 25.7, 28.0, 28.8, 55.0, 89.4, 91.5 (br), 112.8, 115.7, 117.4, 124.2, 124.8, 126.8, 128.1, 128.3, 131.3, 131.8, 147.7, 150.9, 153.3, 160.0, 161.9, (Two peaks are merged with the baseline due to the restricted bond rotation). HRMS (ESI) m/z: $[M + Na]^+$ calcd. for $C_{26}H_{24}N_2NaO_6$ $[M + Na]^+$ 483.1532; found: 483.1527.

Spectral data of 5-(4-(dibenzylamino)phenyl)-1,3-dimethyl-1,5-dihydro-2*H*-chromeno[2,3-d]pyrimidine-2,4(3*H*)-dione (4l)

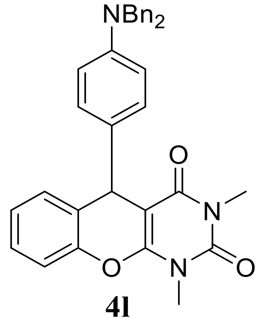

4l

Yield: 86%; White solid, mp 215 ºC; $R_{f=}$ 0.56 (petroleum ether : ethyl acetate = 6 : 4); ^{1}H NMR (400 MHz, $CDCl_3$) δ 3.31 (s, 3H), 3.55 (s, 3H), 4.57 (s, 4H), 5.06 (s, 1H), 6.60 (d, 2H, *J* = 8.72 Hz), 7.02 (d, 2H, *J* = 8.72 Hz), 7.10-7.15 (m, 2H), 7.18-7.24 (m, 8H), 7.27-7.31 (m, 4H); ^{13}C NMR (100 MHz, $CDCl_3$) δ 28.1, 28.9, 37.7, 54.2, 91.1, 112.2, 116.2, 125.3, 125.9, 126.6, 126.8, 127.6, 128.50, 128.55, 130.1, 133.4, 138.6, 147.9, 148.9, 150.7, 152.4, 162.0. Anal. Calcd for $C_{33}H_{29}N_3O_3$: C, 76.87; H, 5.67; N, 8.15. Found: C, 76.60; H, 5.61; N, 8.34.

Spectral data of 7-(2,4,6-trimethoxyphenyl)-5,7-dihydro-6*H*-benzo[c]xanthen-6-one (5a)

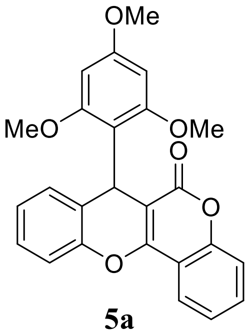

5a

Yield: 94%; White solid, mp 226 ºC; $R_{f=}$ 0.62 (petroleum ether : ethyl acetate = 8 : 2); ^{1}H NMR (700 MHz, $CDCl_3$) δ 3.74 (s, 3H), 3.33-4.19 (bs, 6H), 5.83 (s, 1H), 6.08 (bs, 2H), 7.02 (t, 1H, *J* = 7.49 Hz), 7.13-7.18 (m, 3H), 7.31 (d, 1H, *J* = 8.19 Hz), 7.34 (t, 1H, *J* = 7.49 Hz), 7.52 (t, 1H, *J* = 7.35 Hz), 8.04 (d, 1H, *J* = 7.56 Hz). ^{13}C NMR (175 MHz, $CDCl_3$) δ 27.8, 55.1, 56.1 (br), 91.4, 103.0, 114.2, 114.7, 115.5, 116.6, 122.4, 123.7, 124.4, 124.8, 127.2, 129.3, 131.2, 149.6, 152.5, 156.3, 160.1, 161.4, (One peak is merged with the baseline due to the restricted bond rotation). HRMS (ESI) m/z: $[M + Na]^+$ calcd. for $C_{25}H_{20}O_6Na$ $[M + Na]^+$ 439.1158; found: 439.1136.

Spectral data of 9-chloro-7-(2,4,6-trimethoxyphenyl)-5,7-dihydro-6*H*-benzo[c]xanthen-6-one (5b)

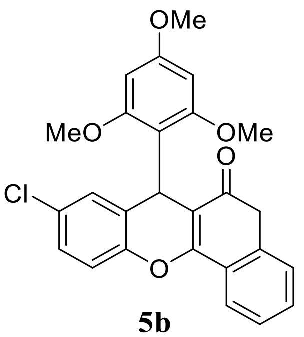

5b

Yield: 89%; White solid, mp 225 ºC; R_f = 0.59 (petroleum ether : ethyl acetate = 8 : 2); ^{1}H NMR (600 MHz, $CDCl_3$) δ 3.75 (s, 3H), 3.41-4.11 (bs, 6H), 5.79 (s, 1H), 6.08 (bs, 2H), 7.09-7.14 (m, 3H), 7.31 (d, 1H, *J* = 8.22 Hz), 7.34 (t, 1H, *J* = 7.5 Hz), 7.53 (t, 1H, *J* = 7.74 Hz), 8.00 (d, 1H, *J* = 7.86 Hz). ^{13}C NMR (150 MHz, $CDCl_3$) δ 27.9, 55.2, 56.0 (br), 91.4, 102.7, 113.4, 114.5, 116.7, 116.9, 122.3, 123.9, 126.3, 127.4, 129, 129.5, 131.5, 148.3, 152.5, 156.1, 158.8 (br), 160.4, 161.1. Anal. Calcd for $C_{25}H_{19}ClO_6$: C, 66.60; H, 4.25. Found: C, 66.39; H, 4.31.

Spectral data of 9-bromo-7-(2,4,6-trimethoxyphenyl)-6*H*,7*H*-chromeno[4,3-b]chromen-6-one (5c)

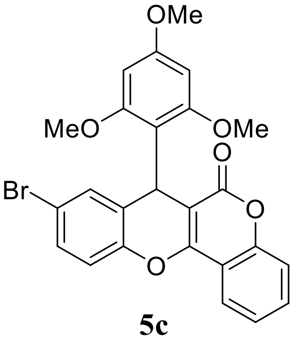

5c

Yield: 88%; White solid, mp 225 ºC; R_f = 0.55 (petroleum ether : ethyl acetate = 8 : 2); ^{1}H NMR (400 MHz, $CDCl_3$) δ 3.75 (s, 3H), 3.43-.4.34 (bs, 6H), 5.79 (s, 1H), 6.08 (bs, 2H), 7.04 (d, 1H, *J* = 8.8 Hz), 7.23 (d, 1H, *J* = 1.76 Hz), 7.26-7.36 (m, 3H), 7.53 (t, 1H, *J* = 7.1 Hz), 8.00 (d, 1H, *J* = 7.1 Hz). ^{13}C NMR (100 MHz, $CDCl_3$) δ 27.8, 55.2, 56.0 (br), 91.4, 102.7, 113.3, 114.4, 116.6, 117.1, 117.3, 122.3, 123.8, 126.7, 130.3, 131.4, 132.0, 148.8, 152.5, 156, 158.9 (br), 160.4, 161.1. HRMS (ESI) m/z: $[M + Na]^+$ calcd. for $C_{25}H_{19}BrNaO_6$ $[M + Na]^+$ 517.0263; found 517.0257.

Spectral data of 11-methoxy-7-(2,4,6-trimethoxyphenyl)-5,7-dihydro-6*H*-benzo[c]xanthen-6-one (5d)

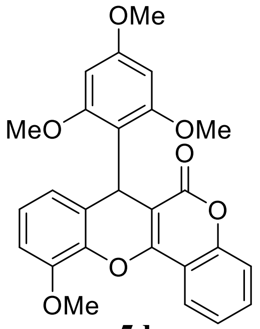

5d

Yield: 92%; White solid, mp 220 ºC; R_{f} = 0.52 (petroleum ether : ethyl acetate = 8 : 2); ^{1}H NMR (600 MHz, $CDCl_3$) δ 3.74 (s, 3H), 3.50-3.94 (bs, 6H), 3.96 (s, 3H), 5.82 (s, 1H), 6.08 (bs, 2H), 6.73 (d, 1H, *J* = 7.56 Hz), 6.77 (d, 1H, *J* = 7.92 Hz), 6.94 (t, 1H, *J* = 7.68 Hz), 7.30 (d, 1H, *J* = 8.1 Hz), 7.34 (t, 1H, *J* = 7.38 Hz), 7.52 (t, 1H, *J* = 7.62 Hz), 8.12 (d, 1H, *J* = 7.68 Hz). ^{13}C NMR (150 MHz, $CDCl_3$) δ 27.8, 55.1, 56.2, 91.5, 102.8, 110.1, 114.1, 114.9, 116.4, 120.9, 122.8, 123.7, 124.3, 125.3, 131.2, 139.5, 147.4, 152.5, 156.2, 159.0 (br), 160.1, 161.4, (One peak is merged with the baseline due to the restricted bond rotation). HRMS (ESI) m/z: $[M + Na]^+$ calcd. for $C_{26}H_{22}NaO_7$ $[M + Na]^+$ 469.1263; found 469.1240.

Spectral data of 11-ethoxy-7-(2,4,6-trimethoxyphenyl)-5,7-dihydro-6*H*-benzo[c]xanthen-6-one (5e)

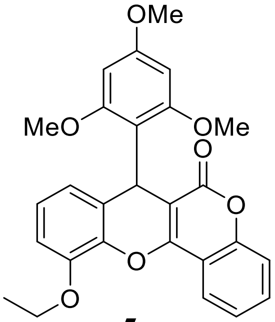

5e

Yield: 93%; White solid, mp 236 ºC; R_{f} = 0.49 (petroleum ether : ethyl acetate = 8 : 2); ^{1}H NMR (600 MHz, $CDCl_3$) δ 1.54 (t, 3H, *J* = 6.66 Hz), 3.74 (s, 3H), 3.57-3.89 (bs, 6H), 4.13-4.20 (m, 2H), 5.82 (s, 1H), 6.08 (bs, 2H), 6.73 (d, 1H, *J* = 7.56 Hz), 6.77 (d, 1H, *J* = 7.8 Hz), 6.91 (t, 1H, *J* = 7.5 Hz), 7.30 (d, 1H, *J* = 8.1 Hz), 7.35 (t, 1H, *J* = 7.38 Hz), 7.52 (t, 1H, *J* = 7.5 Hz), 8.09 (d, 1H, *J* = 7.68 Hz). ^{13}C NMR (150 MHz, $CDCl_3$) δ 15, 27.9, 55.1, 56.1 (br), 65.0, 91.5, 102.7, 112, 114.1, 115, 116.4, 121, 122.7, 123.8, 124.3, 125.4, 131.2, 139.9, 146.7, 152.5, 156.2, 158.9 (br), 160.1, 161.4. HRMS (ESI) m/z: $[M + Na]^+$ calcd. for $C_{27}H_{24}NaO_7$ $[M + Na]^+$ 483.1420; found 483.1399.

Spectral data of 9-bromo-11-methoxy-7-(2,4,6-trimethoxyphenyl)-6*H*,7*H*-chromeno[4,3-b]chromen-6-one (5f)

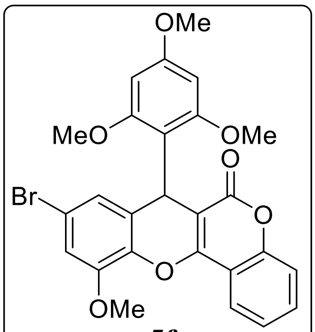

5f

Yield: 90%; White solid, mp 240 ºC; R_{f} = 0.49 (petroleum ether : ethyl acetate = 8 : 2); ^{1}H NMR (400 MHz, $CDCl_3$) δ 3.75 (s, 3H), 3.31-3.89 (bs, 6H), 3.94 (s, 3H), 5.76 (s, 1H), 6.08 (bs, 2H), 6.84 (s, 1H), 6.88 (s, 1H), 7.30 (d, 1H, *J* = 8.2 Hz), 7.34 (t, 1H, *J* = 7.38 Hz), 7.52 (t, 1H, *J* = 7.64 Hz), 8.08 (d, 1H, *J* = 7.8 Hz). ^{13}C NMR (100 MHz, $CDCl_3$) δ 27.8, 55.2, 56.1 (br), 56.4, 91.4, 102.6 113.2, 113.5, 114.6, 116.5 (two peaks merged), 122.7, 123.4, 123.8, 126.9, 131.4, 138.7, 148.2, 152.5, 155.9, 158.9 (br), 160.4, 161.1. Anal. Calcd for $C_{26}H_{21}BrO_7$: C, 59.44; H, 4.03. Found: C, 59.62; H, 4.08.

Spectral data of 9,11-dichloro-7-(2,4,6-trimethoxyphenyl)-6*H*,7*H*-chromeno[4,3-b]chromen-6-one (5g)

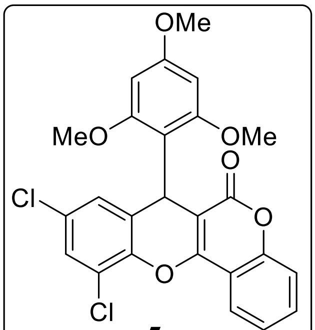

5g

Yield: 86%; White solid, mp 158 ºC; R_{f} = 0.6 (petroleum ether : ethyl acetate = 8 : 2); ^{1}H NMR (400 MHz, $CDCl_3$) δ 3.75 (s, 3H), 3.38-4.13 (bs, 6H), 5.79 (s, 1H), 6.08 (bs, 2H), 7.01 (d, 1H, *J* = 2.0 Hz), 7.24 (d, 1H, *J* = 2.4 Hz), 7.31 (d, 1H, *J* = 8.0 Hz), 7.37 (t, 1H, *J* = 7.6 Hz), 7.55 (t, 1H, *J* = 7.2 Hz), 8.09 (dd, 1H, *J* = 7.84 Hz, *J* = 1.0 Hz). ^{13}C NMR (100 MHz, $CDCl_3$) δ 28.1, 55.2, 56.0 (br), 91.3, 102.7, 112.8, 114.3, 116.6, 121.9, 122.6, 124.1, 127.5, 127.5, 127.8, 129.2, 131.7, 144.5, 152.5, 155.7, 158.8 (br), 160.6, 160.8. Anal. Calcd for $C_{25}H_{18}Cl_2O_6$: C, 61.87; H, 3.74. Found: C, 61.61; H, 3.68.

Spectral data of 9,11-dibromo-7-(2,4,6-trimethoxyphenyl)-6*H*,7*H*-chromeno[4,3-b]chromen-6-one (5h)

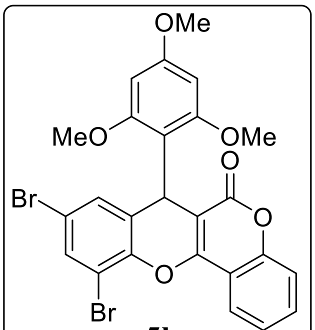

5h

Yield: 81%; White solid, mp 240 ºC; $R_f = 0.58$ (petroleum ether : ethyl acetate = 8 : 2); ^{1}H NMR (400 MHz, $CDCl_3$) δ 3.75 (s, 3H), 3.47-3.98 (bs, 6H), 5.79 (s, 1H), 6.08 (bs, 2H), 7.18 (s, 1H), 7.31 (d, 1H, *J* = 8.24 Hz), 7.37 (t, 1H, *J* = 7.48 Hz), 7.55 (d, 2H, *J* = 10.4 Hz), 8.11 (d, 1H, *J* = 7.68 Hz). ^{13}C NMR (100 MHz, $CDCl_3$) δ 28.2, 55.2, 56.0 (br), 91.3, 102.8, 110.7, 112.9, 114.2, 116.6, 116.9, 122.7, 124.1, 127.9, 131.1, 131.7, 133.3, 145.9, 152.5, 155.9, 158.8 (br), 160.6, 160.8. Anal. Calcd for $C_{25}H_{18}Br_2O_6$: C, 52.29; H, 3.16. Found: C, 52.43; H, 3.21.

Spectral data of 9,11-diiodo-7-(2,4,6-trimethoxyphenyl)-6*H*,7*H*-chromeno[4,3-b]chromen-6-one (5i)

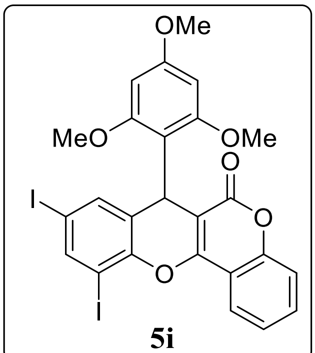

5i

Yield: 78%; White solid, mp 230 ºC; $R_f = 0.57$ (petroleum ether : ethyl acetate = 8 : 2); ^{1}H NMR (400 MHz, $CDCl_3$) δ 3.75 (s, 3H), 3.51-3.96 (bs, 6H) 5.76 (s, 1H), 6.07 (bs, 2H), 7.31 (d, 1H, *J* = 8.24 Hz), 7.35-7.40 (m, 2H), 7.55 (td, 1H, *J* = 7.86 Hz, 1.48 Hz), 7.92 (d, 1H, *J* = 1.84 Hz), 8.16 (dd, 1H, *J* = 7.88 Hz, 1.32 Hz). ^{13}C NMR (100 MHz, $CDCl_3$) δ 28.1, 55.2, 56.0 (br), 84.6, 88.2, 91.3, 103.1, 113.0, 114.2, 116.6, 122.9, 124.1, 127.6, 131.7, 138.1, 144.4, 149, 152.5, 156.2, 160.6, 160.7, (One peak is merged with the baseline due to the

restricted bond rotation). Anal. Calcd for $C_{25}H_{18}I_2O_6$: C, 44.94; H, 2.72. Found: C, 44.82; H, 2.79.

Spectral data of 10-methoxy-7-(2,4,6-trimethoxyphenyl)-6*H*,7*H*-chromeno[4,3-b]chromen-6-one (5j)

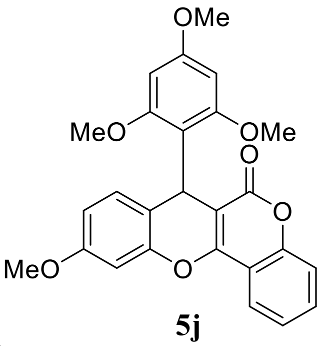

5j

Yield: 74%; White solid, mp 176 ºC; $R_{f\,=}$ 0.52 (petroleum ether : ethyl acetate = 8 : 2); ^{1}H NMR (500 MHz, $CDCl_3$) δ 3.73 (bs, 6H), 3.80 (bs, 6H), 5.76 (s, 1H), 6.08 (s, 2H), 6.60 (dd, 1H, *J* = 2.5 Hz), 6.71 (d, 1H, *J* = 2.5 Hz), 7.03 (d, 1H, *J* = 8.5 Hz), 7.30 (d, 1H, *J* = 8.5 Hz), 7.33 (t, 1H, *J* = 7.5 Hz), 7.51 (t, 1H, 7.0 Hz), 8.03 (d, 1H, *J* = 7.9 Hz, *J* = 1.45 Hz). ^{13}C NMR (125 MHz, $CDCl_3$) δ 27.3, 55.1, 55.3, 56.1 (br), 91.5, 100.8, 103.4, 111.1, 114.3, 114.7, 116.54, 116.56, 122.3, 123.7, 129.7, 131.2, 150.1, 152.5, 156.2, 158.7, 160.0, 161.3, (One peak is merged with the baseline due to the restricted bond rotation). Anal. Calcd for $C_{26}H_{22}O_7$: C, 69.95; H, 4.97. Found: C, 69.82; H, 4.91.

Spectral data of 10-nitro-7-(2,4,6-trimethoxyphenyl)-6*H*,7*H*-chromeno[4,3-b]chromen-6-one (5k)

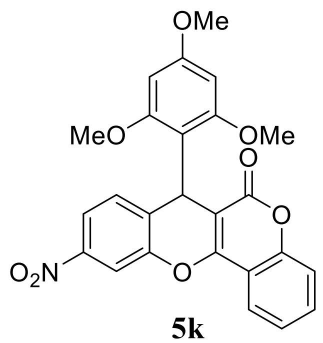

5k

Yield: 95%; White solid, mp 228 ºC; $R_{f\,=}$ 0.49 (petroleum ether : ethyl acetate = 8 : 2); ^{1}H NMR (400 MHz, $CDCl_3$) δ 3.75 (s, 3H), 3.44-4.07 (bs, 6H), 5.89 (s, 1H), 6.08 (s, 2H), 7.29 (d, 1H, *J* = 8.4 Hz), 7.33 (d, 1H, *J* = 8.4 Hz), 7.38 (t, 1H, *J* = 7.6 Hz), 7.57 (t, 1H, *J* = 7.6 Hz), 7.87 (d, 1H, *J* = 8.4 Hz), 8.02-8.05 (m, 2H). ^{13}C NMR (100 MHz, $CDCl_3$) δ 28.1, 55.2, 56.0

(br), 91.3, 102.6, 111.2, 112.7, 114.0, 116.7, 119.6, 122.2, 124.1, 130.2, 131.8, 132.2, 146.9, 149.7, 152.5, 155.7, 158.8 (br), 160.7, 160.8. Anal. Calcd for $C_{25}H_{19}NO_8$: C, 65.07; H, 4.15; N, 3.04. Found: C, 64.88; H, 4.23; N, 3.09.

Spectral data of 7-(4-(dibenzylamino)phenyl)-6*H*,7*H*-chromeno[4,3-b]chromen-6-one (5l)

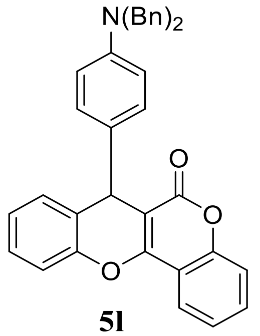

5l

Yield: 88%; White solid, mp 223 °C; R_f = 0.63 (petroleum ether : ethyl acetate = 8 : 2); ^{1}H NMR (400 MHz, $CDCl_3$) δ 4.55 (s, 4H), 5.10 (s, 1H), 6.57 (d, 2H, J = 8.6 Hz), 7.05 (d, 2H, J = 8.6 Hz), 7.09-7.21 (m, 8H), 7.24-7.35 (m, 8H), 7.52 (t, 1H, J = 7.24 Hz), 8.01 (d, 1H, J = 7.72 Hz). ^{13}C NMR (100 MHz, $CDCl_3$) δ 38.2, 54.2, 105.0, 112.3, 114.6, 116.62, 116.66, 122.6, 124.0, 124.8, 125.6, 126.5, 126.8, 127.8, 128.5, 128.8, 130.1, 131.7, 132.9, 138.5, 148.0, 149.0, 152.5, 155.2, 161.5. Anal. Calcd for $C_{36}H_{27}NO_3$: C, 82.90; H, 5.22; N, 2.69. Found: C, 82.69; H, 5.12; N, 2.73.

Spectral data of 3-methyl-10-(2,4,6-trimethoxyphenyl)-1*H*,10*H*-pyrano[4,3-b]chromen-1-one (6)

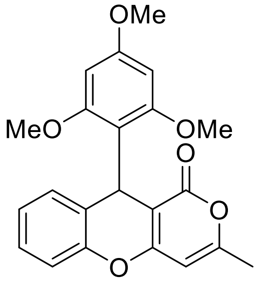

6

Yield: 76%; White solid, mp 201 °C; R_f = 0.56 (petroleum ether : ethyl acetate = 8 : 2); ^{1}H NMR (400 MHz, $CDCl_3$) δ 2.20 (s, 3H), 3.74 (s, 3H), 3.44-4.02 (m, 6H), 5.67 (s, 1H), 5.93 (s, 1H), 6.08 (bs, 2H), 6.93-6.98 (m, 2H), 7.06 - 7.12 (m, 2H). ^{13}C NMR (100 MHz, $CDCl_3$) δ 19.8, 27.1, 55.1, 56.1 (br), 91.5, 98.9, 100.2, 114.5, 115.4, 124.4, 124.7, 127.0, 129.3,

149.7, 158.9 (br), 160.0, 160.4, 161.1, 163.2. Anal. Calcd for $C_{22}H_{20}O_6$: C, 69.46; H, 5.30. Found: C, 69.55; H, 5.21.

Spectral data of 5-(2-hydroxybenzylidene)-1,3-dimethylpyrimidine-2,4,6(1*H*,3*H*,5*H*)-trione (B)[4]

White solid. ^{1}H NMR (700 MHz, $CDCl_3$): δ (ppm) 3.42 (s, 3H), 3.44 (s, 3H), 7.03 (t, 1H, *J* = 7.8 Hz), 7.06 (d, 1H, *J* = 8.2 Hz), 7.47 (t, 1H, *J* = 8.4 Hz), 7.63 (d, 1H, *J* = 7.8 Hz), 8.76 (s, 1H). ^{13}C NMR (176 MHz, $CDCl_3$) δ (ppm) 28.8, 29.2, 115.9, 120.1, 120.9, 122.8, 135.5, 135.7, 150.7, 157.0, 158.9, 162.1, 163.7.

S-5. Reference

1. Dolomanov, O.; V., Bourhis, L. J.; Gildea, R. J.; Howard, J. A. K.; & Puschmann, H. *J. Appl. Cryst.* **2009**, *42*, 339-341.
2. Bourhis, L. J.; Dolomanov, O. V.; Gildea, R. J.; Howard, J. A. K.; Puschmann, H. *Acta Cryst*. **2015**, *A71*, 59-75.
3. Sheldrick, G. M. *Acta Cryst*. **2015**, *C71*, 3-8.
4. Tanwer, Y. B. S.; Sangma, S. W.; Patra, S. R.; Swain, K.; Bhunia, S.; Pal, S.; Das, D. *ACS Sustainable Resour. Manage*. **2025**, *2*, 492-500.

S-6. Spectra of products

^{1}H NMR Spectra of 4a at RT

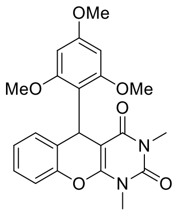

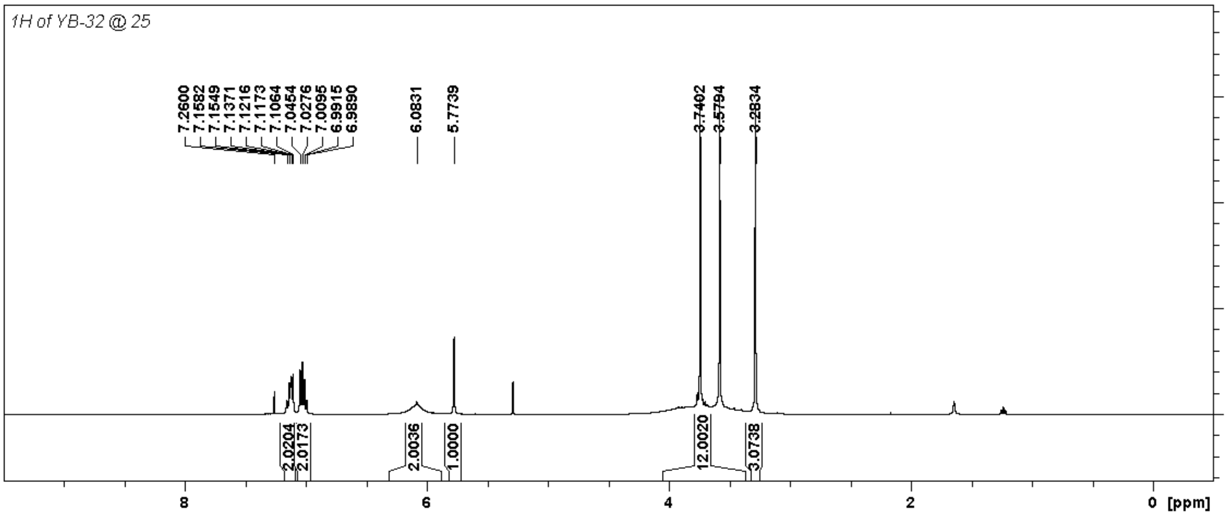

^{1}H NMR Spectra of 4a at 0 ^{o}C

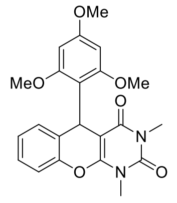

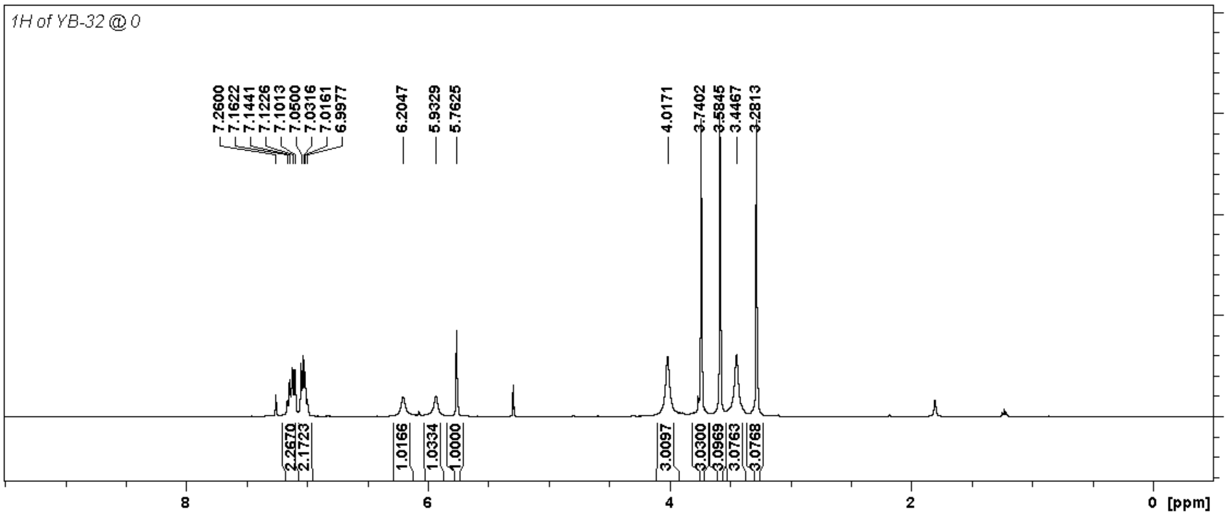

^{1}H NMR Spectra of 4a at -10 ^{o}C

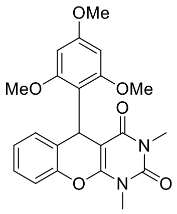

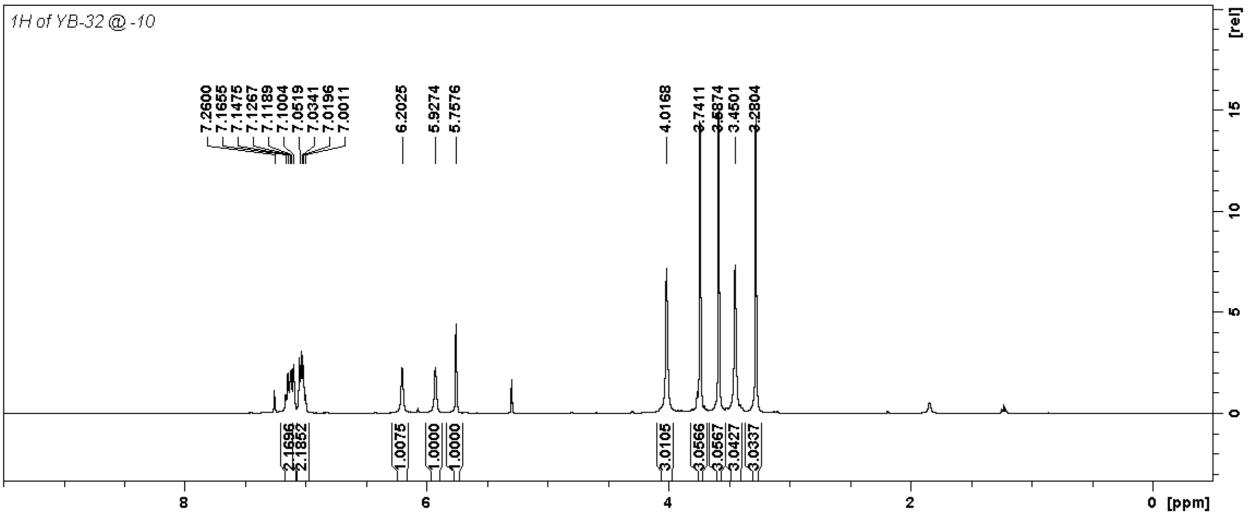

^{1}H NMR Spectra of 4a at -50 ^{o}C

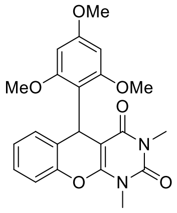

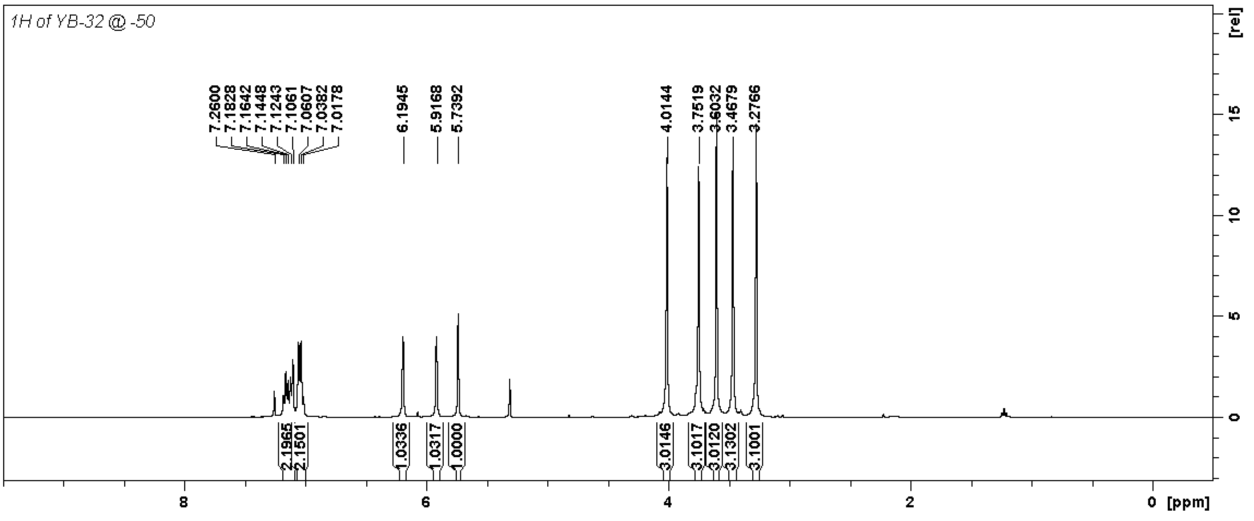

^{13}C NMR Spectra of 4a at RT

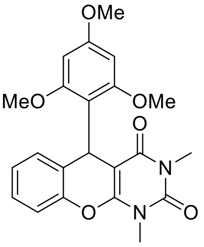

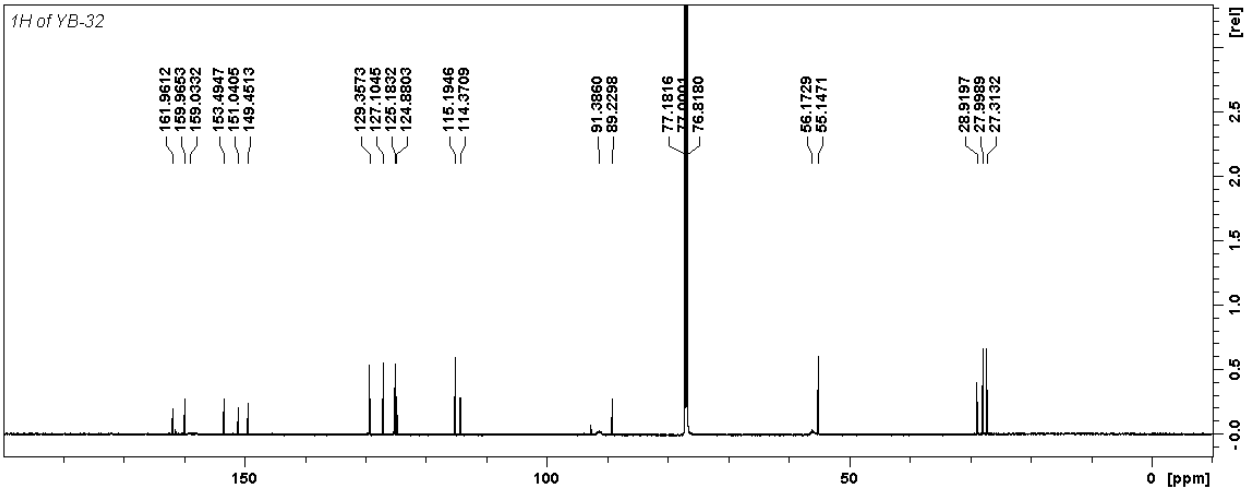

^{13}C NMR Spectra of 4a at -50 ^{o}C

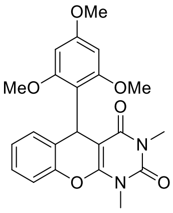

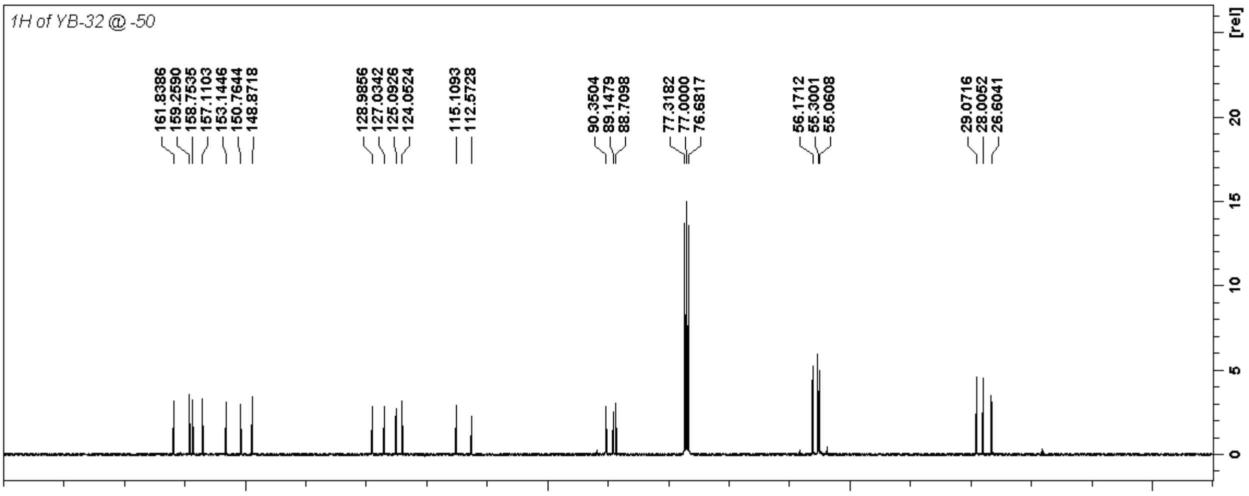

^{1}H NMR Spectra of 4b

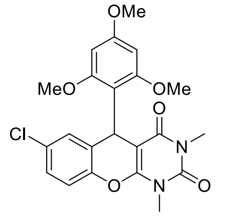

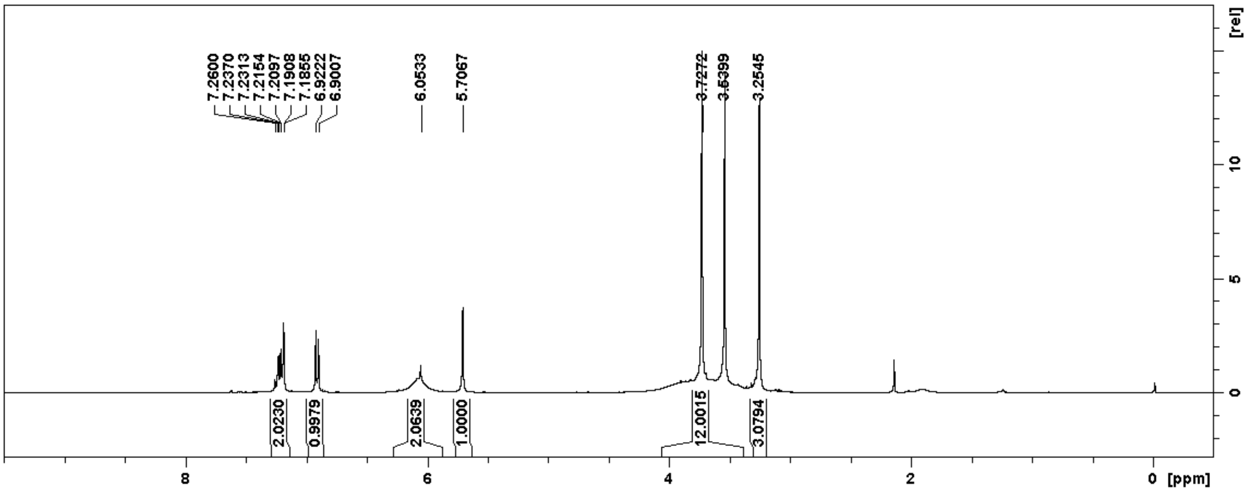

^{13}C NMR Spectra of 4b

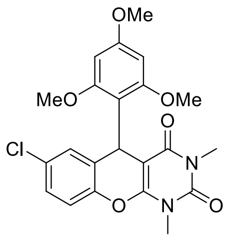

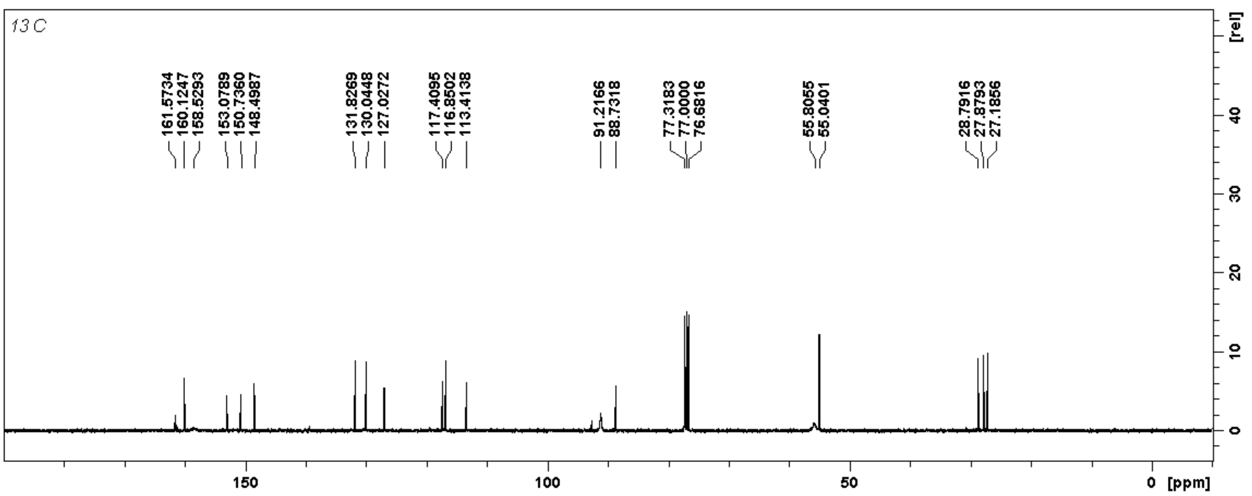

^{1}H NMR Spectra of 4c

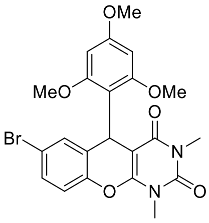

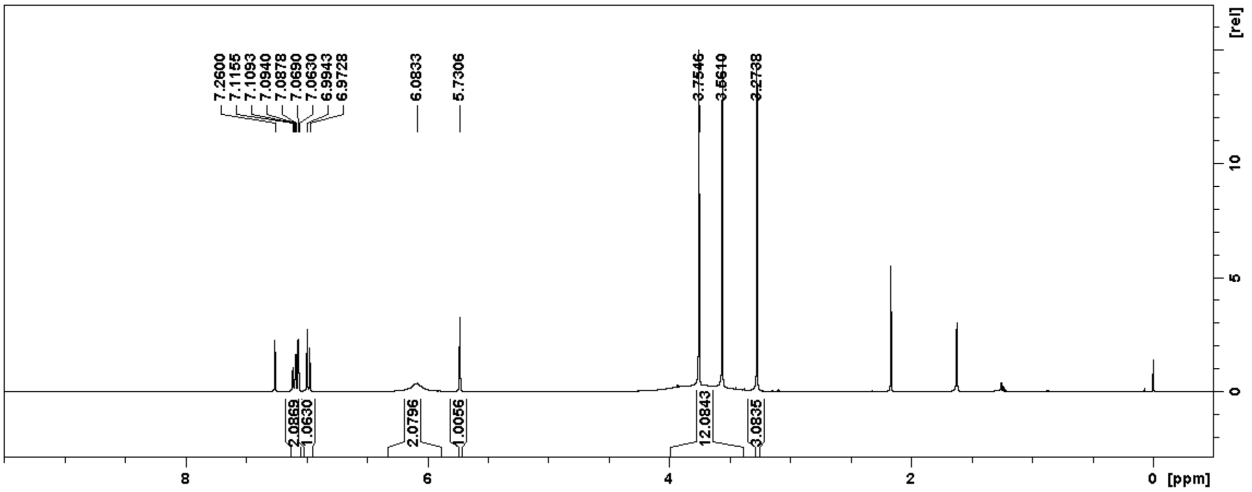

^{13}C NMR Spectra of 4c

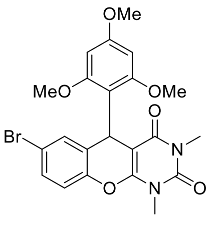

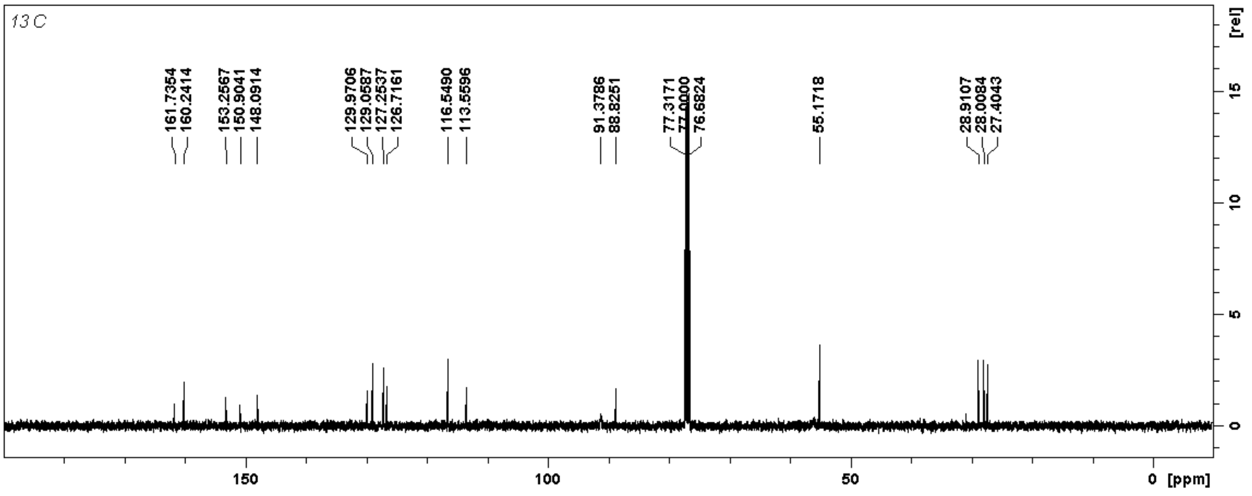

^{1}H NMR Spectra of 4d

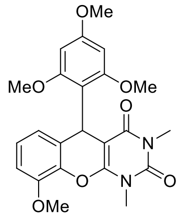

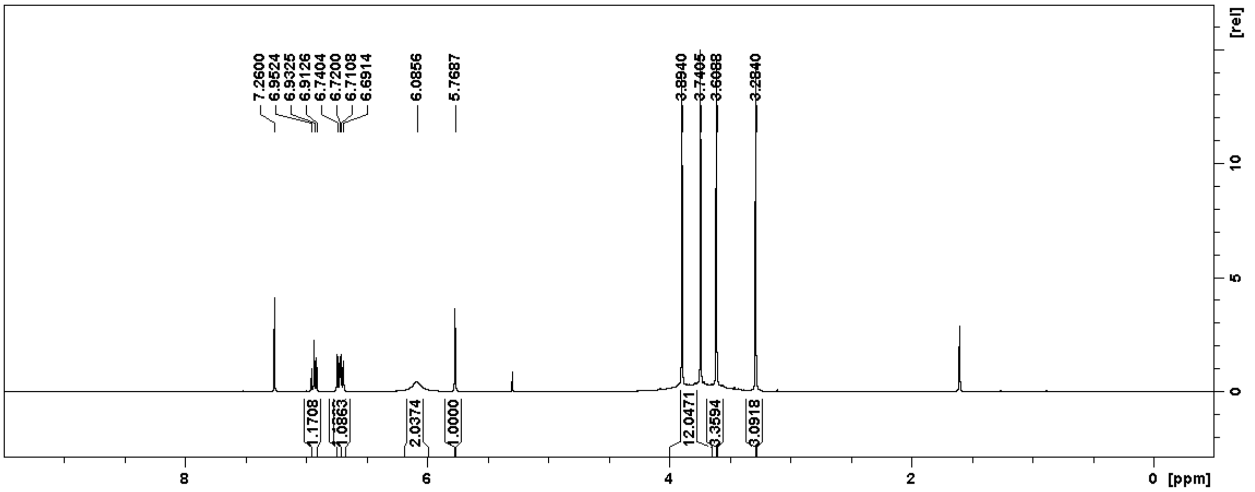

^{13}C NMR Spectra of 4d

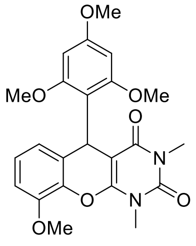

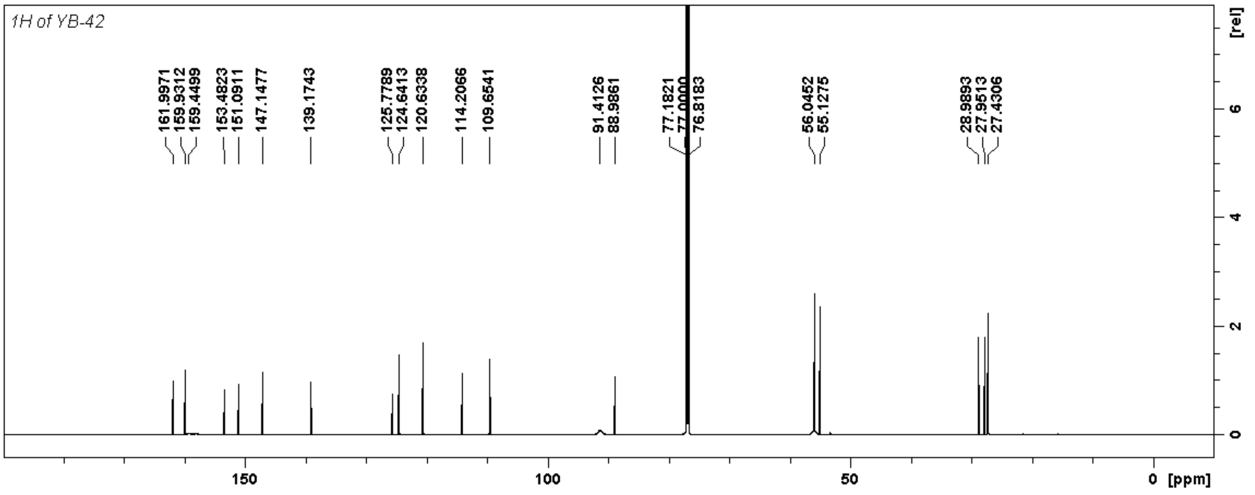

^{1}H NMR Spectra of 4e

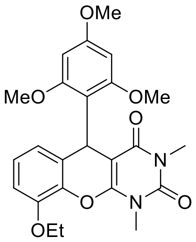

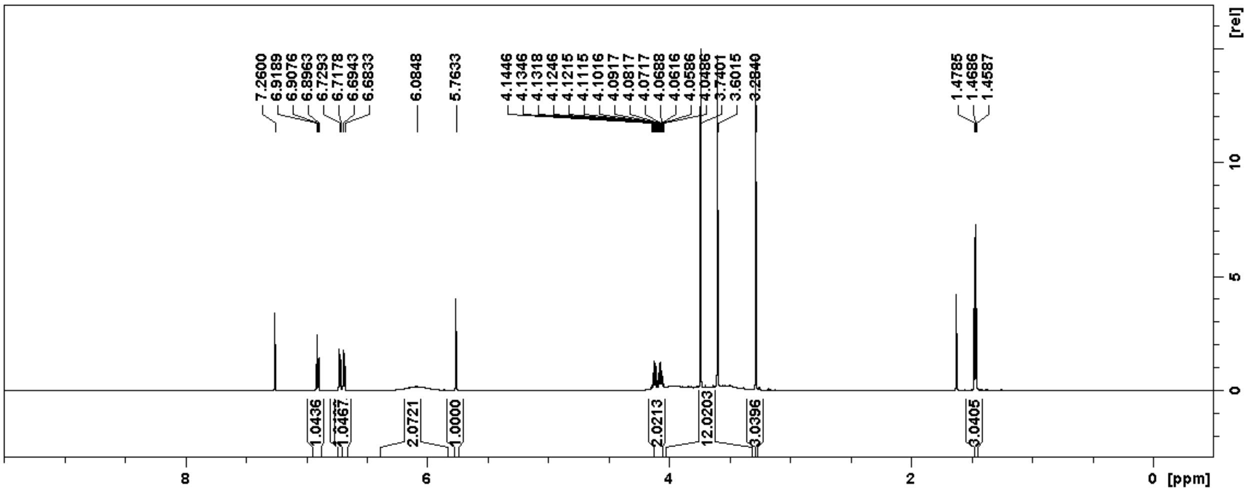

^{13}C NMR Spectra of 4e

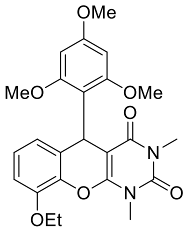

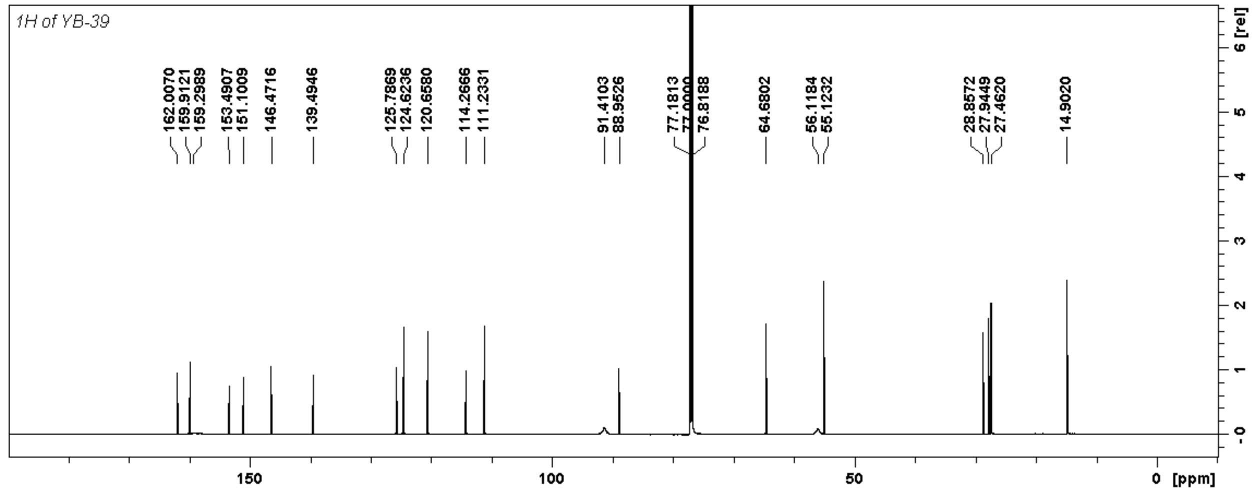

^{1}H NMR Spectra of 4f

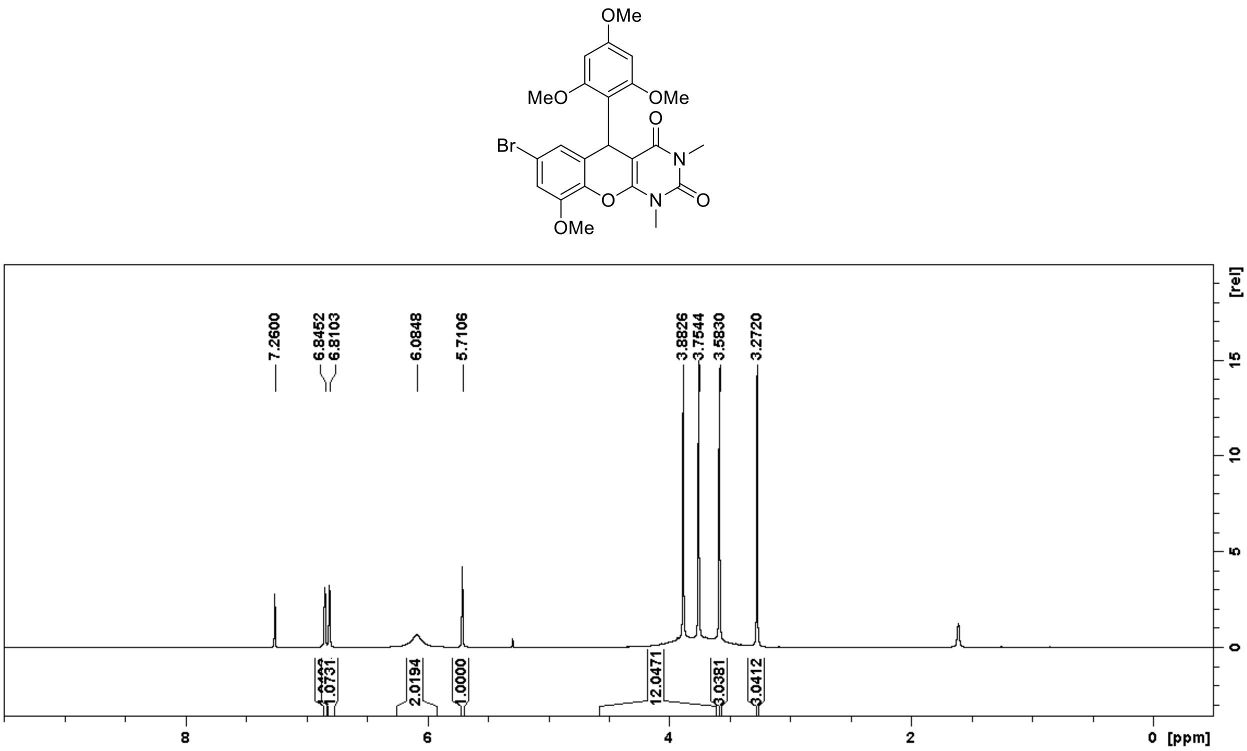

^{13}C NMR Spectra of 4f

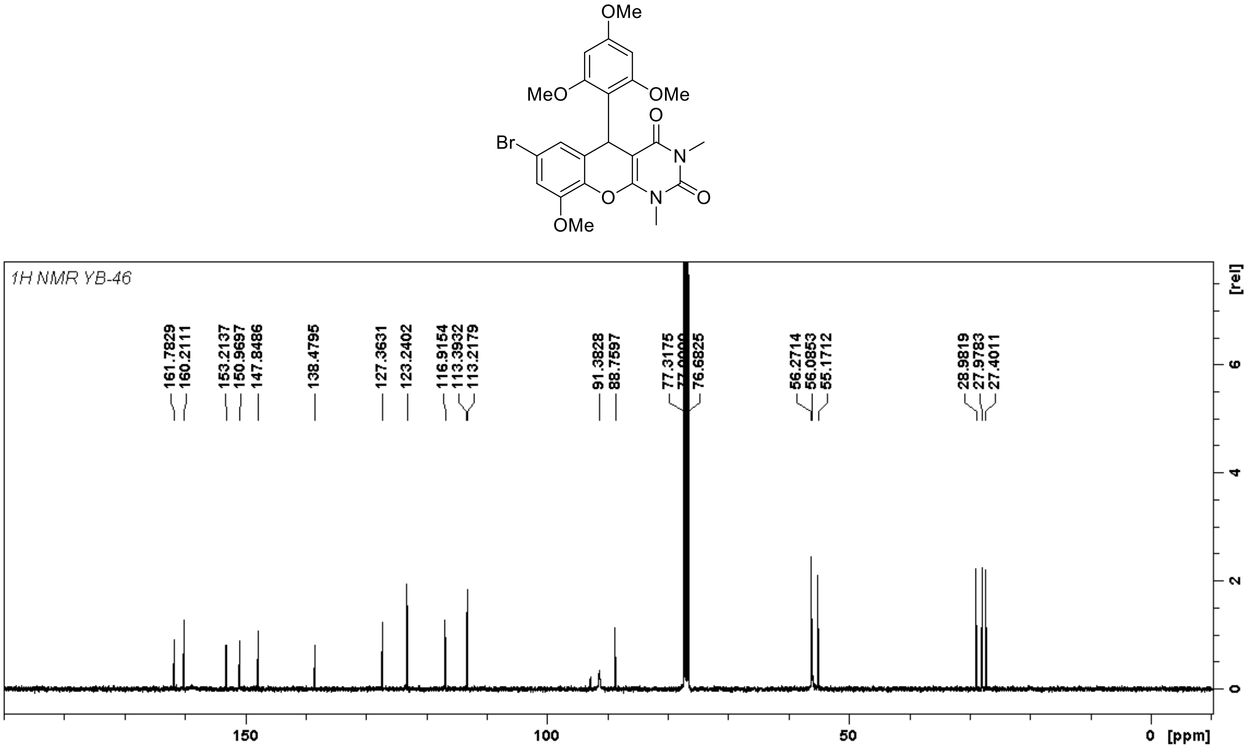

^{1}H NMR Spectra of 4g

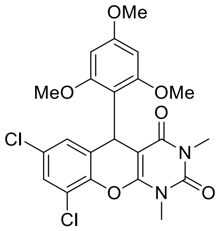

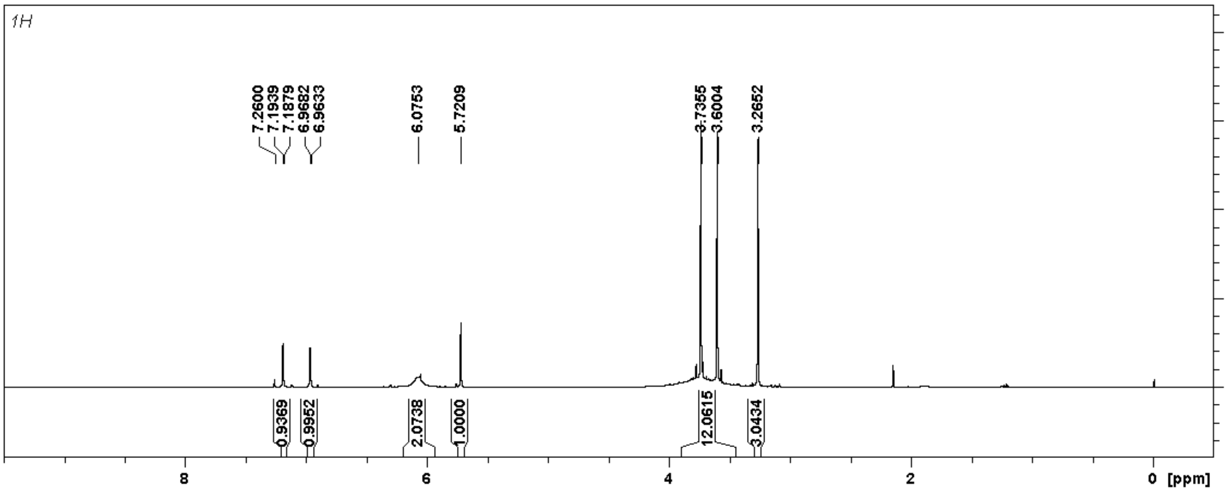

^{13}C NMR Spectra of 4g

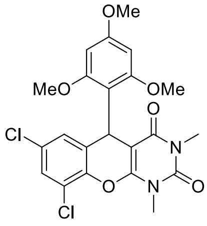

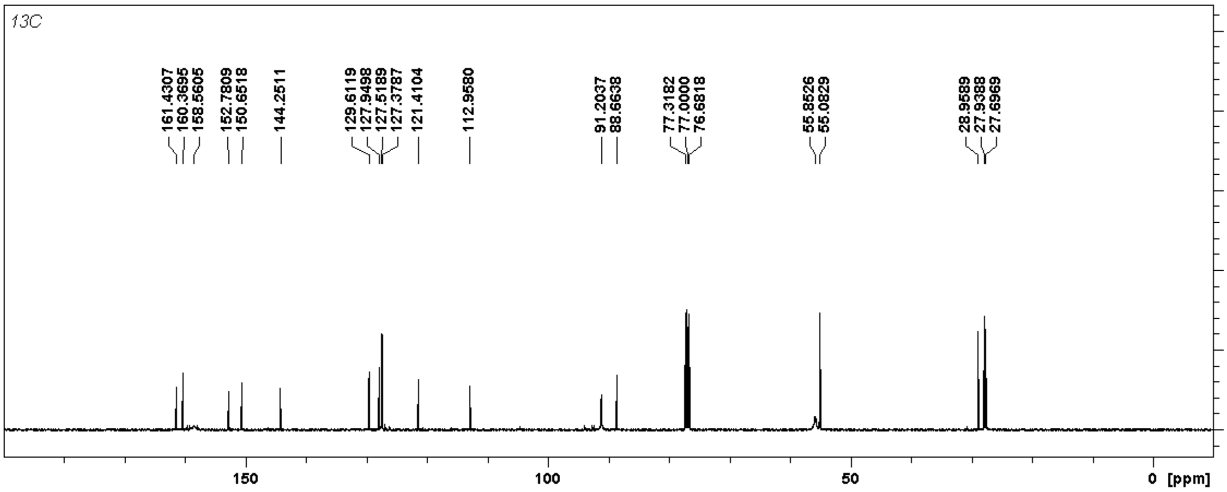

^{1}H NMR Spectra of 4h

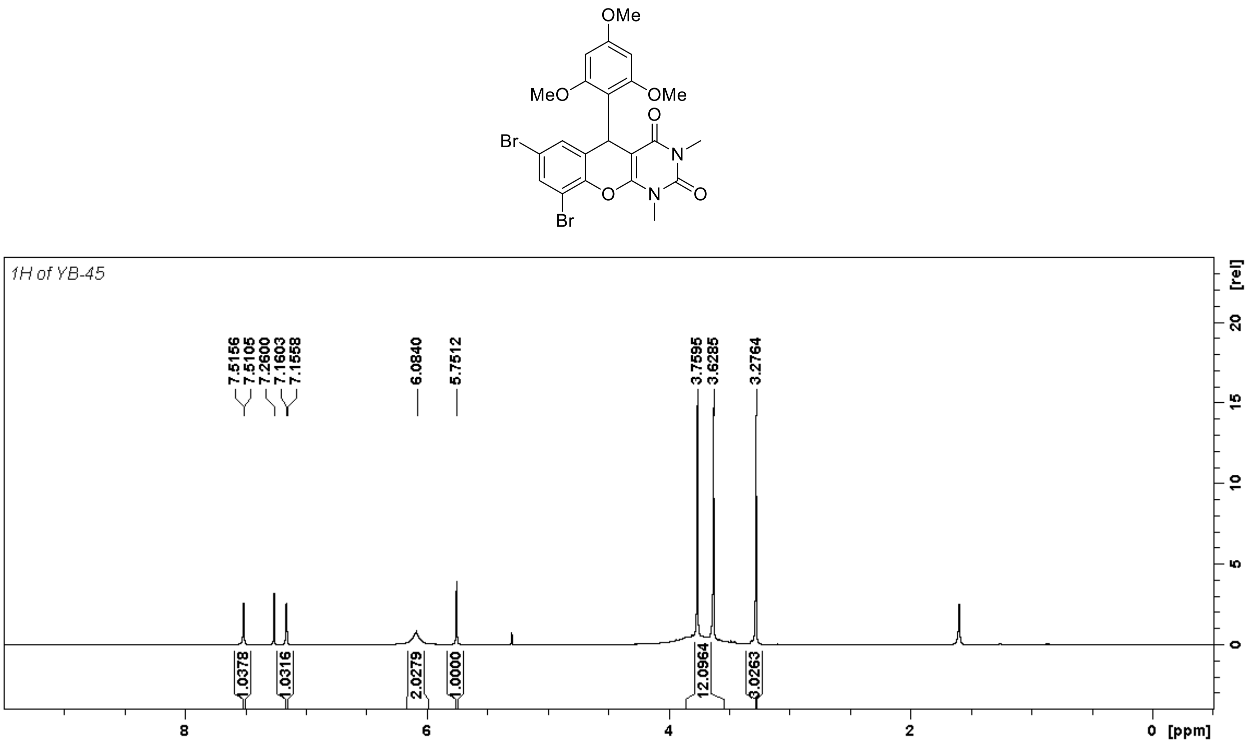

^{13}C NMR Spectra of 4h

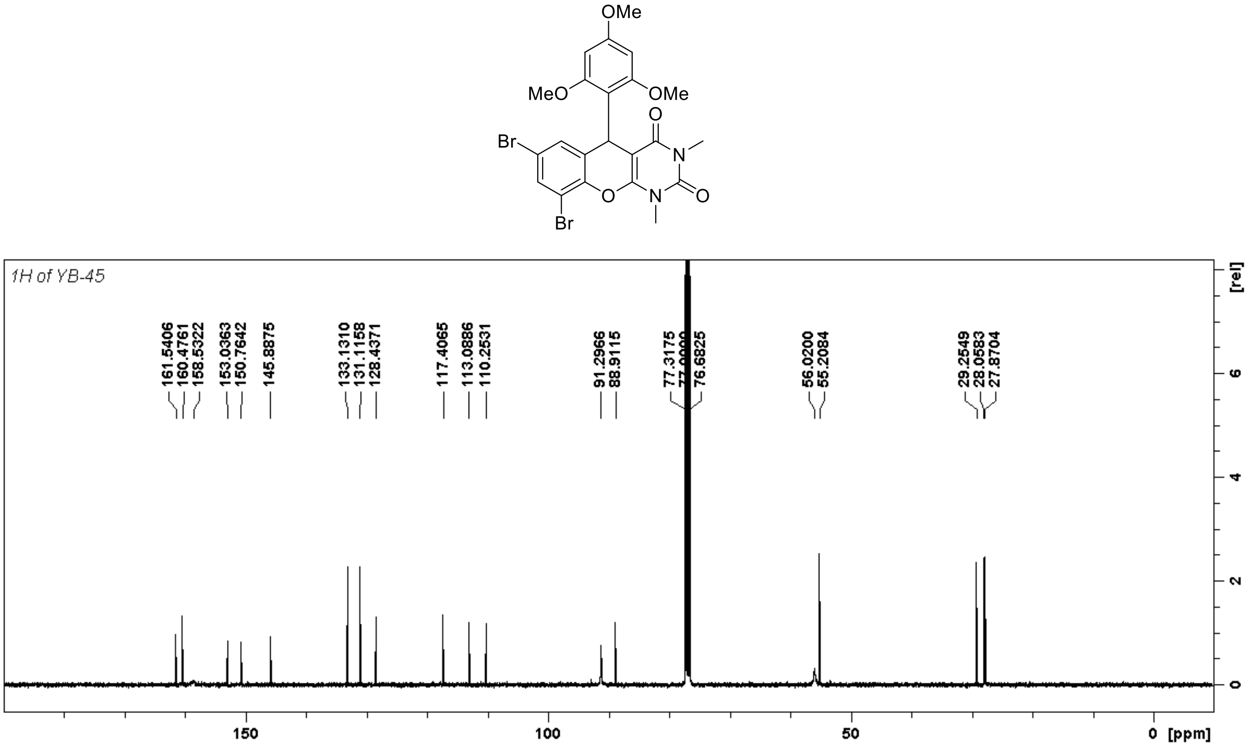

^{1}H NMR Spectra of 4i

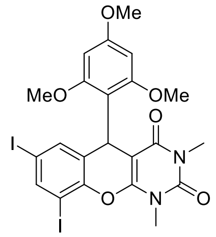

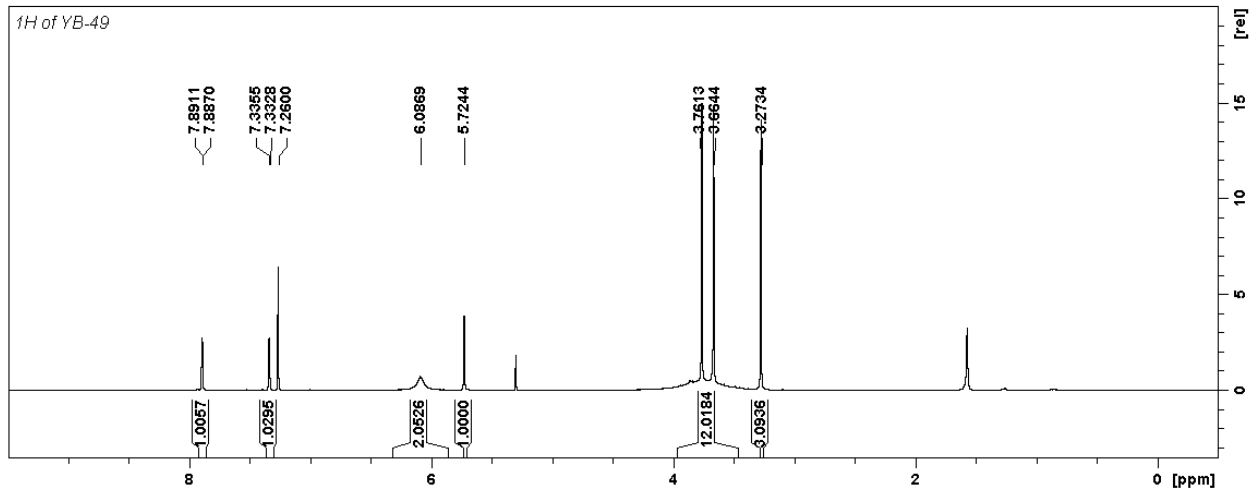

^{13}C NMR Spectra of 4i

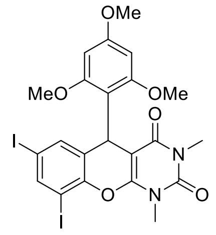

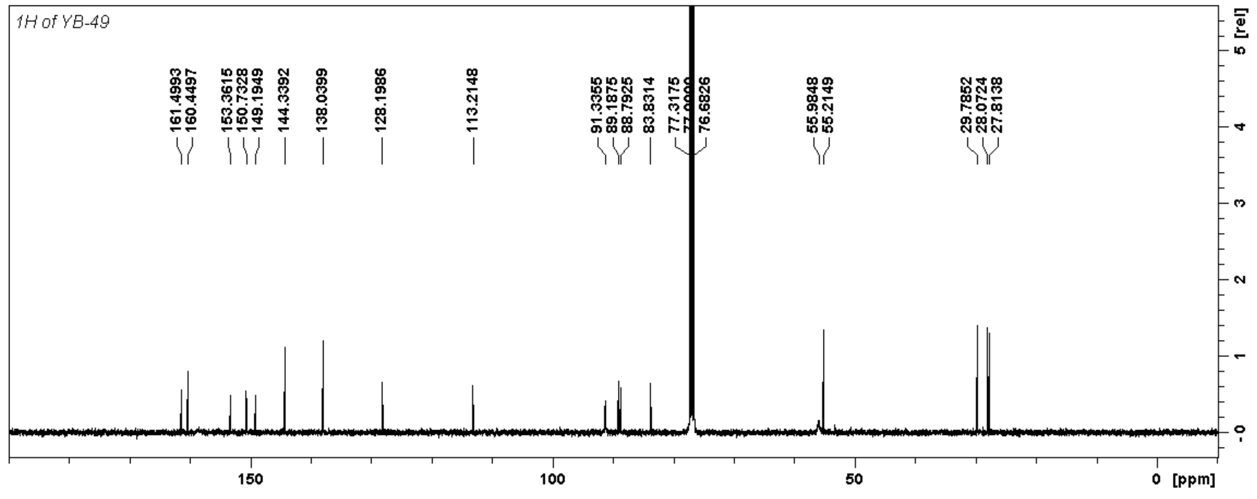

^{1}H NMR Spectra of 4j

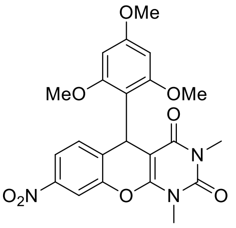

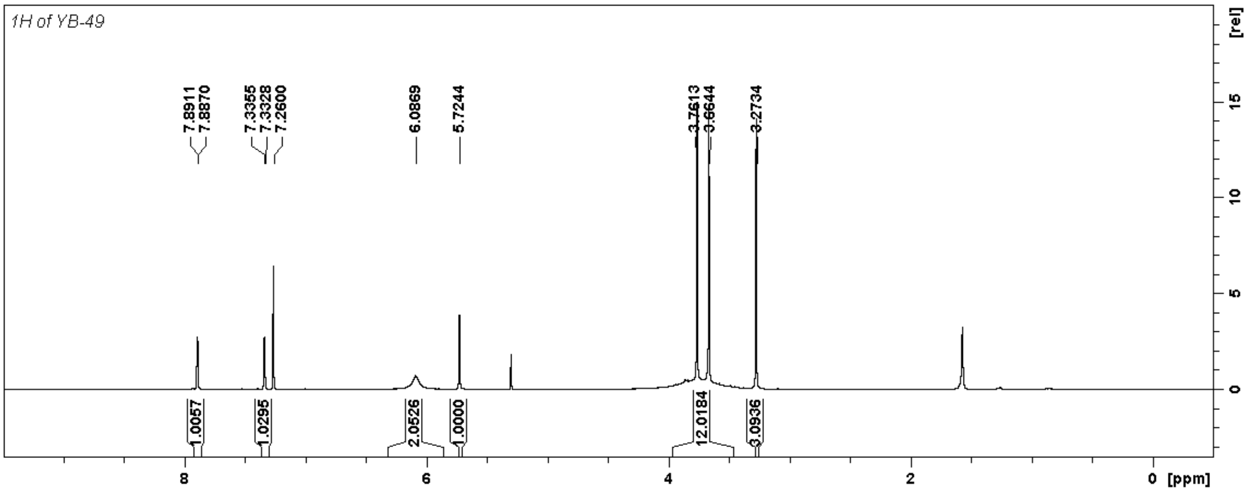

^{13}C NMR Spectra of 4j

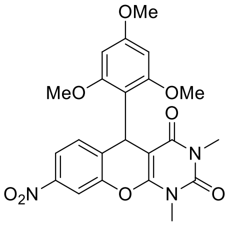

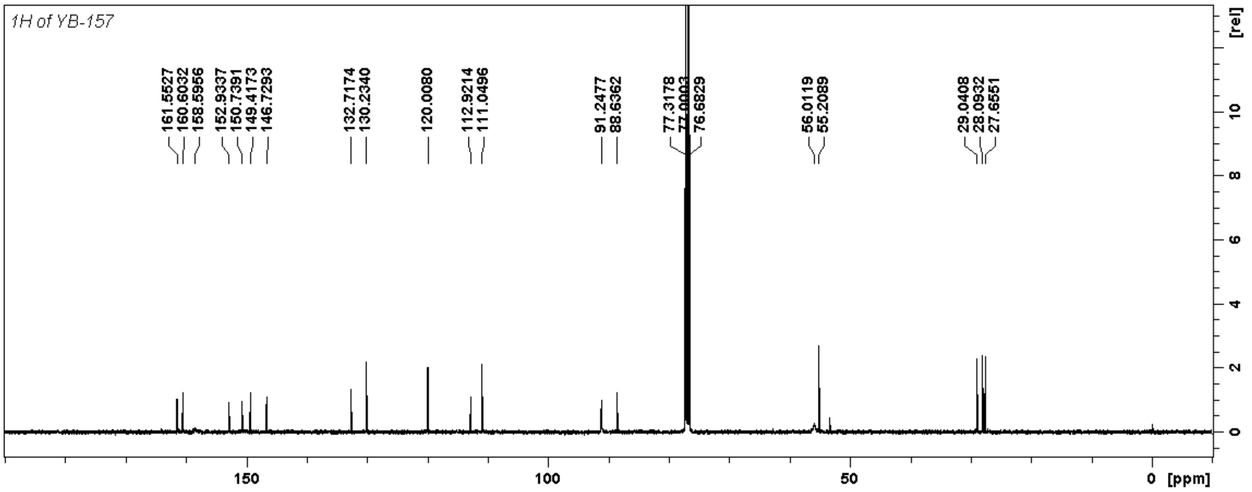

^{1}H NMR Spectra of 4k

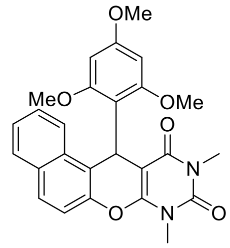

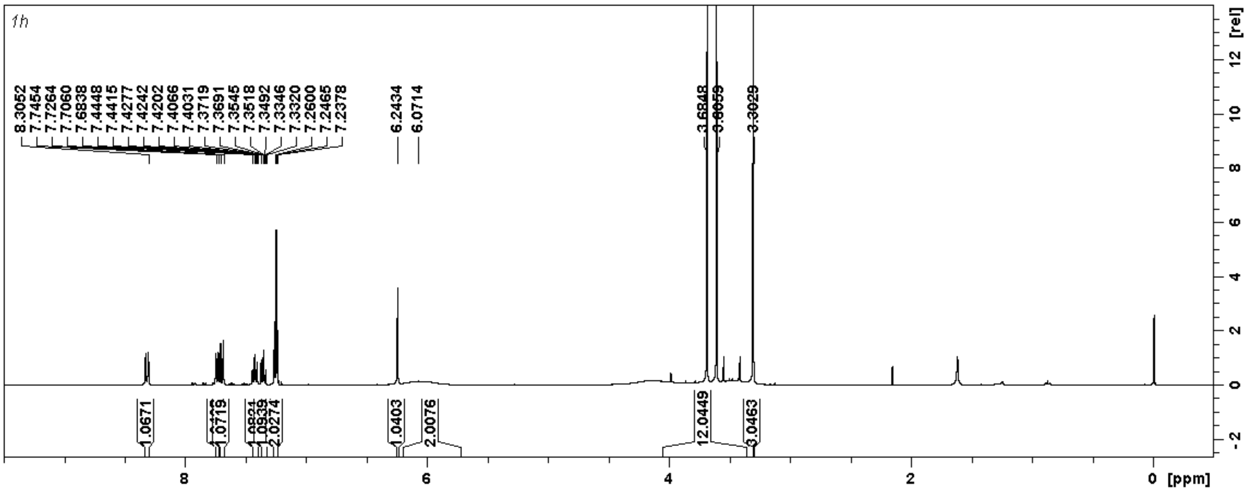

^{13}C NMR Spectra of 4k

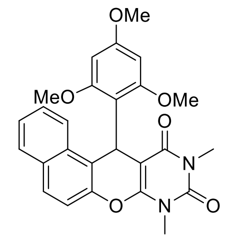

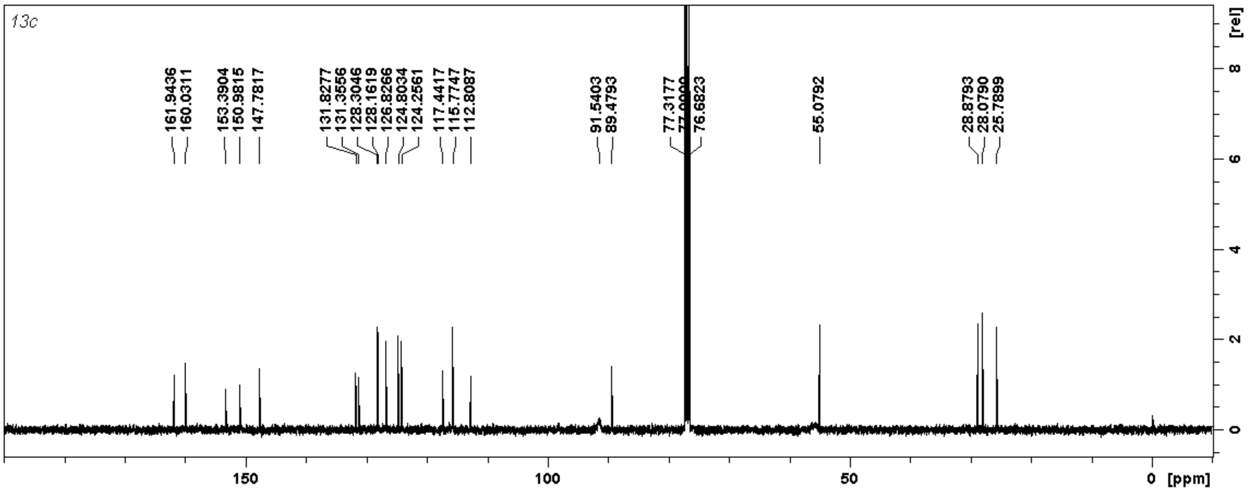

^{1}H NMR Spectra of 4l

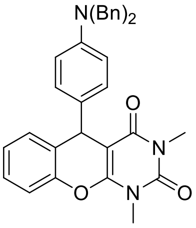

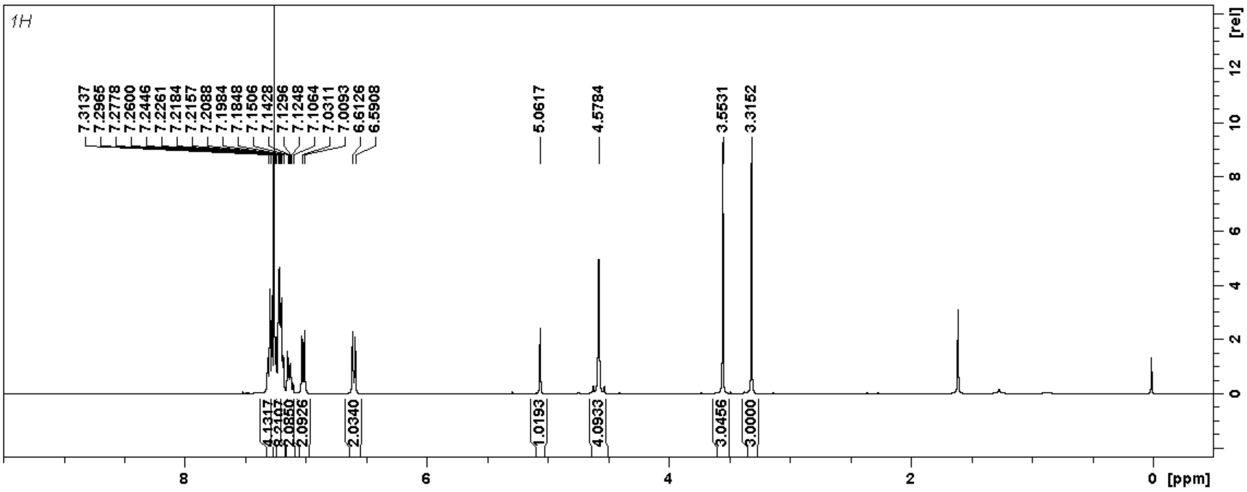

^{13}C NMR Spectra of 4l

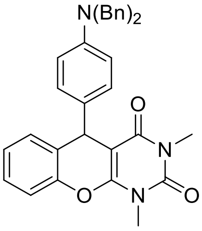

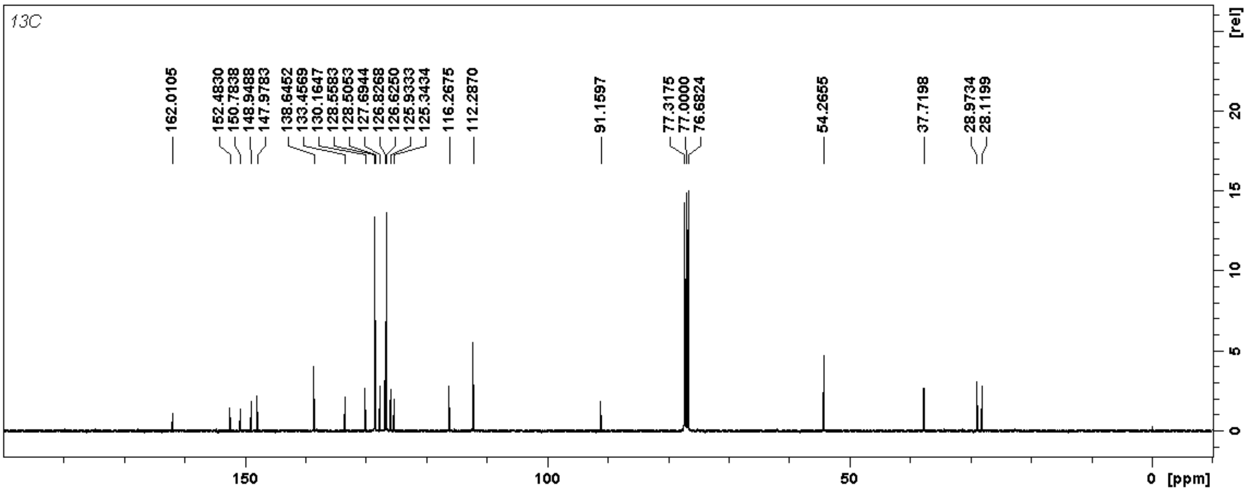

^{1}H NMR Spectra of 5a

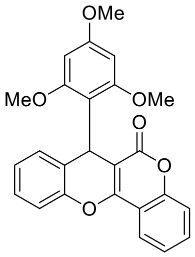

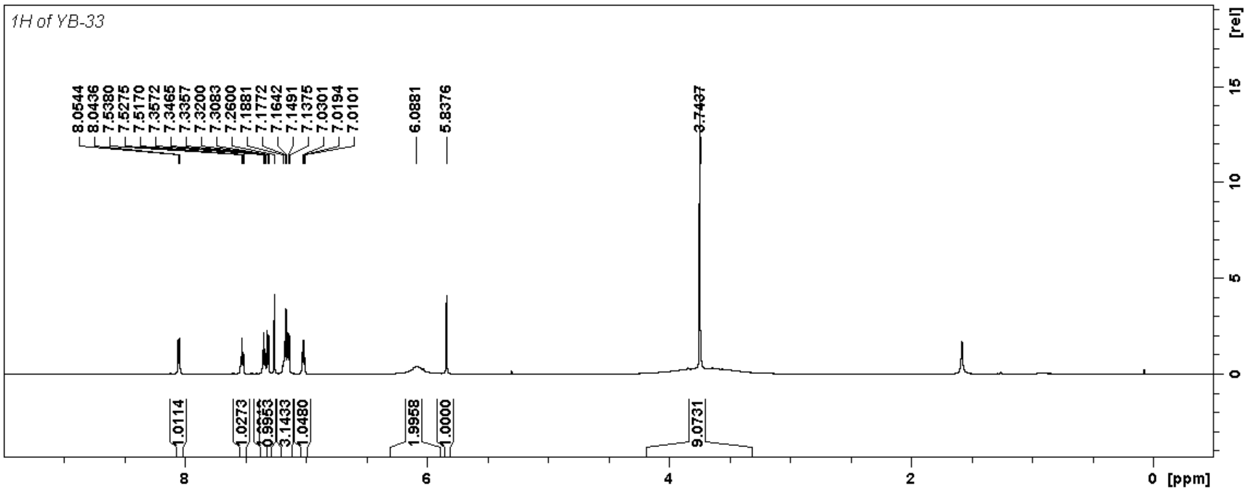

^{13}C NMR Spectra of 5a

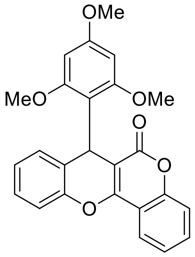

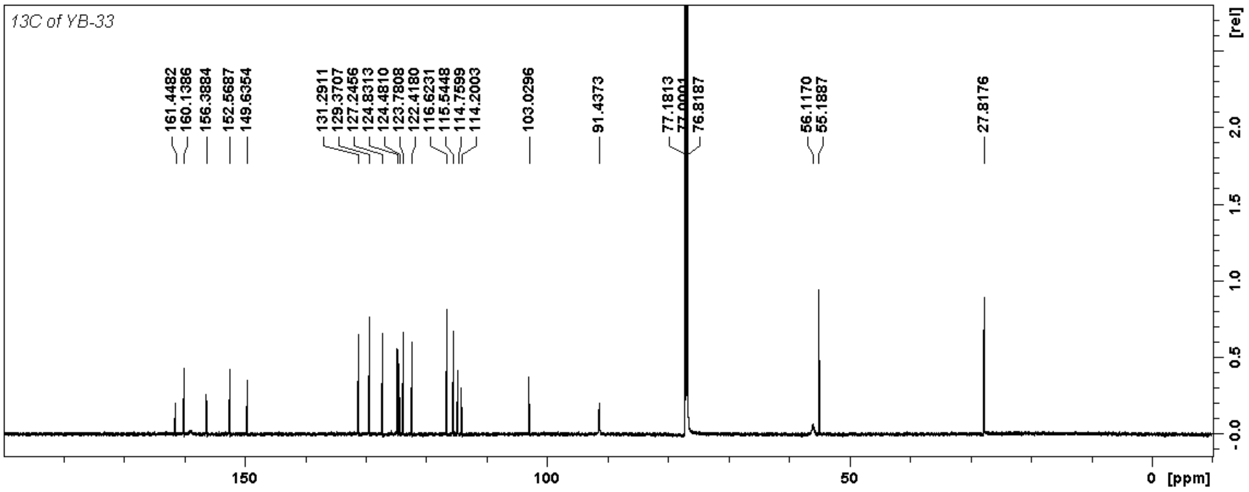

^{1}H NMR Spectra of 5b

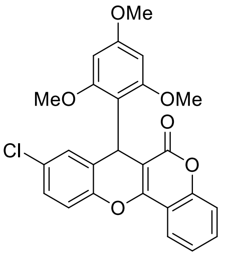

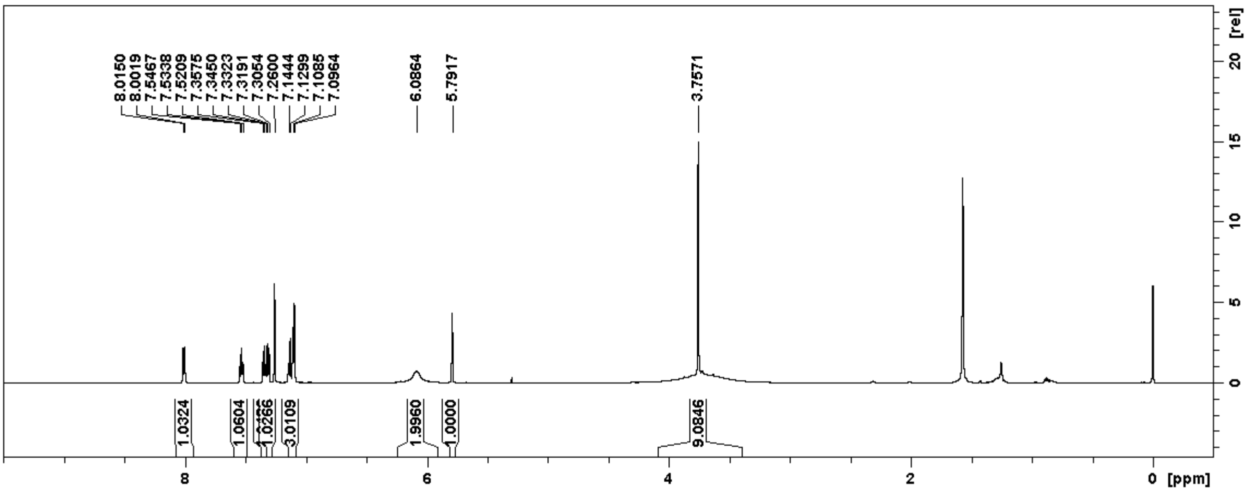

^{13}C NMR Spectra of 5b

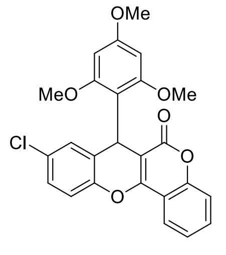

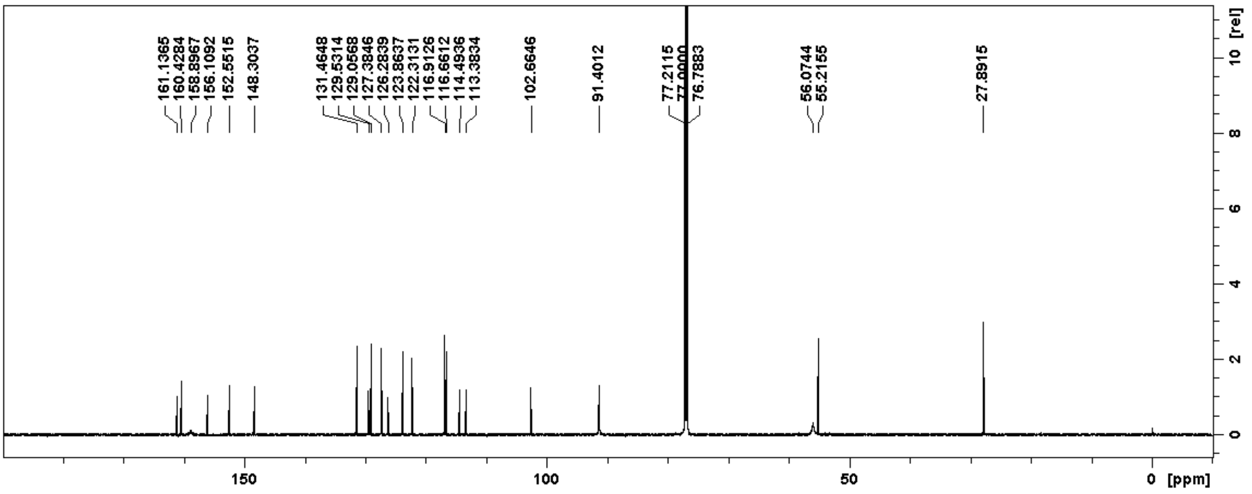

^{1}H NMR Spectra of 5c

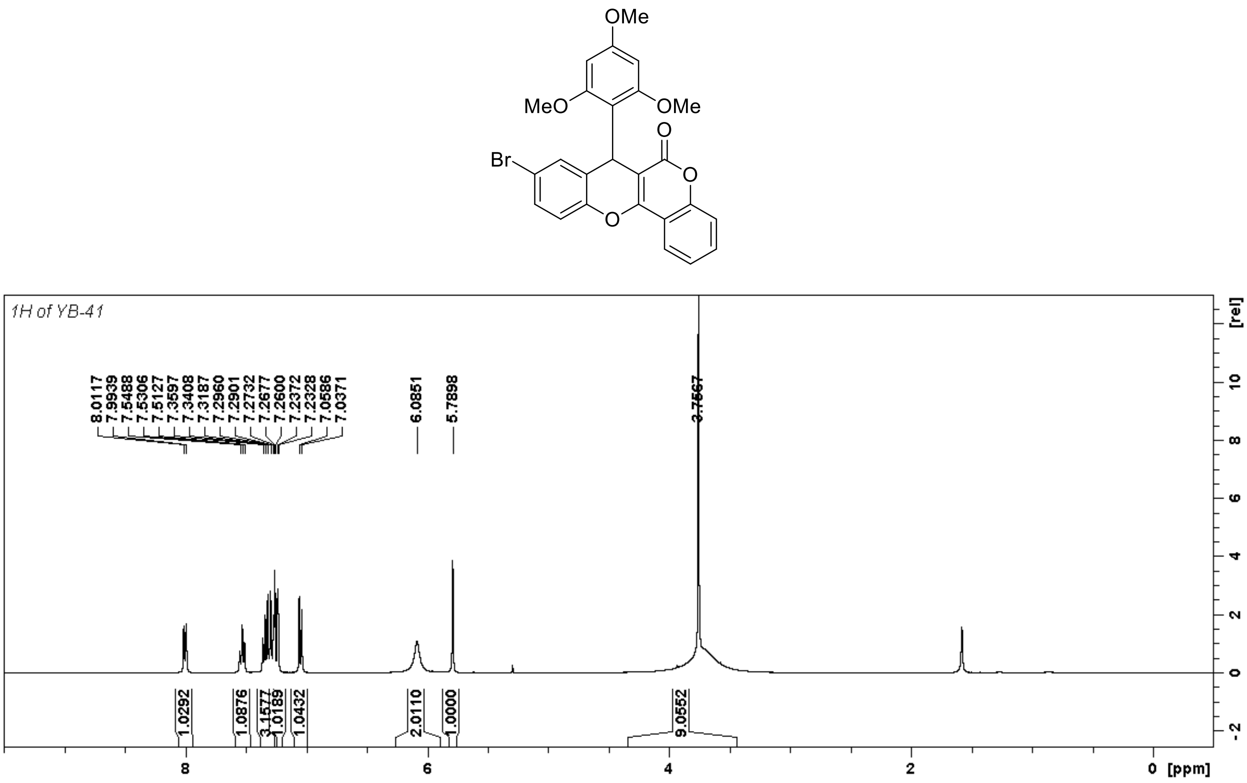

^{13}C NMR Spectra of 5c

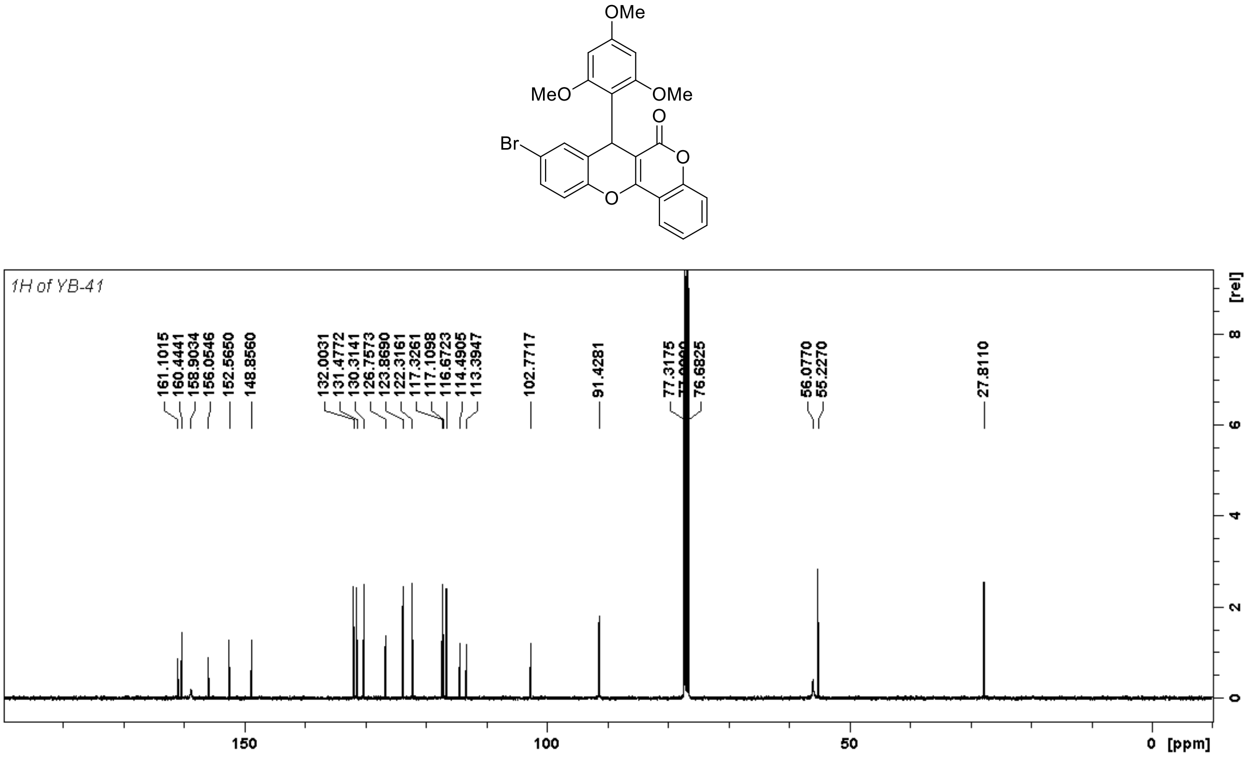

^{1}H NMR Spectra of 5d

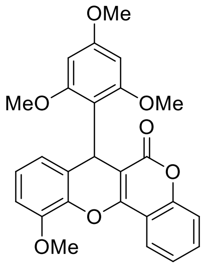

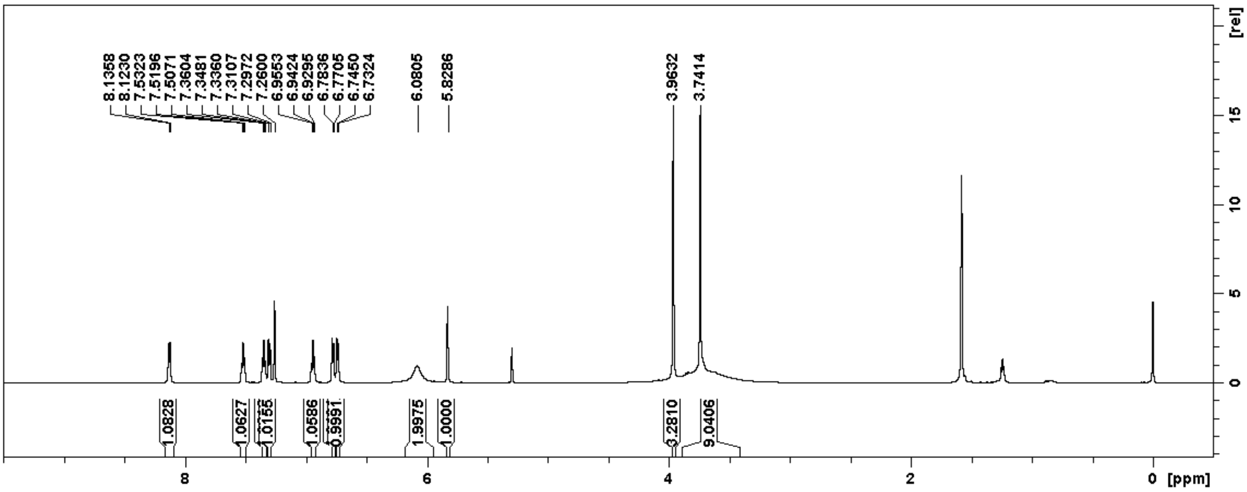

^{13}C NMR Spectra of 5d

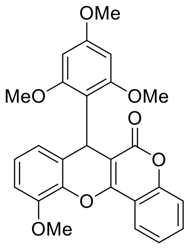

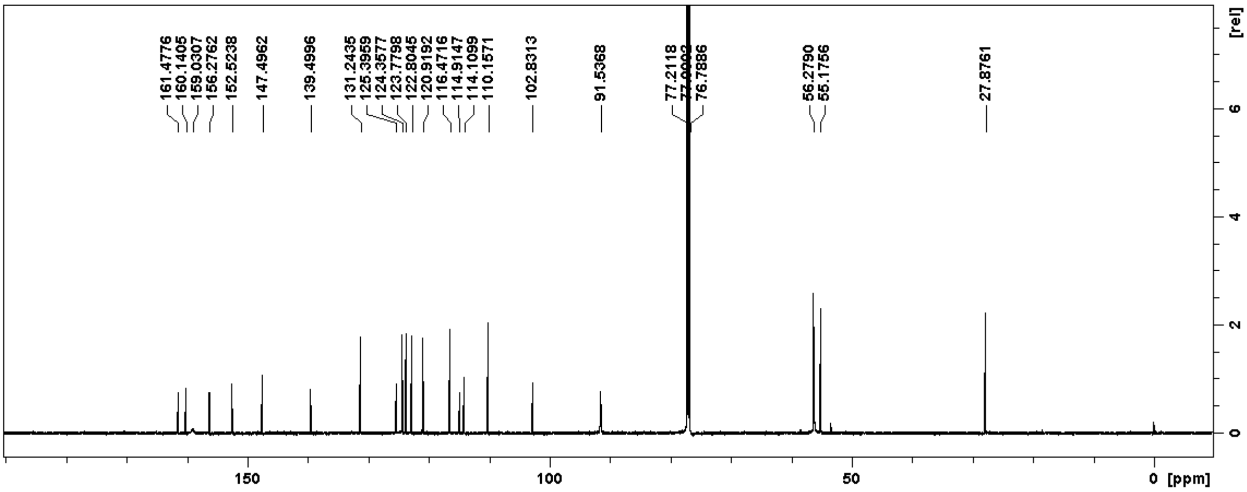

^{1}H NMR Spectra of 5e

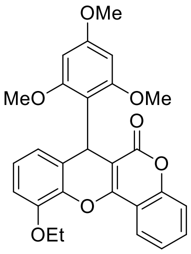

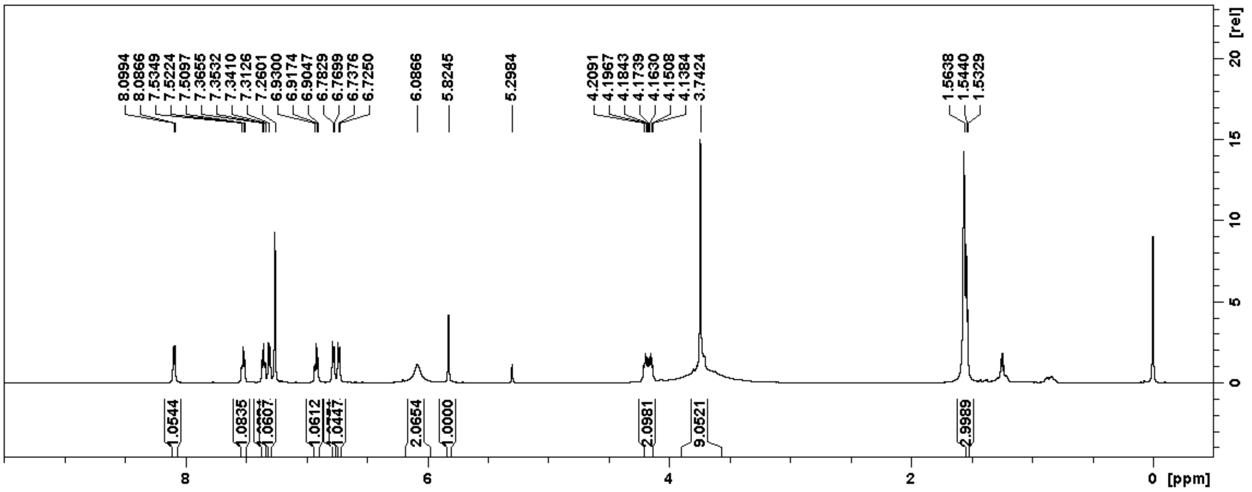

^{13}C NMR Spectra of 5e

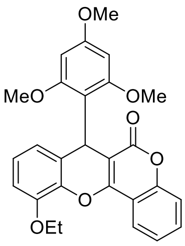

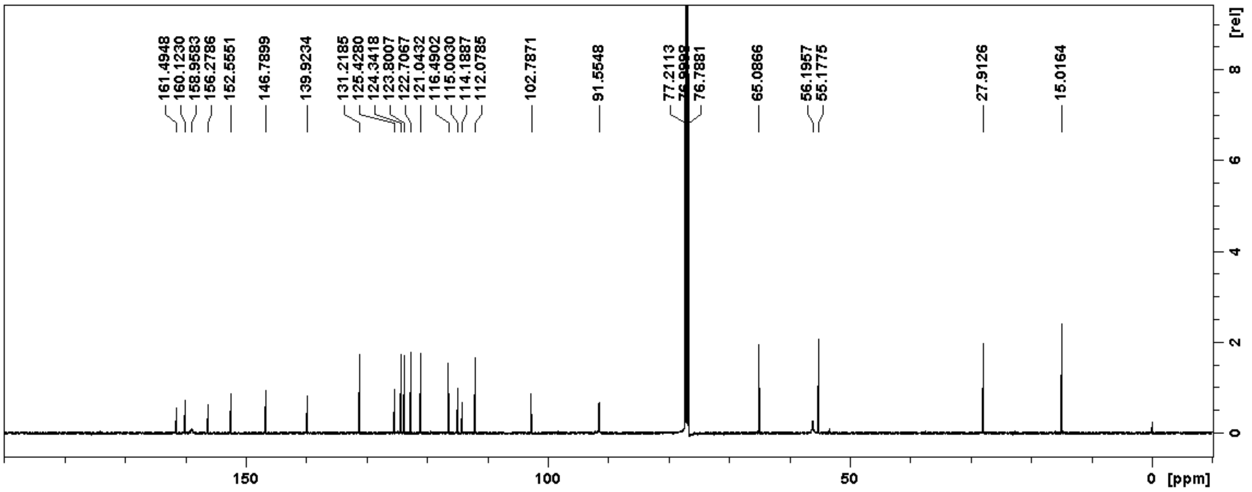

^{1}H NMR Spectra of 5f

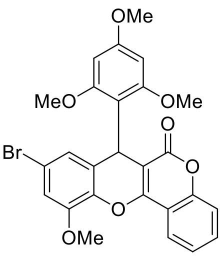

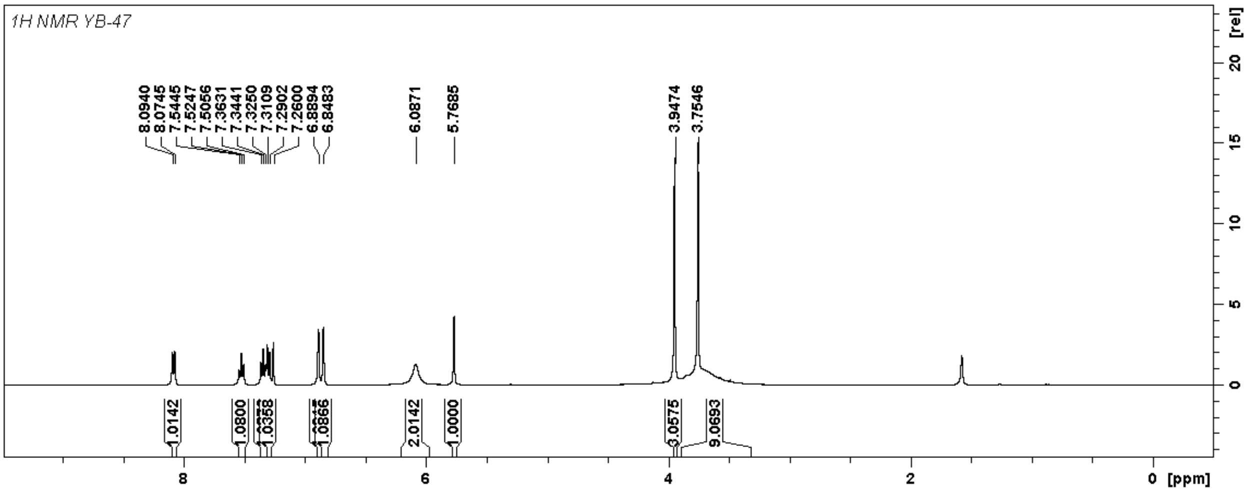

^{13}C NMR Spectra of 5f

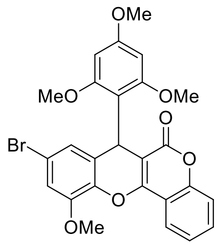

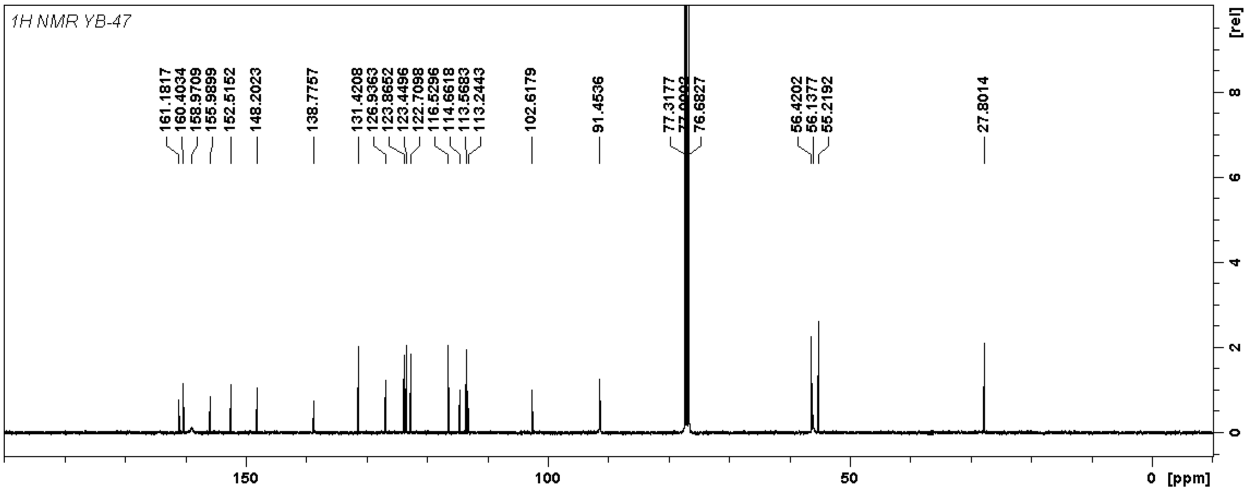

^{1}H NMR Spectra of 5g

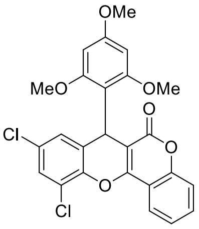

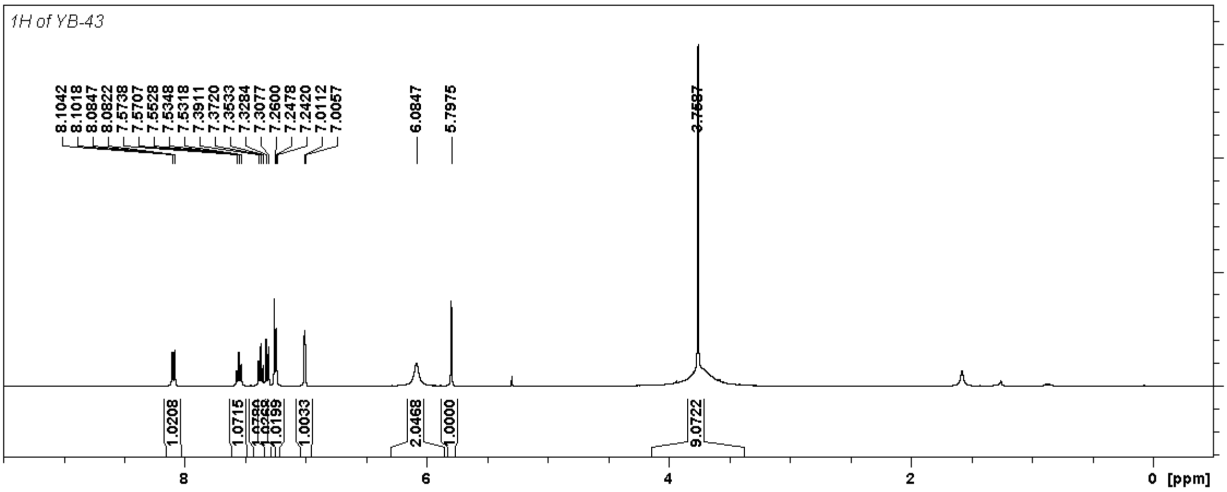

^{13}C NMR Spectra of 5g

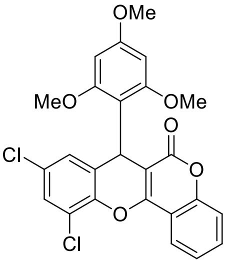

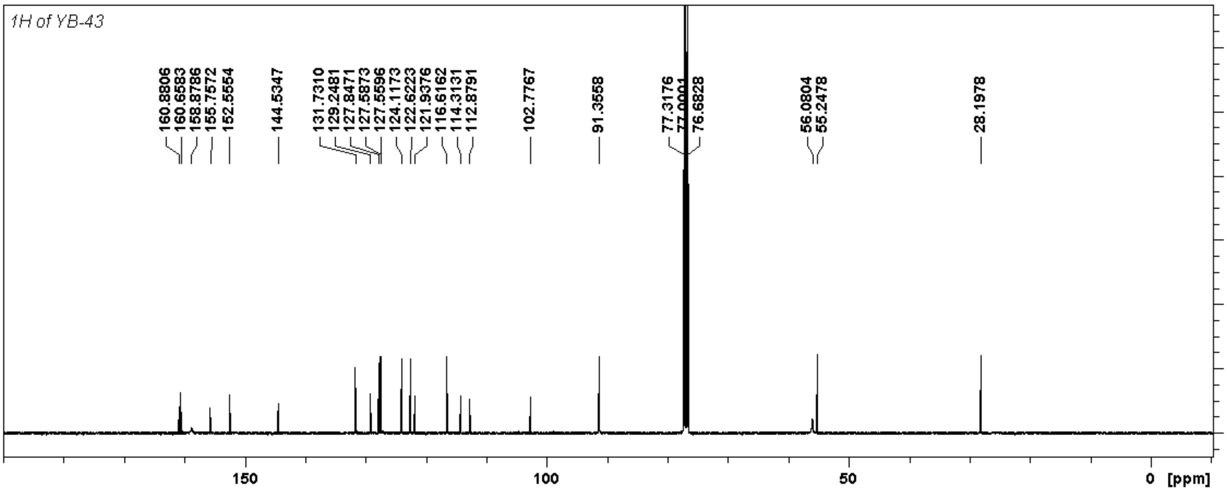

^{1}H NMR Spectra of 5h

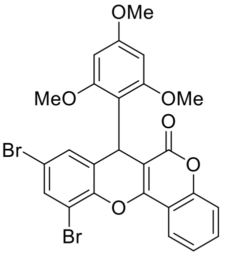

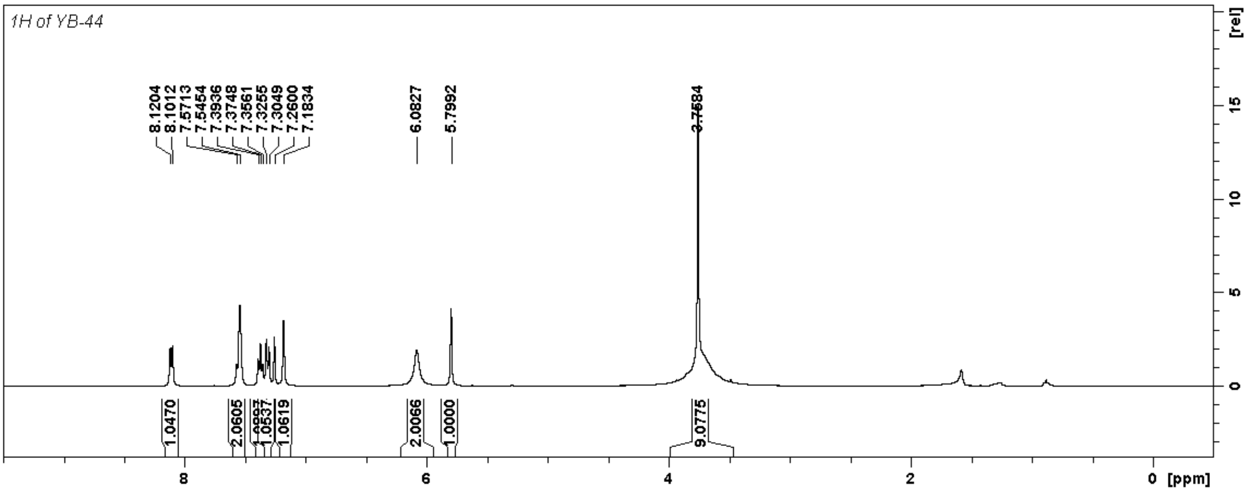

^{13}C NMR Spectra of 5h

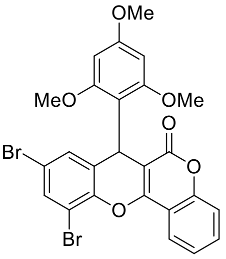

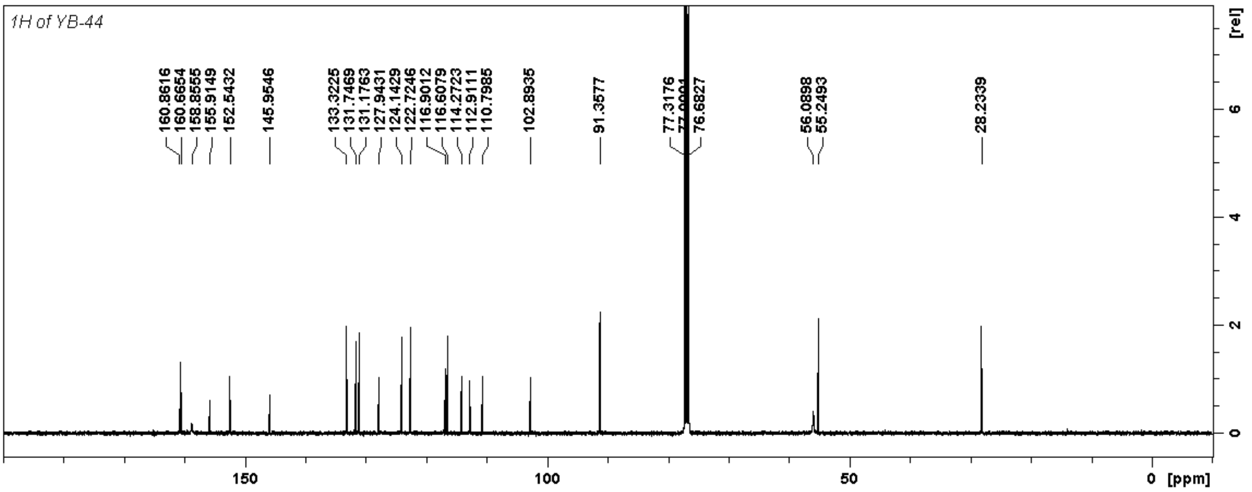

^{1}H NMR Spectra of 5i

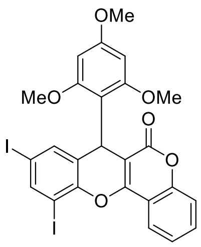

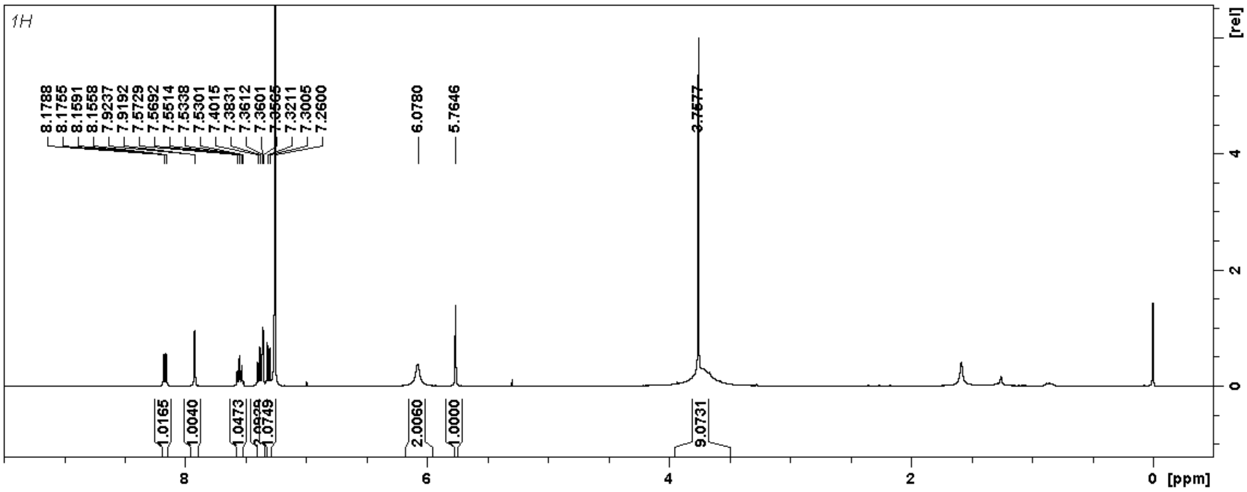

^{13}C NMR Spectra of 5i

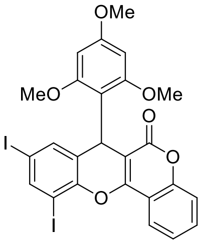

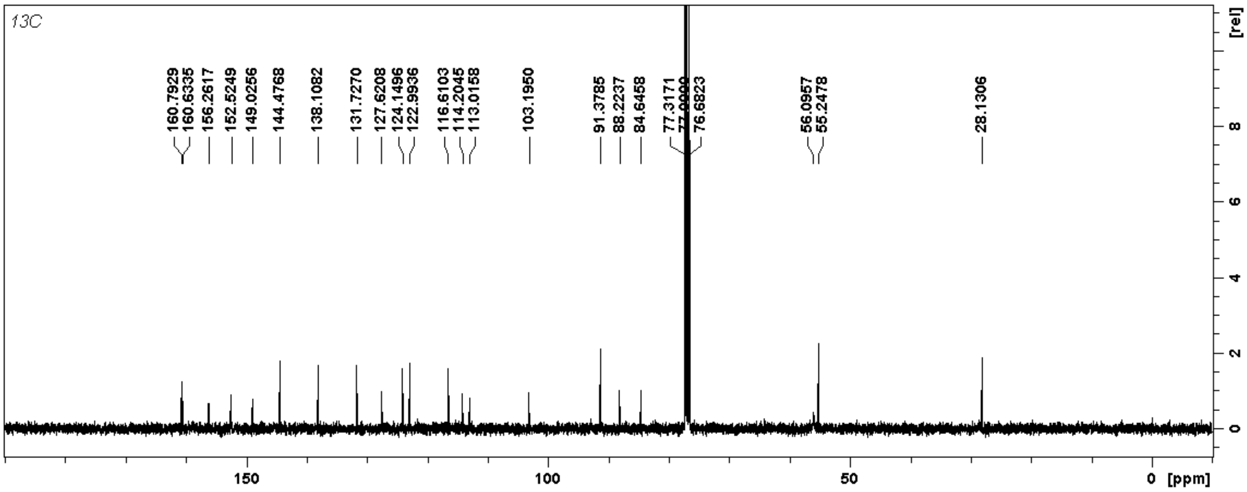

^{1}H NMR Spectra of 5j

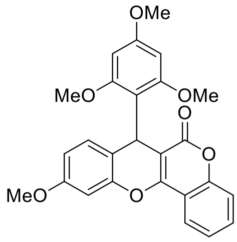

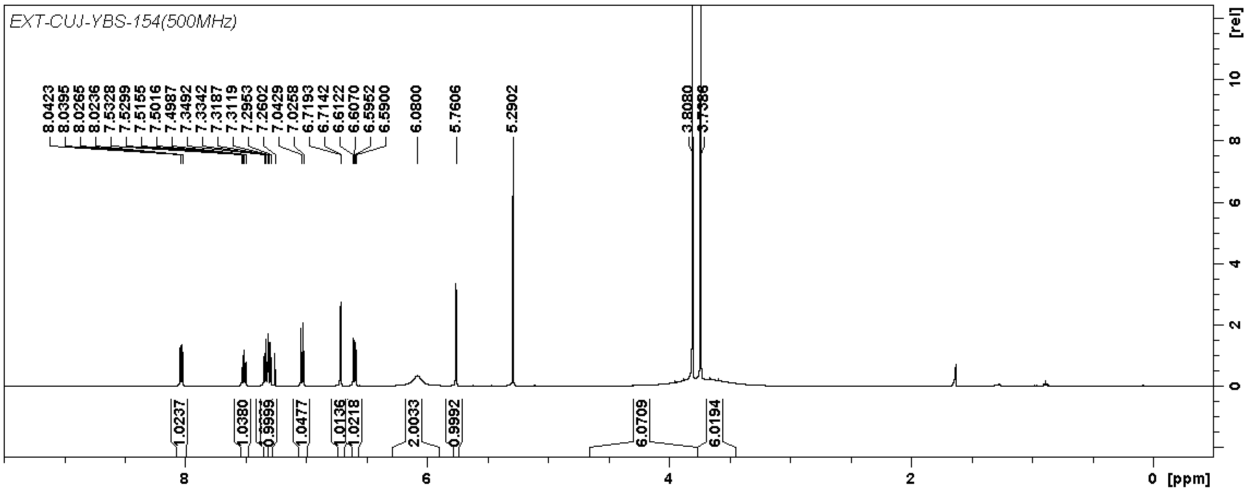

^{13}C NMR Spectra of 5j

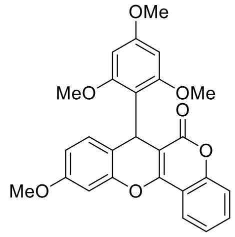

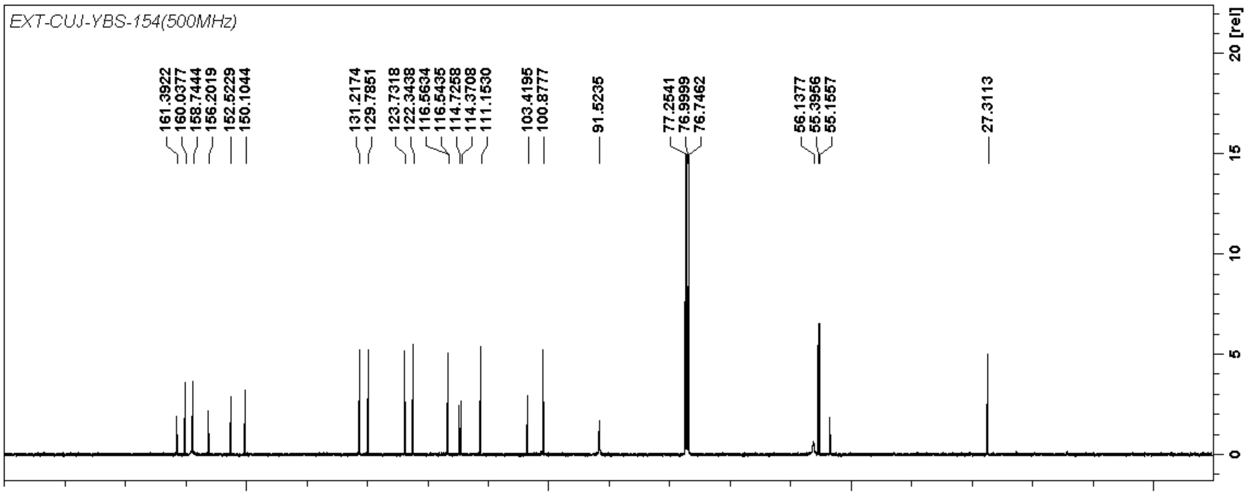

^{1}H NMR Spectra of 5k

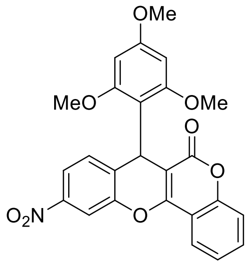

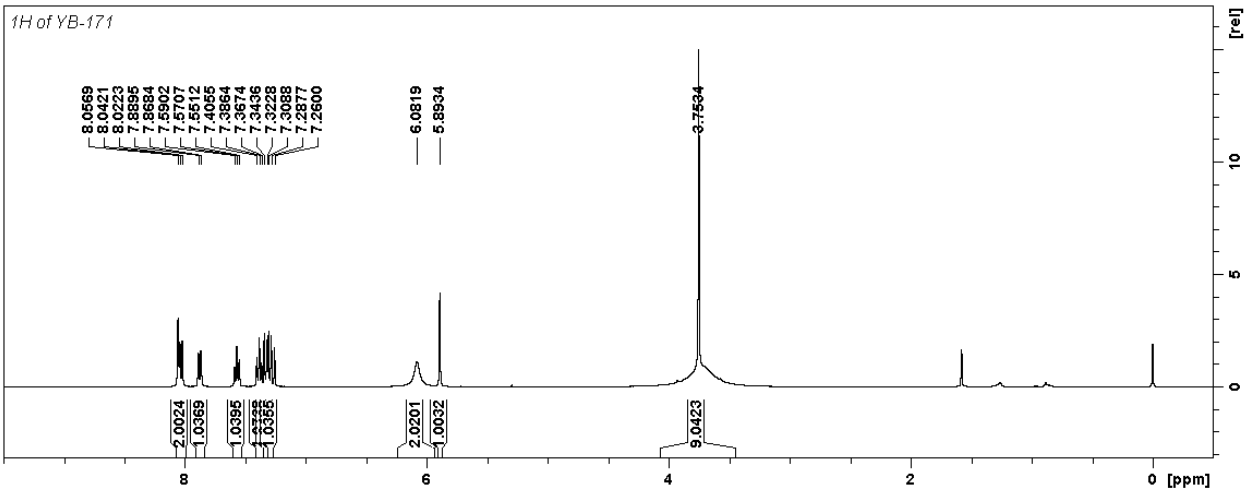

^{13}C NMR Spectra of 5k

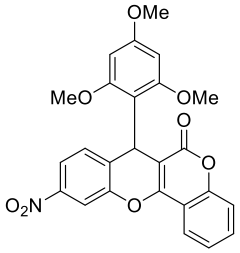

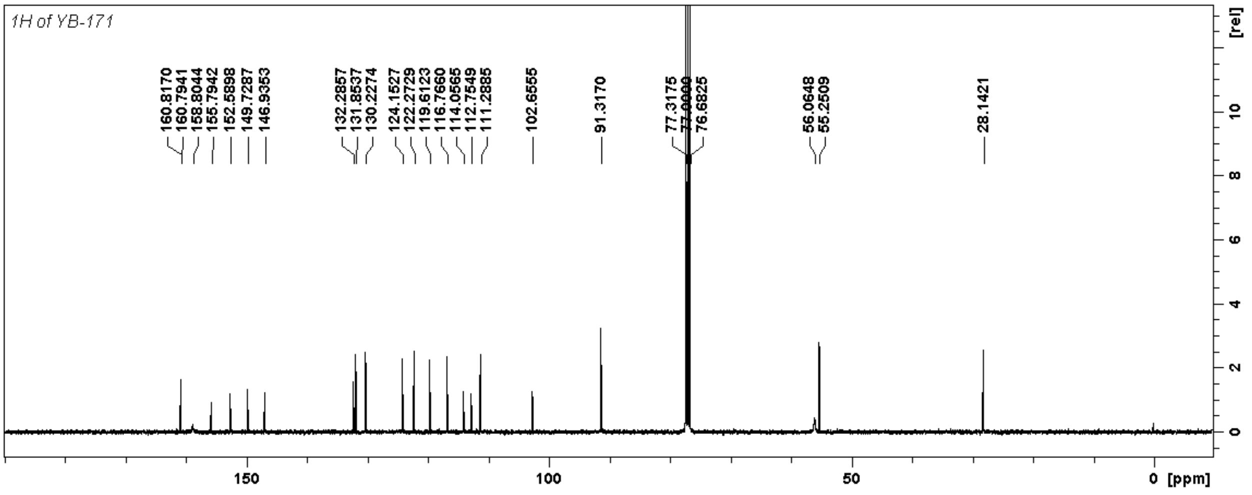

^{1}H NMR Spectra of 5l

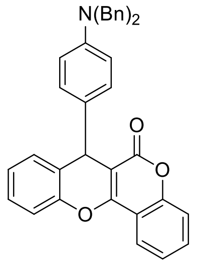

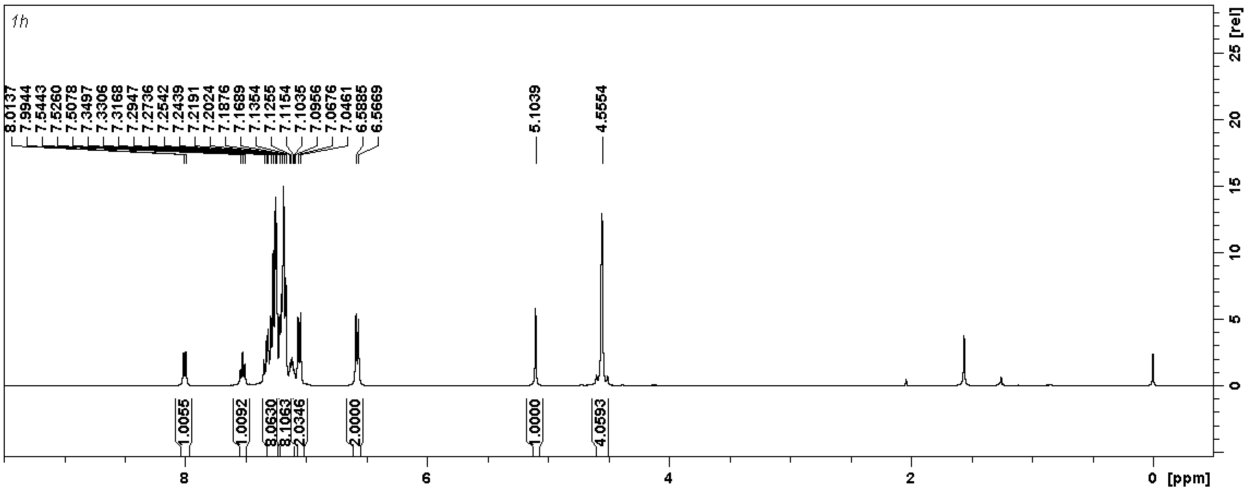

^{13}C NMR Spectra of 5l

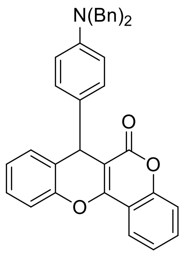

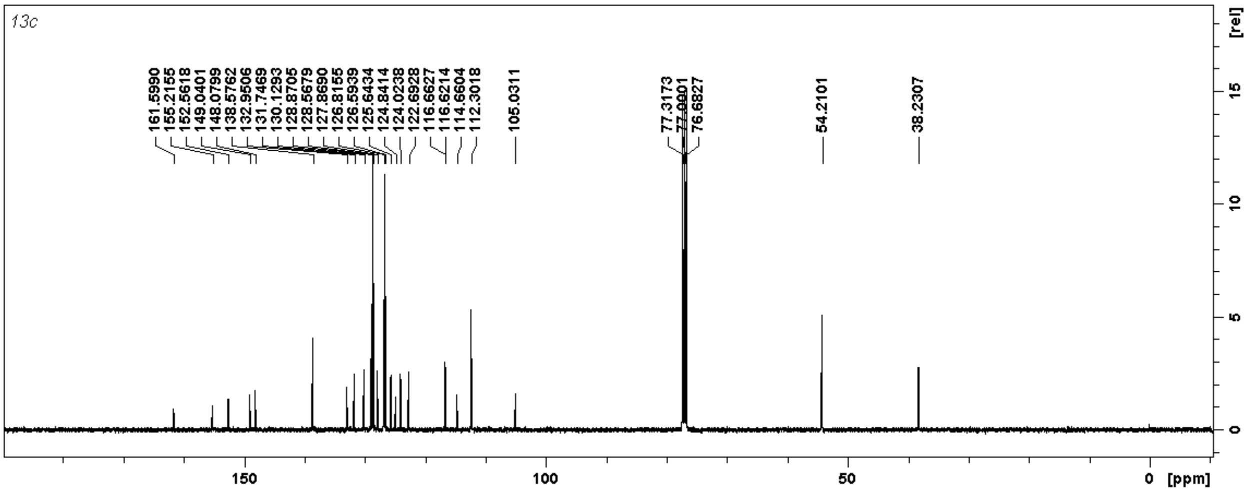

^{1}H NMR Spectra of 6

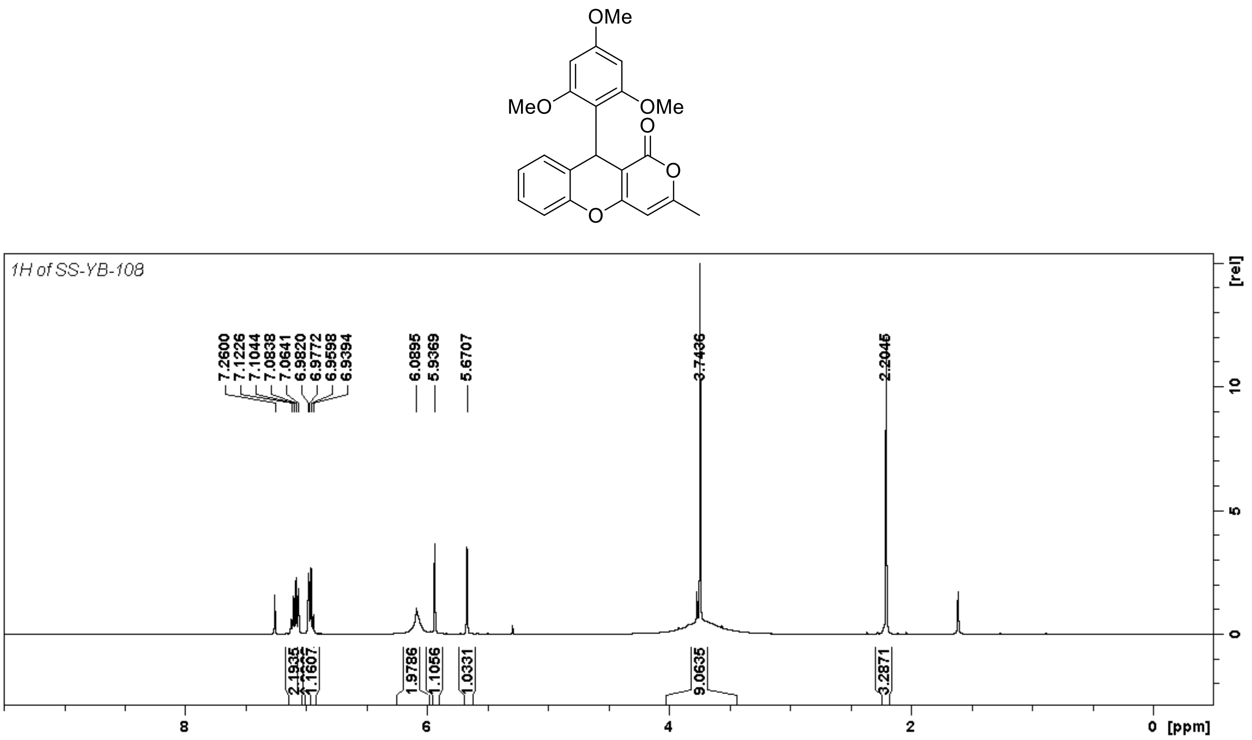

^{13}C NMR Spectra of 6

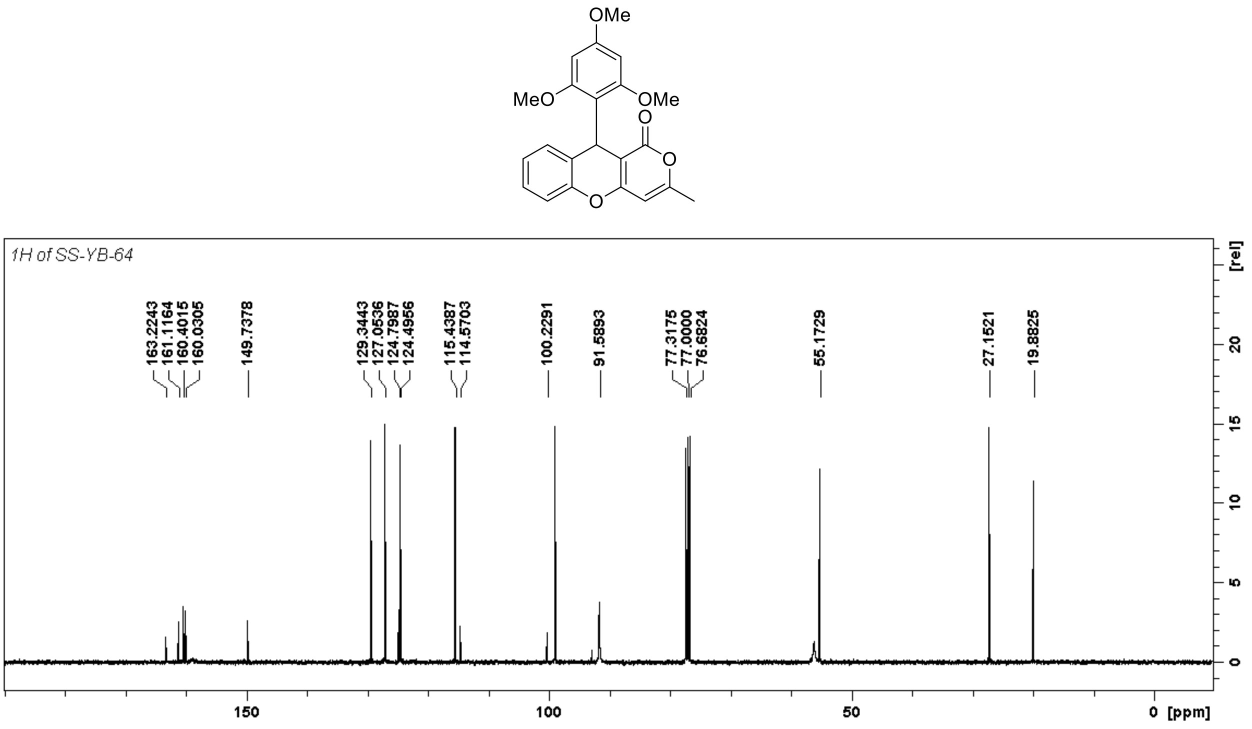

^{1}H NMR Spectra of B

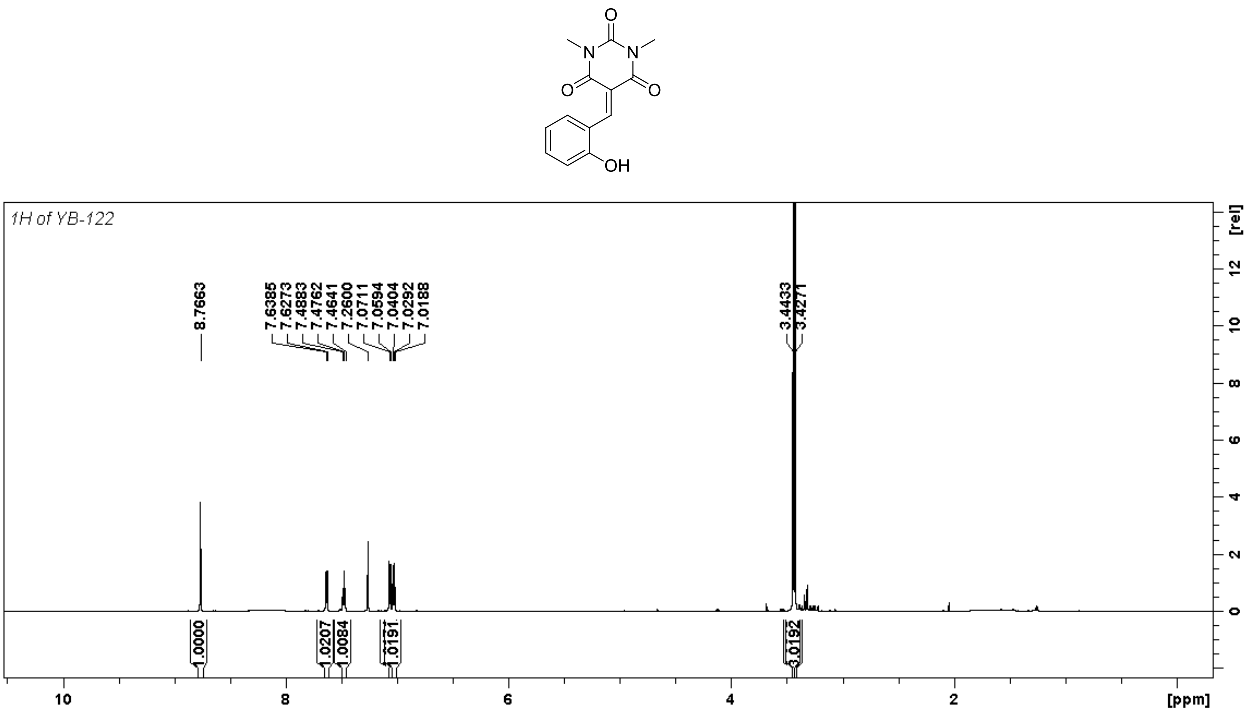

^{13}C NMR Spectra of B

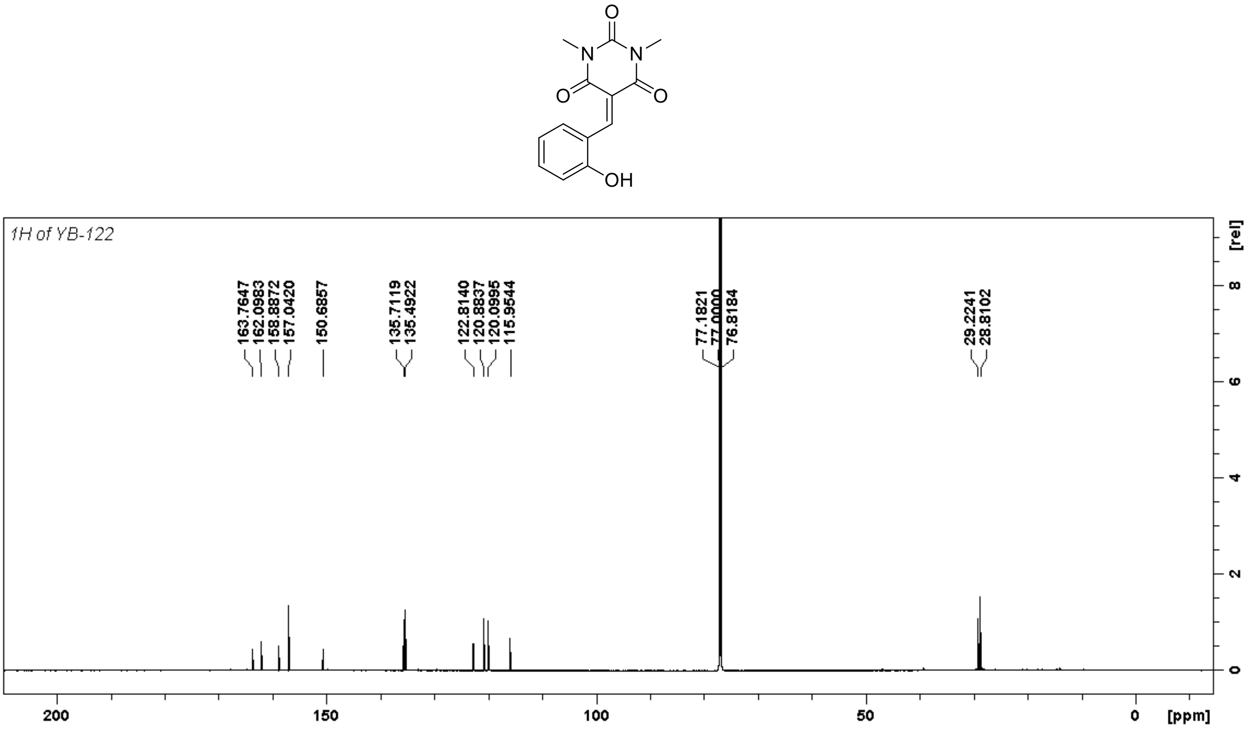

Supplement: RA-015-D5RA06198A-s001 [file RA-015-D5RA06198A-s001.pdf]
